# Development of Halogenated-Chalcones Bearing with Dimethoxy Phenyl Head as Monoamine Oxidase-B Inhibitors

**DOI:** 10.3390/ph15091152

**Published:** 2022-09-16

**Authors:** Nisha Abdul Rehuman, Jong Min Oh, Mohamed A. Abdelgawad, Eman A. M. Beshr, Mohammed A. S. Abourehab, Nicola Gambacorta, Orazio Nicolotti, Rakesh Kumar Jat, Hoon Kim, Bijo Mathew

**Affiliations:** 1 Department of Pharmaceutical Chemistry, Dr. Joseph Mar Thoma Institute of Pharmaceutical Sciences & Research, Kerala 690503, India; 2 Department of Pharmacy, Research Institute of Life Pharmaceutical Sciences, Sunchon National University, Suncheon 57922, Korea; 3 Department of Pharmaceutical Chemistry, College of Pharmacy, Jouf University, Sakaka 72341, Saudi Arabia; 4 Department of Medicinal Chemistry, Faculty of Pharmacy, Minia University, Minia 61519, Egypt; 5 Department of Pharmaceutics, College of Pharmacy, Umm Al-Qura University, Makkah 21955, Saudi Arabia; 6 Dipartimento di Farmacia—Scienze del Farmaco, Università degli Studi di Bari “Aldo Moro”, Via E. Orabona, 4, I-70125 Bari, Italy; 7 Department of Pharmaceutical Chemistry, JJTU University, Rajasthan 333001, India; 8 Department of Pharmaceutical Chemistry, Amrita School of Pharmacy, Amrita Vishwa Vidyapeetham, AIMS Health Sciences Campus, Kochi 682041, India

**Keywords:** dimethoxy chalcones, monoamine oxidase inhibitor, reversibility, cytotoxicity, molecular docking, Parkinson’s disease

## Abstract

Two series of dimethoxy-halogenated chalcones (**DM1**–**DM20**) were synthesized and tested for their ability to inhibit monoamine oxidase (MAOs). Compound **DM2** exhibited the most significant inhibition against MAO-B with an IC_50_ value of 0.067 µM, followed by compound **DM18** (IC_50_ = 0.118 µM), with selectivity index (SI) values of 93.88 and >338.98, respectively. However, none of the substances successfully inhibited MAO-A. The MAO-B inhibitors **DM2** and **DM18** were competitive and reversible, with K_i_ values of 0.032 ± 0.004 and 0.045 ± 0.001 µM, respectively. **DM2** was non-toxic below 100 µg/mL in the cytotoxic test using the Vero epithelial cell line by the MTT method. According to molecular docking studies, **DM2** and **DM18** formed very similar conformations within the MAO-B binding pocket, with the *ortho*-chlorine and *ortho*-fluorine aromatic rings sandwiched between F168 and Y326. These conformations were predicted to show better interactions with the targeted MAO-B than MAO-A. In particular, the induced-fit docking of the dimethoxy phenyl ring of **DM2** facing the hydrophobic pocket made up of FAD, Y398, and Y435 had an impact on F168 in the docking pocket. Taken together, **DM2** and **DM18** may be suitable candidates for treating neurodegenerative conditions such as Parkinson’s disease.

## 1. Introduction

Parkinson’s disease (PD) is a neurological condition that affects the patient’s ability to move about and regulate their muscles. The loss of striatal dopaminergic neurons causes motor symptoms of PD, and that of dopaminergic areas causes non-motor symptoms. Bradykinesia, muscle stiffness, and tremors are PD’s motor symptoms, whereas depression, cognitive problems, and sleep disorders are its motor symptoms [1,2,3,4]. PD is associated with risk factors and genetic mutations. Risk factors include free radical formation, oxidative stress, and environmental toxins [5,6,7,8,9]. Reduced motor function and disease-related clinical characteristics are caused by basal ganglia motor structure found in the extrapyramidal system, characterized by functional loss of dopaminergic neurons [10]. Other neurotransmitters, including cholinergic, serotonergic, adrenergic, and glutaminergic, are involved, but their participation is explained by non-motor characteristics [11,12,13,14,15,16].

Histopathological studies explain that the presence of Lewy bodies (α synuclein, ubiquitin, and other proteins) and loss of pigmented dopaminergic neurons are significant reasons. The degeneration of dopaminergic neurons in the substantia nigra pars compacta (SNpc), which project to the nigrostriatal pathway, is a substantial factor in the loss of dopaminergic function. In the extrapyramidal system, excitatory (D1) and inhibitory (D2) dopamine receptors are responsible for motor activity. Functional dopaminergic loss in PD patients results in the dysfunction of gamma-aminobutyric acid (GABA), and activity was stimulated in the internal globus pallidal segment (GPi) or pars reticulata portion of the substantia nigra (SNpr), which leads to the inhibition of thalamus. Decreased motor activity results from the diminished ability of the thalamus to switch on the frontal cortex [17,18].

Clinical manifestations explain that dopaminergic therapies activate D2 and D1 receptors, which restore dopamine activity and improve motor activity [19,20,21]. Levodopa has provided the best symptomatic relief since it was introduced. Dopamine agonists (DAGs), monoamine oxidase-B (MAO-B) inhibitors, and catechol-*O*-methyltransferase (COMT) inhibitors are used for the treatment of motor as well as non-motor symptoms [22,23,24,25]. MAO-B isoform in the human brain converts dopamine to homovanillic acid and 3,4-dihydroxyphenylacetic acid [26]. MAO-B also converts both exogenous and endogenous dopamine to hydrogen peroxide, responsible for oxidative damage and stress in PD. Thus, MAO-B inhibitors inhibit dopamine breakdown and enhance dopaminergic activity [27,28,29]. Inhibition of MAO-B reduces the free radicals resulting from the oxidation of dopamine.

Rasagiline, selegiline, and safinamide are the three MAO-B inhibitors used in the treatment of PD. Rasagiline and selegiline are under the category of selective, irreversible MAO-B inhibitors. Several studies evaluated that prolonged use of MAO-B inhibitors on non-motor symptoms of PD correlated with the development of hallucination and dementia [30,31,32]. PD Research Group of the United Kingdom (PDRG-UK) explains that combined therapy of selegiline and levodopa can develop cardiovascular side effects and orthostatic hypotension. At a higher dose of selegiline, MAO-B’s selectivity decreased, and MAO-A inhibition occurred [33,34,35]. The development of reversible MAO-B inhibitors needs considerable attention due to the pathogenesis of PD and the drawbacks of currently available MAO-B inhibitors.

Chalcones are the class of drug-likeness compounds that take part in various pharmacological profiles such as anti-inflammatory, antioxidant, hepatic-protective, anti-microbial, anti-cancer, and MAO inhibition [36,37]. Chalcones are moieties possessing open-chain flavonoids (1,3-diphenyl prop-2-ene-1-one (-CH=CH–CO-), in which the two aromatic or hetero rings are linked by the first and third carbon of the open chain [38]. Due to olefinic linkage, the structure occurs as trans and cis, where trans-chalcone is more stable than cis [39]. MAO-B Inhibitory properties of chalcones depend on the Michael acceptor’s electrophilic character and orientation of electron-donating (ED) and electron-withdrawing (EW) groups on the phenyl ring **A** or **B**. Numerous chalcones having an attachment with halogen, hydroxyl, and methoxy at different positions show a wide variety of pharmacological activities, such as MAO-B and cholinesterase inhibition [40,41,42,43,44,45,46,47].

Several studies have suggested that methoxy-chalcones are multi-targeting scaffolds for the expansion of multi-target ligands for the management of various neurodegenerative disorders [48,49,50,51,52,53]. The presence of various halogen atoms, such as fluorine, bromine, and chlorine on rings **A** and **B** of chalcone moiety showed highly selective MAO-B inhibition [54]. Therefore, in the current study, we planned to design the increased number of methoxy groups on ring **A** of chalcones with two positions of 2′,4′ and 3′,4′, respectively. Moreover, different halogens were placed in various positions of phenyl ring **B** in order to understand the fine-tuning of halogen orientation. The lead molecules were subjected to kinetics, reversibility, cytotoxic evaluation, and docking analysis [https://doi.org/10.3390/chemistry4030067 (accessed on 6 September 2022)].

## 2. Results and Discussion

### 2.1. Synthesis

**Figure 1:**
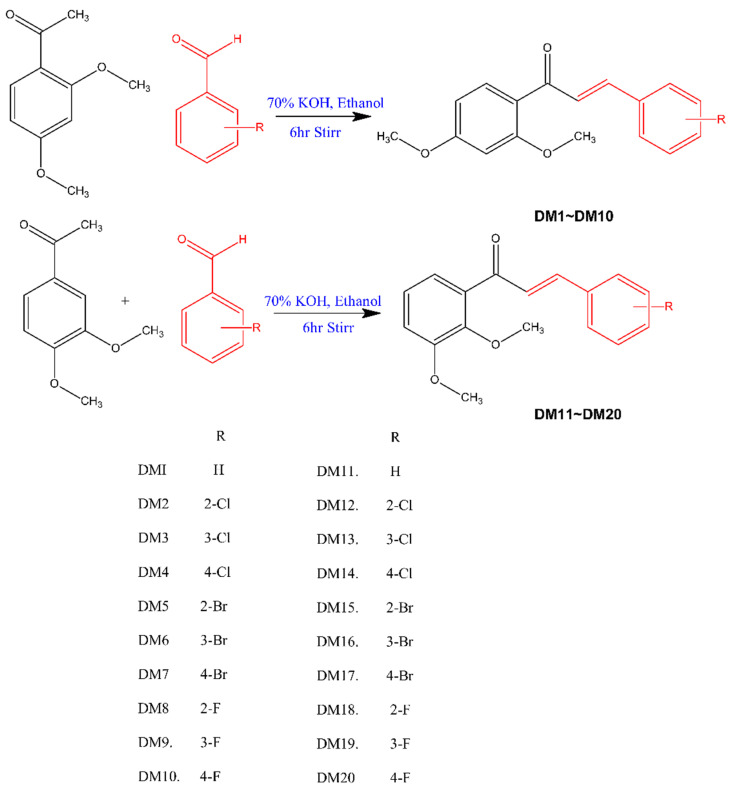
Synthetic route of dimethoxy halogenated chalcones.

By using Claisen–Schmidt condensation, 2,4-dimethoxy benzaldehyde and 3,4-dimethoxy benzaldehyde were condensed with different substituted halogenated benzaldehydes to synthesize dimethoxy halogenated chalcones [55]. The synthetic route is described in Figure 1. The spectral data of the compounds were provided in the Supplementary Materials.

### 2.2. MAO Inhibition Assays

The present study documented that when compared to MAO-A, two series of dimethoxylated chalcones inhibited MAO-B more potently, and they all had potent MAO-B inhibitory actions with the residual activity of less than 50% at 10 µM (Table 1). In general, 2′,4′-dimethoxy chalcone derivatives (**DM1**–**DM10**) showed more effective inhibitory activities against MAO-B, compared to 3′,4′-dimethoxy chalcones derivatives (**DM11**–**DM20**) (Table 1). Compound **DM2** most potently inhibited MAO-B with an IC_50_ value of 0.067 μM, followed by **DM18**, **DM3**, **DM6**, **DM17**, and **DM5** (IC_50_ = 0.118, 0.130, 0.148, 0.146, and 0.161 μM, respectively). The -Cl atom on *ortho* position (**DM2**) increased MAO-B inhibitory activity compared to the parental compound **DM1**.

**Figure 2:**
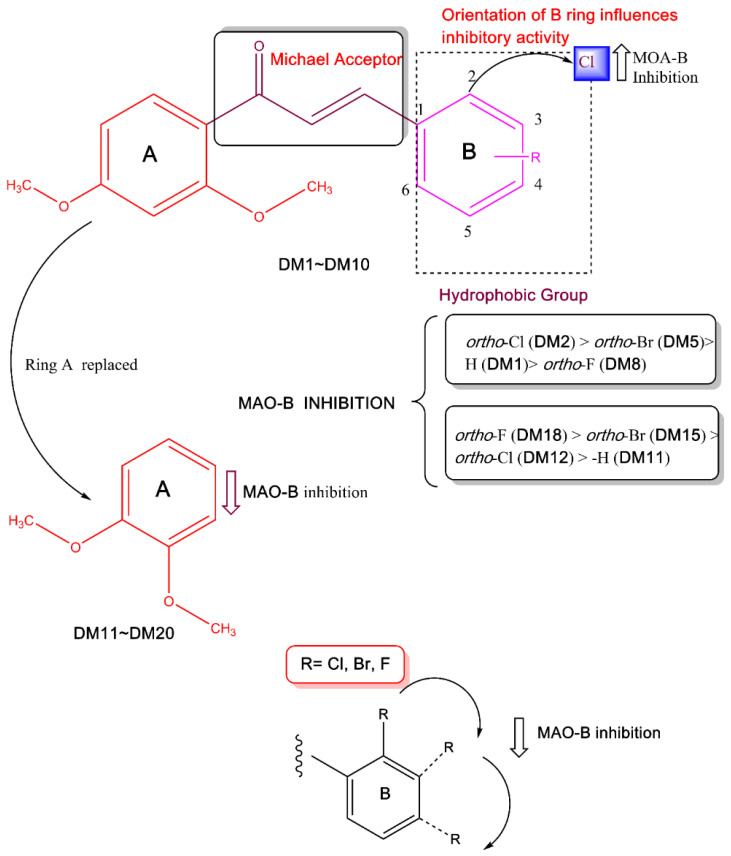
SAR of dimethoxylated halogen-containing chalcones.

However, the -Br or -F atoms on the *ortho* position (**DM5** and **DM8**, respectively) improved the MAO-B inhibitory profile less than the -Cl atom in that respective position. MAO-B inhibitory activities increased with *ortho*-Cl (**DM2**) > *ortho*-Br (**DM5**) > -H (**DM1**) > *ortho*-F (**DM8**). In addition, -Cl atom on *ortho* position (**DM2**) was effective on MAO-B inhibitory activity compared to *meta* and *para* positions (**DM3** and **DM4**, respectively) (Table 1). On the other hand, the -F atom on *ortho* position in **DM18** increased MAO-B inhibitory activity compared to the parental structure **DM11**. Contrarily, the -Cl and -Br atom on *ortho* position in **DM12** and **DM15** showed low MAO-B inhibitory activity. In these derivatives, *ortho*-F showed higher MAO-B inhibition (**DM18**) than *ortho*-Br (**DM15**), *ortho*-Cl (**DM12**), and -H (**DM11**). In **DM18**, -F atom on *ortho* position inhibited MAO-B 3.75 and four times more, respectively, than -F in the *meta* and *para* positions of **DM19** and **DM20** (Table 1). **DM2** and **DM18** were selective for MAO-B with selectivity index (SI) values of 93.88 and >338.98, respectively, over MAO-A, suggesting that **DM2** has the highest inhibitory activity, and **DM18** has extremely high selectivity for MAO-B (Table 1). Structure–activity relationship (SAR) study of the synthesized compounds showed that the orientation of the halogen atoms in ring **B** at different locations affected MAO-B inhibition. The current study indicated that the *ortho* location of the phenyl **B** ring’s chlorine group had a stronger MAO-B inhibiting effect with an IC_50_ value of 0.067 µM. Figure 2 shows some crucial SARs of dimethoxylated chalcones. In additional experiments for multi-targeting tests, all compounds weakly inhibited acetylcholinesterase, butyrylcholinesterase, and β-secretase-1 (Table S1).

Our design strategy mainly focused on the di-methoxy group in various positions of the second, third, and fourth of ring **A** of chalcones. The 2′,4′- and 3′,4′-dimethoxy substitutions are the only two possible pharmacophores we can generate from this aspect. It is obvious from the selectivity pattern that 3′,4′-dimethoxy substituted chalcones have no effect on the MAO-A inhibition. This theory postulated that the positioning of two methoxy groups separated by an aromatic carbon would maximize the selectivity of MAO-B (**DM1**–**DM10**). The 3′,4′-dimethoxy substituted chalcones in close proximity (**DM11**–**DM20**) demonstrated strong MAO-B inhibition and weak MAO-A activity.

### 2.3. Kinetic Study

**Figure 3:**
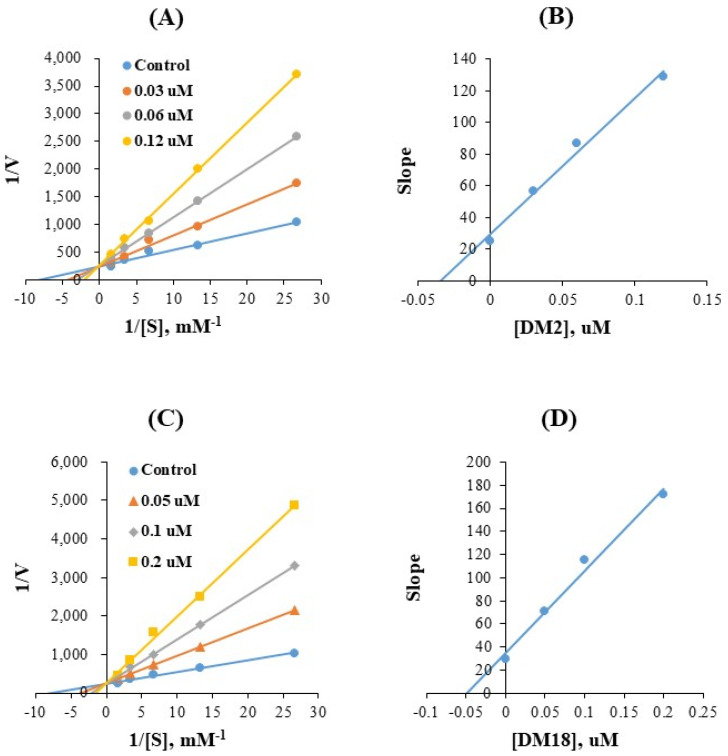
LB plots for MAO-B inhibition by **DM2** and **DM18** (**A**,**C**), and their secondary plots (**B**,**D**) of the slopes vs. inhibitor concentrations.

Lineweaver-Burk (LB) plots revealed that **DM2** and **DM18** were competitive inhibitors of MAO-B in the kinetic investigations of MAO-B (Figure 3A,C), and their secondary plots revealed that their K_i_ values were 0.032 ± 0.004 and 0.045 ± 0.001 µM, respectively (Figure 3B,D). These findings imply that **DM2** and **DM18** are competitive inhibitors that bind to the MAO-B active site together with the substrate.

### 2.4. Reversibility Studies

**Figure 4:**
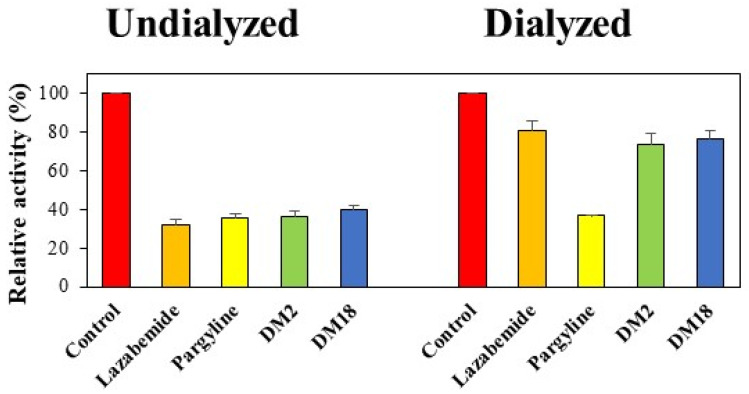
Recoveries of MAO-B inhibitions by **DM2** and **DM18** using dialysis experiments.

The concentrations used in the tests were 0.13 µM for **DM2**, 0.24 µM for **DM18**, 0.22 µM for lazabemide (a reference reversible inhibitor), and 0.28 µM for pargyline (a reference irreversible inhibitor). To identify the reversibility patterns, the relative activities for samples dialyzed (A_D_) and for those undialyzed (A_U_) were compared. The MAO-B inhibitions by **DM2** and **DM18** were restored from 36.0% (A_U_) to 74.0% (A_D_) and from 40.1% to 76.6%, respectively (Figure 4). These recovery values could be distinguished from those of pargyline, an irreversible reference inhibitor against MAO-B, which ranged from 35.5% to 36.8%, and those of lazabemide, a reversible reference inhibitor against MAO-B, which ranged from 31.7% to 80.9%. These findings demonstrated that **DM2** and **DM18** were reversible MAO-B inhibitors.

### 2.5. In Vitro Toxicity Evaluation

**Figure 5:**
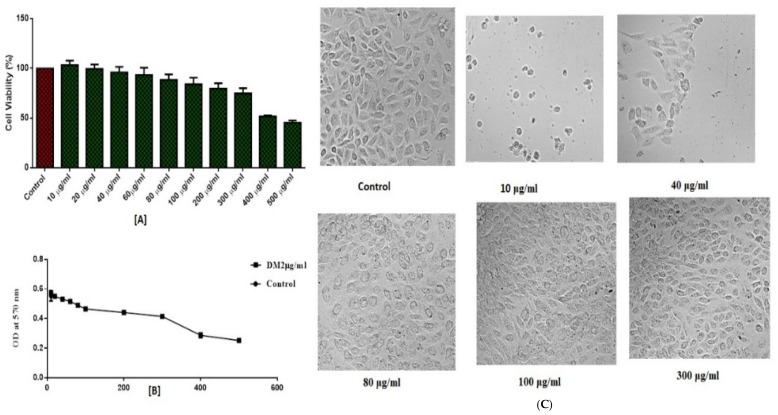
Effect of **DM2** on Vero cell viability. (**A**) Cell viability; (**B**) A dose-response curve and IC_50_; (**C**) Morphological observation of Vero cells with different concentrations under phase contrast microscope after 48 h exposure.

On the normal epithelial cells isolated from the kidney of an African monkey called Vero, the MTT test was carried out to demonstrate the biological safety of the compound **DM2**. Vero cells were treated to various doses (10–500 µg/mL) for 48 h, and relative cell viability was determined by measuring the absorbance at 570 nm with an ELISA microplate. The results demonstrated that cell toxicity varied with concentration and that at a concentration of 100 µg/mL, 80% of cells were alive (Figure 5A). The IC_50_ value for **DM2** was determined to be 183.2 µg/mL using the GraphPad Prism 6.0 program (Figure 5B). At a greater dosage of 300 µg /mL, cytotoxicity was evident in cell shrinkage and blebbing, and cellular density decreased (Figure 5C). The experiments found that **DM2** did not become harmful to Vero cells below 100 µg/mL dosage.

### 2.6. Computational Studies

In silico analysis was performed in an attempt to inspect the binding mode of compounds **DM2** and **DM18** towards the MAO-B binding pocket.

**Figure 6:**
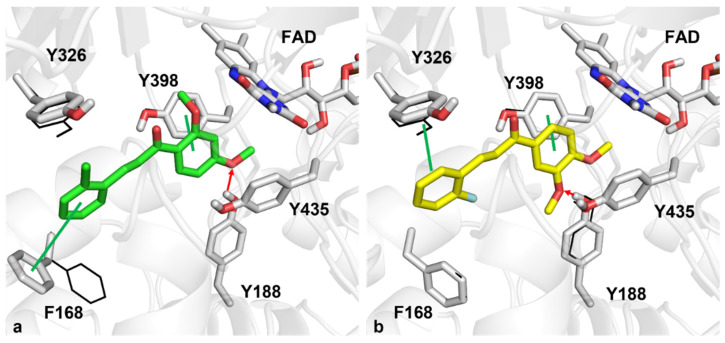
Docking analysis of **DM2** (**a**) and **DM18** (**b**) for the best poses towards MAO-B. Green and yellow sticks represent **DM2** and **DM18**, respectively. Black wireframes depict MAO-B side chains in the original positions. Green lines and red arrows indicate Pi-Pi contacts and hydrogen bonds, respectively.

As far as **DM2** interactions are concerned, the ortho-chlorine aromatic ring can engage in Pi-Pi interaction with F168, interacting through hydrophobic contact with Y326 MAO-B selective residue. Furthermore, the 2,4-dimethoxy phenyl ring lies in the aromatic pocket formed by FAD, Y398, and Y435, establishing a Pi-Pi interaction with Y398. In addition, the methoxy substituent can make a hydrogen bond with the side chain of Y188. Regarding **DM18**, the 3,4-dimethoxy impacts the aromatic region formed by FAD, Y435, and Y398, making Pi-Pi contact with the latter. In addition, the methoxy substituent can make a hydrogen bond with Y188, as well as the **DM2** compound. The ortho-fluorine aromatic ring also makes a Pi-Pi contact with Y326 (Figure 6). For the sake of completeness, docking score values obtained from the induced-fit docking simulations were equal to −10.30 and −10.17 kcal/mol for compounds **DM2** and **DM18**, respectively, very similar to the value computed for the MAO-B cognate ligand described in the Materials and Methods section.

## 3. Materials and Methods

### 3.1. Synthesis

In the presence of 70% KOH as a catalyst, dimethoxy benzaldehyde (0.01 mol) and substituted benzaldehyde (0.01 mol) were dissolved in 50 mL of ethanol and stirred for 6 h at room temperature. After being transferred to water, the solution was acidified with 10% HCl, filtered, washed with water, and dried. In order to obtain pure crystals, the product was recrystallized using ethanol [46]. Through the use of thin layer chromatography (10:90 ethylacetate to hexane ratio), the compounds’ purities were examined. The Supplementary Materials contains the spectral data.

### 3.2. MAO Assays

Recombinant MAO-A and MAO-B were used to test the MAO inhibitory activity using the substrates kynuramine and benzylamine (0.06 mM and 0.3 mM, respectively) [56]. Lazabemide and pargyline were employed as reference drugs for MAO-B, whereas toloxatone and clorgyline were used for MAO-A. For MAO-B, K_m_ of benzylamine in this study was 0.13 mM. Chemicals were from Sigma-Aldrich [57].

### 3.3. Kinetics Studies

MAO-A At 10 µM, compounds’ inhibitory effects on MAOs were initially recorded. The IC50 values of the compounds were calculated for those that had residual activity that were close to or below 50% [58]. The SI values of MAO-B were calculated by dividing the IC_50_ values for MAO-A and those for MAO-B. The MAO-B was used to study the kinetics of substances **DM2** or **DM18** at five different substrate concentrations. By using the LB plots and their secondary plots at three different inhibitor doses, the inhibition patterns were examined.

### 3.4. Inhibition Reversibility of **DM2** and **DM18**

Following a 30 min preincubation with MAO-B and **DM2** or **DM18** at ~2 × IC_50_ (i.e., 0.13 and 0.24 µM, respectively), dialysis was performed to assess the reversibility of MAO-B inhibition [59]. To serve as benchmarks, MAO-B was preincubated with 0.22 µM of lazabemide (a reference reversible MAO-B inhibitor) or 0.28 µM of pargyline (a reference irreversible MAO-B inhibitor). By contrasting the recovery results of samples that had been dialyzed (A_D_) and undialyzed (A_U_), reversibility patterns were determined.

### 3.5. Cytotoxicity Study

Cytotoxicity study of the lead molecule **DM2** was carried out as previously described [60,61] and as in Supplementary Materials described shortly.

### 3.6. Computational Studies

The crystal structure of MAO-B was downloaded from the Protein Data Bank by selecting entry 2V5Z [62]. The docking protocol was described in our previous studies [63]. For completeness, with the purpose of validating the docking protocol, redocking analysis was performed. Satisfactorily, the root mean square deviation (RMSD) of the best MAO-B cognate ligand docked pose was equal to 0.390 Å with respect to the experimental pose and returned a docking score equal to −10.76 kcal/mol.

## 4. Conclusions

In this study, two series of twenty dimethoxy-chalcones (**DM1**–**DM20**) with different substituted halogens were synthesized and examined for their ability to inhibit MAOs. All twenty molecules showed greater MAO-B inhibitory action than MAO-A. Chalcones have a significant impact on the MAO-B inhibitory actions depending on the kind and orientation of the halogen atoms on the **B** phenyl ring of the chalcone. The two sets greatly influenced MAO-B inhibition by substituting halogens at different locations on the phenyl ring **B**, notably at the ortho position. Compound **DM2** most potently inhibited MAO-B with an IC_50_ value of 0.067 μM, followed by **DM18**, **DM3**, **DM6**, **DM17**, and **DM5** (IC_50_ = 0.118, 0.130, 0.148, 0.146, and 0.161 μM, respectively). Relative activities for undialyzed (A_U_) and dialyzed (A_D_) were compared, and inhibition of MAO-B by **DM2** and **DM18** was recovered, similar to the reversible reference inhibitor against MAO-B. The results documented that **DM2** and **DM18** were reversible inhibitors of MAO-B. The biological safety of **DM2** was evaluated by cytotoxic evaluations at different concentrations, and it was found that **DM2** was not harmful to Vero cells below a dosage of 100 µg/mL. Induced-fit docking simulations gave a robust and detailed explanation of the binding mode of compounds **DM2** and **DM18** towards MAO-B. In particular, the two compounds can be assumed to have very similar conformations within the MAO-B binding pocket, with the ortho-chlorine and *ortho*-fluorine aromatic rings sandwiched between Y326 and F168, and the latter particularly was affected by the induced-fit docking of compound **DM2**, and the dimethoxy phenyl rings faced the aromatic pocket consisting of FAD, Y398, and Y435. Finally, the presence of hydrogen bonds with the side chain of Y188 can stabilize the two compounds within the binding pocket. The study concluded that the **DM2** and **DM18** molecules from this series could be considered candidates for the development of a new class of MAO-B inhibitors for the treatment of PD.

## Figures and Tables

**Table 1:** Inhibitions of MAO-A and MAO-B by DM series ^a^.

Compounds	Residual Activity at 10 µM (%)		IC_50_ (µM)		SI ^b^
	MAO-A	MAO-B	MAO-A	MAO-B	
**DM1**	57.23 ± 1.91	17.56 ± 0.74	15.393 ± 1.969	0.927 ± 0.021	16.61
**DM2**	41.53 ± 1.59	−2.85 ± 1.02	6.293 ± 0.432	0.067 ± 0.002	93.88
**DM3**	43.27 ± 1.20	7.89 ± 0.63	5.733 ± 0.142	0.130 ± 0.023	44.08
**DM4**	38.77 ± 3.40	18.59 ± 1.04	4.482 ± 0.152	0.589 ± 0.055	7.61
**DM5**	54.52 ± 4.32	10.57 ± 1.88	12.383 ± 0.872	0.161 ± 0.020	77.02
**DM6**	62.70 ± 1.32	5.50 ± 0.64	12.755 ± 1.569	0.148 ± 0.087	86.49
**DM7**	40.28 ± 1.05	15.55 ± 1.78	4.733 ± 0.005	0.844 ± 0.023	5.60
**DM8**	56.35 ± 7.34	11.10 ± 1.44	13.420 ± 0.820	1.126 ± 0.033	11.90
**DM9**	50.58 ± 0.82	10.77 ± 0.23	11.252 ± 1.057	0.246 ± 0.074	45.93
**DM10**	53.91 ± 7.17	23.22 ± 0.37	16.037 ± 1.467	1.965 ± 0.065	8.14
**DM11**	64.61 ± 0.72	14.58 ± 3.48	14.293 ± 1.829	2.188 ± 0.098	6.54
**DM12**	88.70 ± 9.40	8.98 ± 0.92	>40	1.225 ± 0.250	>32.65
**DM13**	75.63 ± 0.88	7.49 ± 0.12	>40	4.700 ± 0.320	>8.51
**DM14**	98.75 ± 1.77	9.45 ± 2.88	>40	0.833 ± 0.087	>48.02
**DM15**	77.50 ± 7.07	10.25 ± 0.17	>40	0.716 ± 0.056	>55.87
**DM16**	76.88 ± 0.88	11.83 ± 5.27	>40	1.113 ± 0.260	>35.94
**DM17**	96.88 ± 0.88	13.76 ± 0.61	>40	0.146 ± 0.041	>273.97
**DM18**	100.00 ± 0.01	8.87 ± 1.08	>40	0.118 ± 0.036	>338.98
**DM19**	93.67 ± 7.16	3.94 ± 1.76	>40	0.450 ± 0.071	>88.89
**DM20**	97.47 ± 8.95	3.70 ± 0.00	>40	0.483 ± 0.077	>83.82
Toloxatone			1.080 ± 0.025	-	
Lazabemide			-	0.110 ± 0.016	
Clorgyline			0.007 ± 0.001	-	
Pargyline			-	0.140 ± 0.006	

^a^ Results are the means ± standard errors of duplicate or triplicate experiments. ^b^ Selectivity index (SI) values are expressed for MAO-B as compared to MAO-A.

## Data Availability

Data are contained within the article and Supplementary Materials.

## Supplementary Materials

The following supporting information can be downloaded at: https://www.mdpi.com/article/10.3390/ph15091152/s1, Figure S1: ^1^H-NMR spectra, Figure S2: ^13^C-NMR spectra, Figure S3: Mass spectra, Text S1: Supplementary procedure for toxicity evaluation, Table S1: Inhibitions of AChE, BChE, and BACE-1 by **DM** series. Refs. [64,65,66,67] are cited in Supplementary Materials.
