# Supplementary material for: Development of Halogenated-Chalcones Bearing with Dimethoxy Phenyl Head as Monoamine Oxidase-B Inhibitors
Source: Pharmaceuticals (Basel). 2022 Sep 16;15(9):1152. doi: 10.3390/ph15091152 (PMC9503132; doi:10.3390/ph15091152)
Supplement: Supplementary file 1 [file pharmaceuticals-15-01152-s001.zip › pharmaceuticals-1893545-supplementary.pdf]

Supplementary Materials

Development of Halogenated-Chalcones Bearing with Dimethoxy Phenyl Head as Monoamine Oxidase-B Inhibitors

Nisha Abdul Rehuman [1†], Jong Min Oh [2†], Mohamed A Abdelgawad [3], Eman A. M. Beshr [4], Mohammed A. S. Abourehab [5], Nicola Gambacorta [6], Orazio Nicolotti [6], Rakesh Kumar Jat [7], Hoon Kim [2*] and Bijo Mathew [8*]

[1] Department of Pharmaceutical Chemistry, Dr. Joseph Mar Thoma Institute of Pharmaceutical Sciences & Research, Kerala 690503, India
[2] Department of Pharmacy, and Research Institute of Life Pharmaceutical Sciences, Sunchon National University, Suncheon 57922, Republic of Korea
[3] Department of Pharmaceutical Chemistry, College of Pharmacy, Jouf University, Sakaka, 72341, Saudi Arabia
[4] Department of Medicinal Chemistry, Faculty of Pharmacy, Minia University, Minia 61519, Egypt
[5] Department of Pharmaceutics, College of Pharmacy, Umm Al-Qura University, Makkah, 21955 Saudi Arabia
[6] Dipartimento di Farmacia—Scienze del Farmaco, Università degli Studi di Bari "Aldo Moro", Via E. Orabona, 4, I-70125 Bari, Italy
[7] Department of Pharmaceutical Chemistry, JJTU University, Rajasthan 333001, India
[8] Department of Pharmaceutical Chemistry, Amrita School of Pharmacy, Amrita Vishwa Vidyapeetham, AIMS Health Sciences Campus, Kochi 682041, India
* Correspondence: hoon@sunchon.ac.kr (H. K); bijomathew@aims.amrita.edu or bijovilaventgu@gmail.com) (B. M.)
† These authors contributed equally to this work.

Contents

Figure S1. ^{1}H-NMR spectra ………………………………………………………………….. 2

Figure S2. ^{13}C-NMR spectra ……………………………………………………..…….……….. 11

Figure S3. MASS Spectra ……………………………………………………………….….… 20

Text S1. Supplementary procedure for toxicity evaluation …………………………….…….. 29

Table S1. Inhibitions of AChE, BChE, and BACE-1 by **DM** series ……………………..…….. 30

Figure S1. ^{1}H-NMR spectra

D1
1H_8scan CDCl3 {D:\Spectra} nmr 13

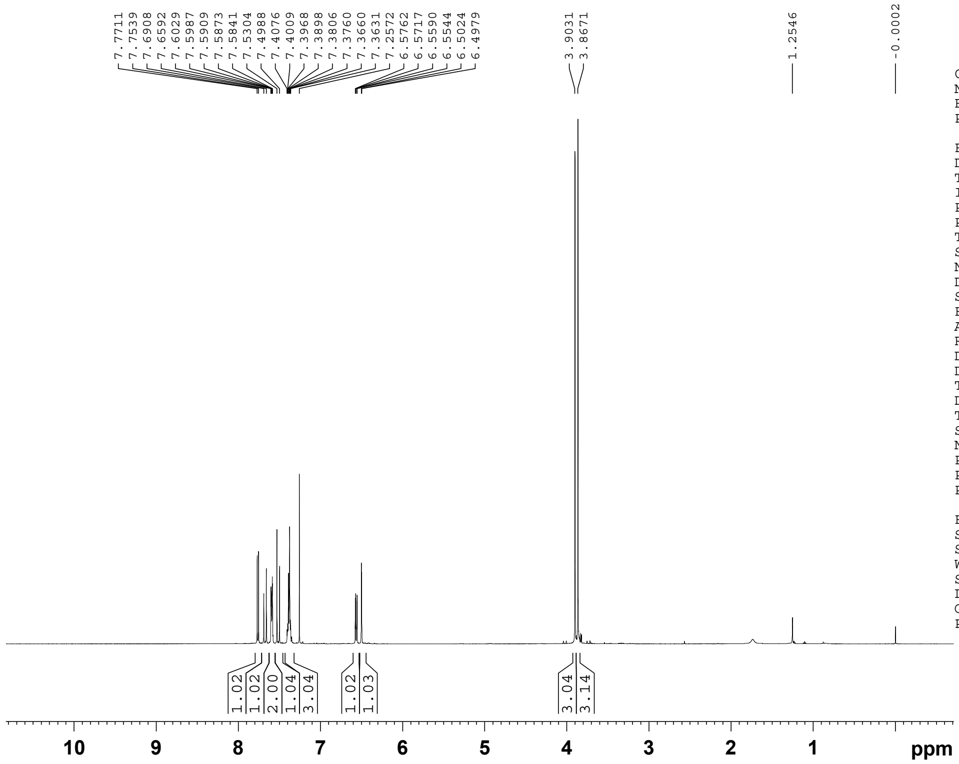

BRUKER
AVANCE NEO
500 MHz NMR
SPECTROMETER
SAIF, P.U.

Current Data Parameters

NAME	Dec15-2020
EXPNO	130
PROCNO	1

F2 - Acquisition Parameters

Date_	20201215
Time	13.42 h
INSTRUM	Avance Neo 500
PROBHD	Z119470_0333 (
PULPROG	zg30
TD	65536
SOLVENT	CDCl3
NS	8
DS	0
SWH	14705.883 Hz
FIDRES	0.448788 Hz
AQ	2.2282240 sec
RG	95.7854
DW	34.000 usec
DE	6.79 usec
TE	300.2 K
D1	1.00000000 sec
TD0	1
SFO1	500.1730885 MHz
NUC1	1H
P0	3.33 usec
P1	10.00 usec
PLW1	20.93000031 W

F2 - Processing parameters

SI	65536
SF	500.1700133 MHz
WDW	EM
SSB	0
LB	0.30 Hz
GB	0
PC	1.00

D2
1H_8scan CDCl3 {D:\Spectra} nmr 14

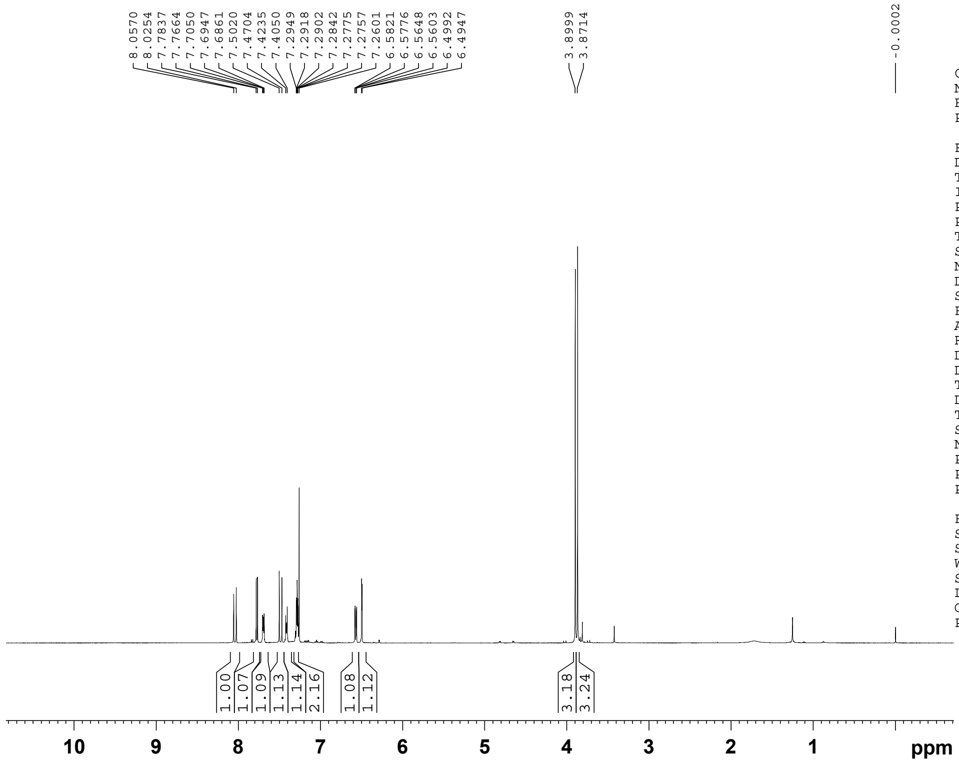

BRUKER
AVANCE NEO
500 MHz NMR
SPECTROMETER
SAIF, P.U.

Current Data Parameters

NAME	Dec15-2020
EXPNO	140
PROCNO	1

F2 - Acquisition Parameters

Date_	20201215
Time	13.44 h
INSTRUM	Avance Neo 500
PROBHD	Z119470_0333 (
PULPROG	zg30
TD	65536
SOLVENT	CDCl3
NS	8
DS	0
SWH	14705.883 Hz
FIDRES	0.448788 Hz
AQ	2.2282240 sec
RG	95.7854
DW	34.000 usec
DE	6.79 usec
TE	300.2 K
D1	1.00000000 sec
TD0	1
SFO1	500.1730885 MHz
NUC1	1H
P0	3.33 usec
P1	10.00 usec
PLW1	20.93000031 W

F2 - Processing parameters

SI	65536
SF	500.1700119 MHz
WDW	EM
SSB	0
LB	0.30 Hz
GB	0
PC	1.00

D3
1H_8scan CDCl3 {D:\Spectra} nmr 15

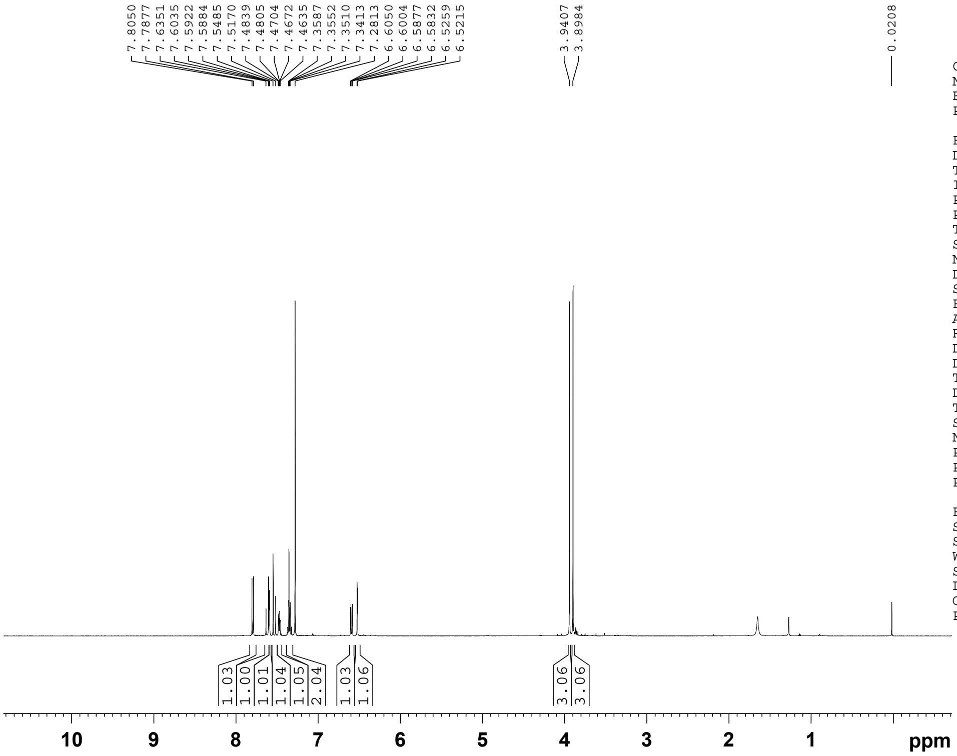

BRUKER
AVANCE NEO
500 MHz NMR
SPECTROMETER
SAIF, P.U.

Current Data Parameters

NAME	Dec15-2020
EXPNO	150
PROCNO	1

F2 - Acquisition Parameters

Date_	20201215
Time	13.47 h
INSTRUM	Avance Neo 500
PROBHD	Z119470_0333 (
PULPROG	zg30
TD	65536
SOLVENT	CDCl3
NS	8
DS	0
SWH	14705.883 Hz
FIDRES	0.448788 Hz
AQ	2.2282240 sec
RG	101
DW	34.000 usec
DE	6.79 usec
TE	300.2 K
D1	1.00000000 sec
TD0	1
SFO1	500.1730885 MHz
NUC1	1H
P0	3.33 usec
P1	10.00 usec
PLW1	20.93000031 W

F2 - Processing parameters

SI	65536
SF	500.1700012 MHz
WDW	EM
SSB	0
LB	0.30 Hz
GB	0
PC	1.00

D5
1H_8scan CDCl3 {D:\Spectra} nmr 17

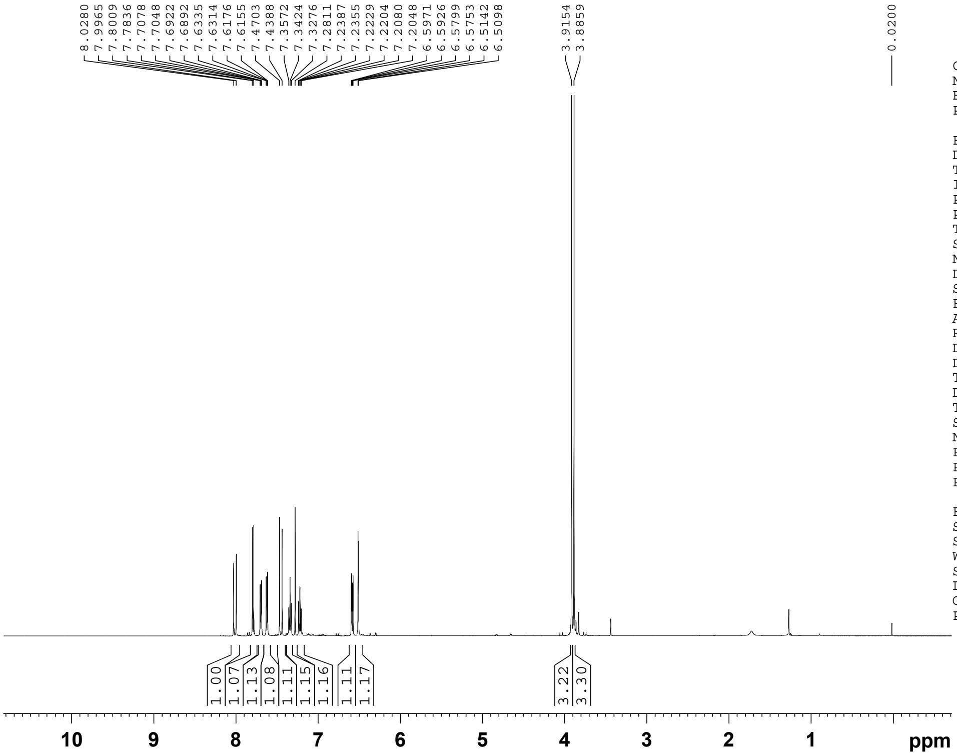

BRUKER
AVANCE NEO
500 MHz NMR
SPECTROMETER
SAIF, P.U.

Current Data Parameters

NAME	Dec15-2020
EXPNO	170
PROCNO	1

F2 - Acquisition Parameters

Date_	20201215
Time	13.52 h
INSTRUM	Avance Neo 500
PROBHD	Z119470_0333 (
PULPROG	zg30
TD	65536
SOLVENT	CDCl3
NS	8
DS	0
SWH	14705.883 Hz
FIDRES	0.448788 Hz
AQ	2.2282240 sec
RG	95.7854
DW	34.000 usec
DE	6.79 usec
TE	300.2 K
D1	1.00000000 sec
TD0	1
SFO1	500.1730885 MHz
NUC1	1H
P0	3.33 usec
P1	10.00 usec
PLW1	20.93000031 W

F2 - Processing parameters

SI	65536
SF	500.1700012 MHz
WDW	EM
SSB	0
LB	0.30 Hz
GB	0
PC	1.00

D6

1H_8scan CDCl3 {D:\Spectra} nmr 18

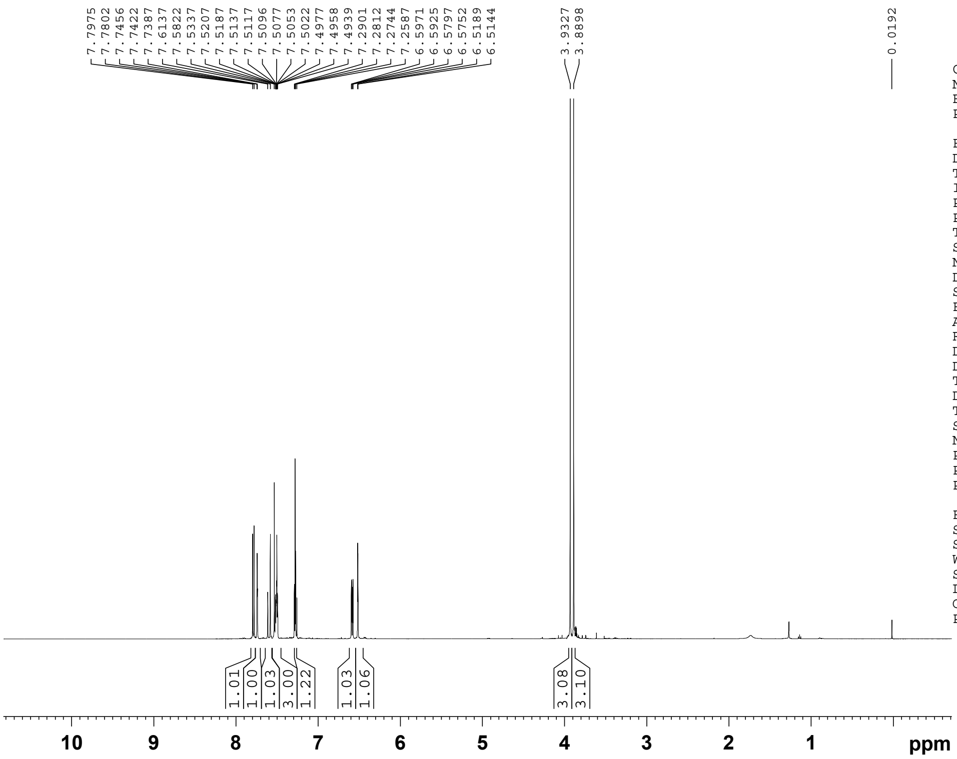

BRUKER
AVANCE NEO
500 MHz NMR
SPECTROMETER
SAIF, P.U.

Current Data Parameters
NAME Dec15-2020
EXPNO 180
PROCNO 1

F2 - Acquisition Parameters
Date_ 20201215
Time 13.55 h
INSTRUM Avance Neo 500
PROBHD Z119470_0333 (
PULPROG zg30
TD 65536
SOLVENT CDCl3
NS 8
DS 0
SWH 14705.883 Hz
FIDRES 0.448788 Hz
AQ 2.2282240 sec
RG 95.7854
DW 34.000 usec
DE 6.79 usec
TE 300.2 K
D1 1.00000000 sec
TD0 1
SFO1 500.1730885 MHz
NUC1 1H
P0 3.33 usec
P1 10.00 usec
PLW1 20.93000031 W

F2 - Processing parameters
SI 65536
SF 500.1700012 MHz
WDW EM
SSB 0
LB 0.30 Hz
GB 0
PC 1.00

D8

1H_8scan CDCl3 {D:\Spectra} nmr 24

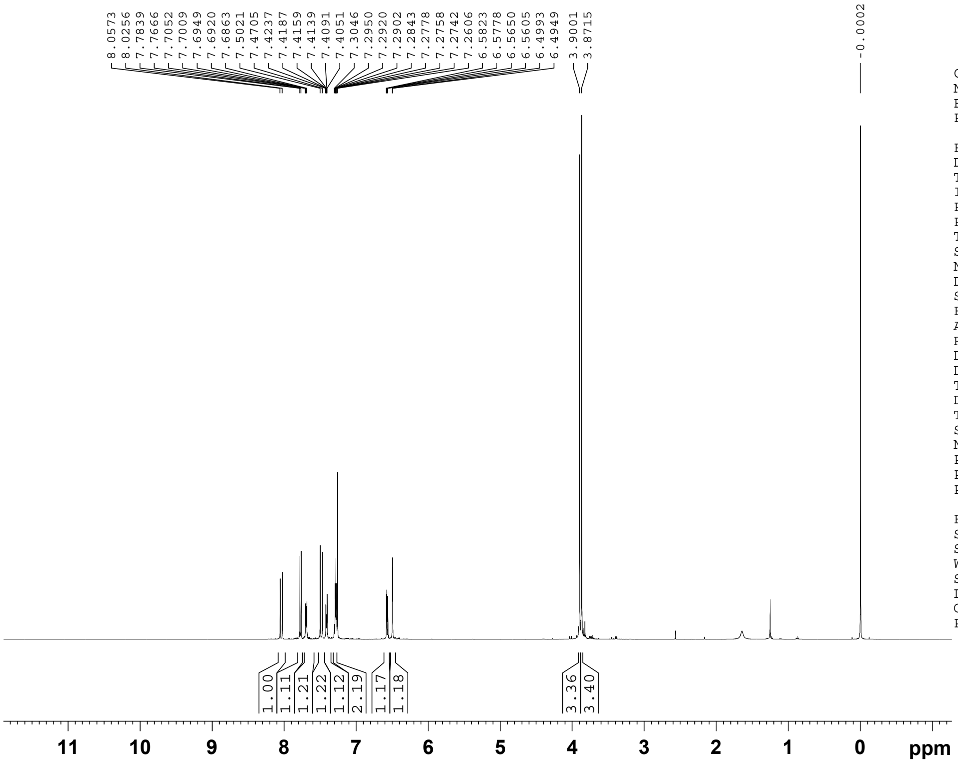

BRUKER
AVANCE NEO
500 MHz NMR
SPECTROMETER
SAIF, P.U.

Current Data Parameters
NAME Jun21-2022
EXPNO 240
PROCNO 1

F2 - Acquisition Parameters
Date_ 20220621
Time 15.47 h
INSTRUM Avance Neo 500
PROBHD Z119470_0333 (
PULPROG zg30
TD 65536
SOLVENT CDCl3
NS 16
DS 0
SWH 14705.883 Hz
FIDRES 0.448788 Hz
AQ 2.2282240 sec
RG 95.7854
DW 34.000 usec
DE 6.79 usec
TE 300.1 K
D1 1.00000000 sec
TD0 1
SFO1 500.1730885 MHz
NUC1 1H
P0 3.33 usec
P1 10.00 usec
PLW1 20.93000031 W

F2 - Processing parameters
SI 65536
SF 500.1700116 MHz
WDW EM
SSB 0
LB 0.30 Hz
GB 0
PC 1.00

D9
1H_8scan CDCl3 {D:\Spectra} nmr 25

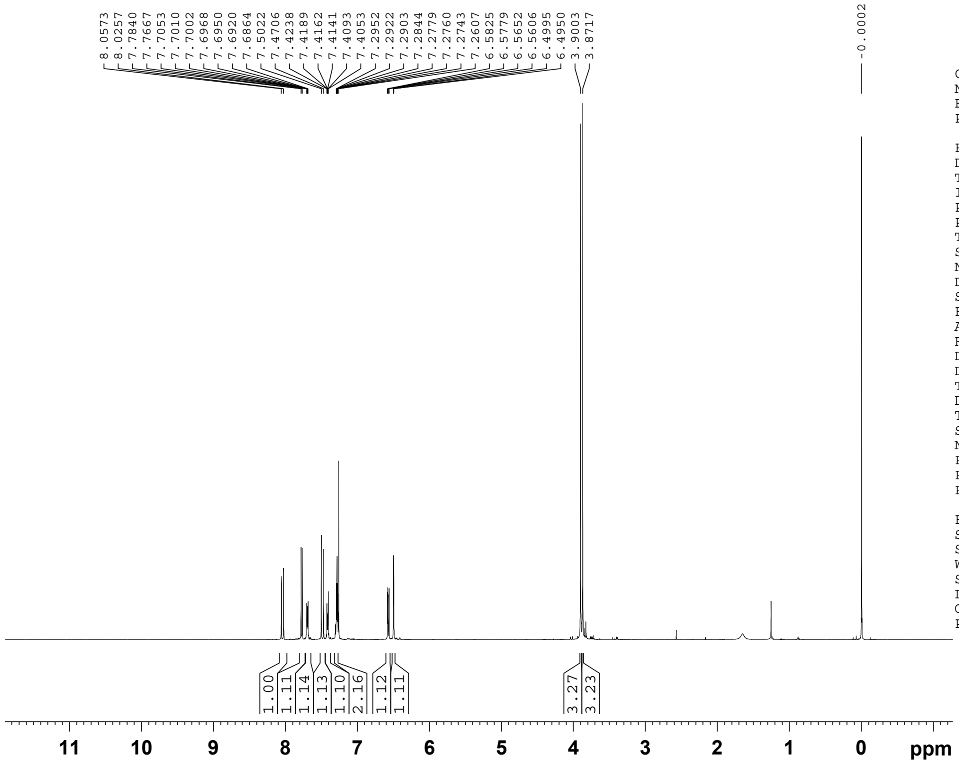

BRUKER
AVANCE NEO
500 MHz NMR
SPECTROMETER
SAIF, P.U.

Current Data Parameters

NAME	Jun21-2022
EXPNO	250
PROCNO	1

F2 - Acquisition Parameters

Date_	20220621
Time	16.15 h
INSTRUM	Avance Neo 500
PROBHD	Z119470_0333 (
PULPROG	zg30
TD	65536
SOLVENT	CDCl3
NS	16
DS	0
SWH	14705.883 Hz
FIDRES	0.448788 Hz
AQ	2.2282240 sec
RG	95.7854
DW	34.000 usec
DE	6.79 usec
TE	300.1 K
D1	1.00000000 sec
TD0	1
SFO1	500.1730885 MHz
NUC1	1H
P0	3.33 usec
P1	10.00 usec
PLW1	20.93000031 W

F2 - Processing parameters

SI	65536
SF	500.1700117 MHz
WDW	EM
SSB	0
LB	0.30 Hz
GB	0
PC	1.00

D10
1H_8scan CDCl3 {D:\Spectra} nmr 26

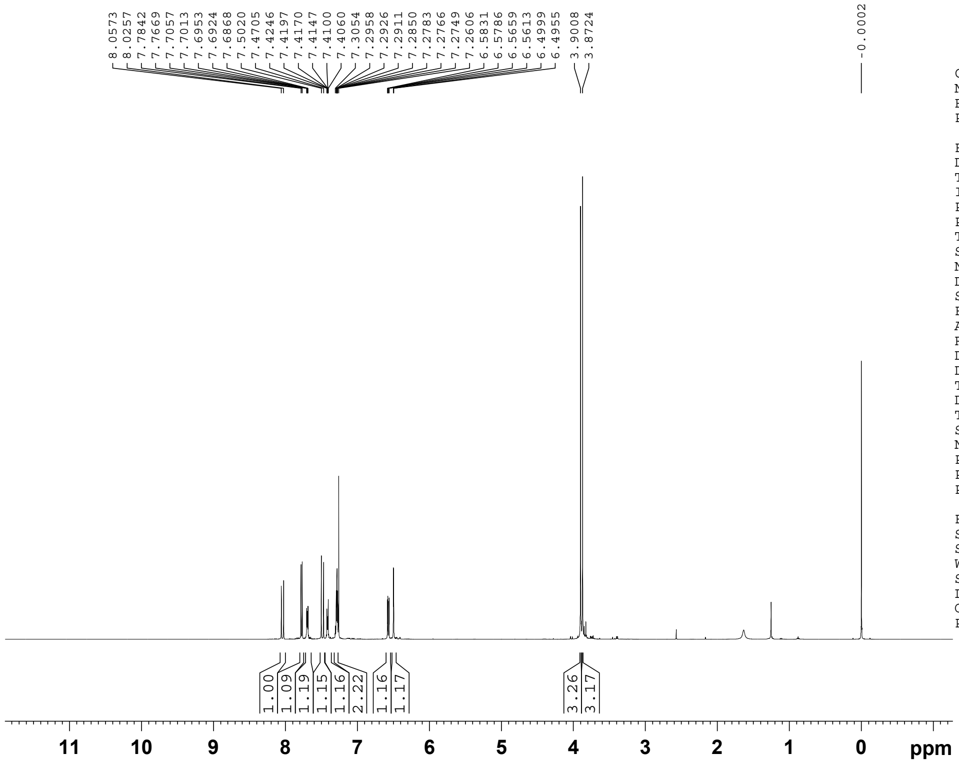

BRUKER
AVANCE NEO
500 MHz NMR
SPECTROMETER
SAIF, P.U.

Current Data Parameters

NAME	Jun21-2022
EXPNO	260
PROCNO	1

F2 - Acquisition Parameters

Date_	20220621
Time	16.31 h
INSTRUM	Avance Neo 500
PROBHD	Z119470_0333 (
PULPROG	zg30
TD	65536
SOLVENT	CDCl3
NS	16
DS	0
SWH	14705.883 Hz
FIDRES	0.448788 Hz
AQ	2.2282240 sec
RG	101
DW	34.000 usec
DE	6.79 usec
TE	300.1 K
D1	1.00000000 sec
TD0	1
SFO1	500.1730885 MHz
NUC1	1H
P0	3.33 usec
P1	10.00 usec
PLW1	20.93000031 W

F2 - Processing parameters

SI	65536
SF	500.1700117 MHz
WDW	EM
SSB	0
LB	0.30 Hz
GB	0
PC	1.00

D11
1H_8scan CDCl3 {D:\Spectra} nmr 27

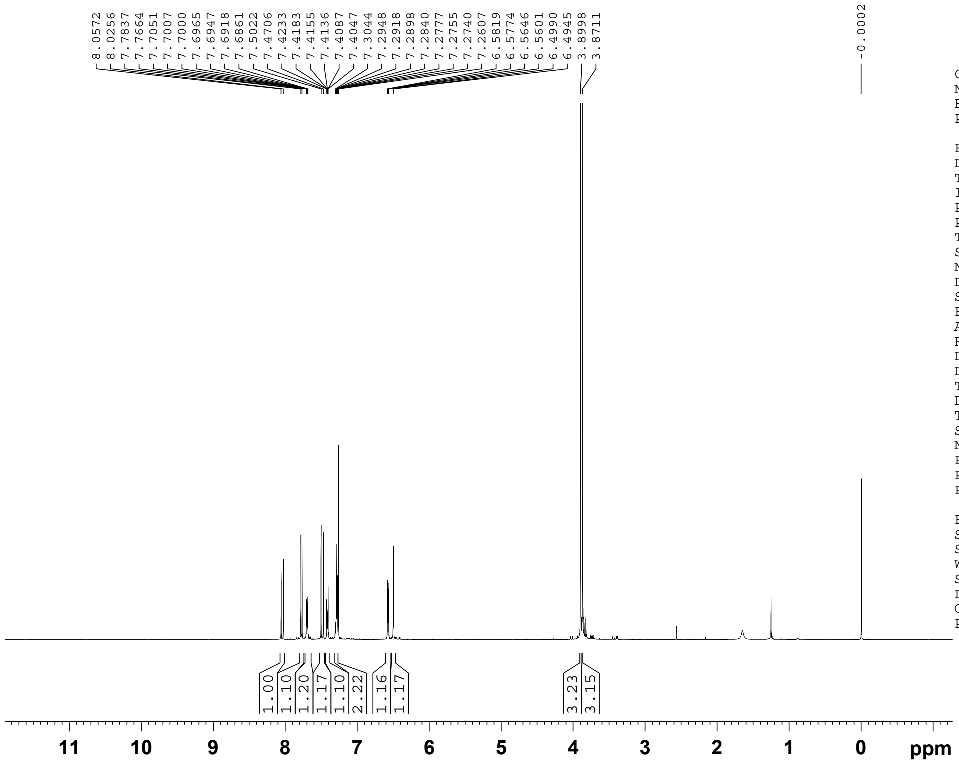

BRUKER
AVANCE NEO
500 MHz NMR
SPECTROMETER
SAIF, P.U.

Current Data Parameters
NAME Jun21-2022
EXPNO 270
PROCNO 1

F2 - Acquisition Parameters
Date_ 20220621
Time 16.47 h
INSTRUM Avance Neo 500
PROBHD Z119470_0333 (
PULPROG zg30
TD 65536
SOLVENT CDCl3
NS 16
DS 0
SWH 14705.883 Hz
FIDRES 0.448788 Hz
AQ 2.2282240 sec
RG 95.7854
DW 34.000 usec
DE 6.79 usec
TE 300.1 K
D1 1.00000000 sec
TD0 1
SFO1 500.1730885 MHz
NUC1 1H
P0 3.33 usec
P1 10.00 usec
PLW1 20.93000031 W

F2 - Processing parameters
SI 65536
SF 500.1700116 MHz
WDW EM
SSB 0
LB 0.30 Hz
GB 0
PC 1.00

D12
1H_8scan CDCl3 {D:\Spectra} nmr 28

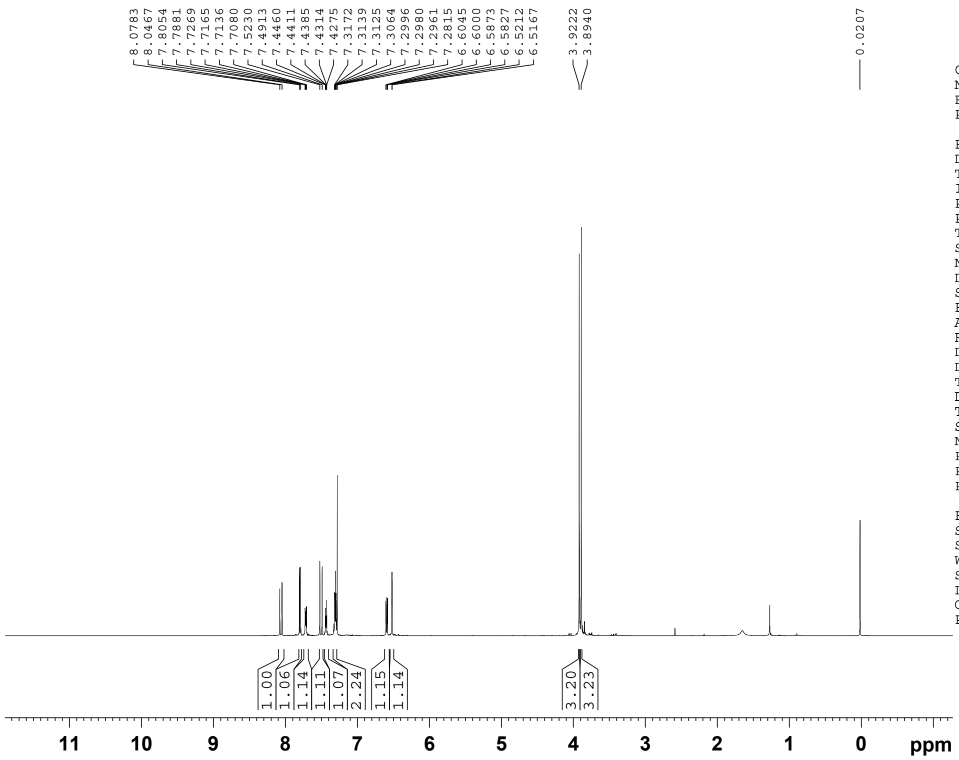

BRUKER
AVANCE NEO
500 MHz NMR
SPECTROMETER
SAIF, P.U.

Current Data Parameters
NAME Jun21-2022
EXPNO 280
PROCNO 1

F2 - Acquisition Parameters
Date_ 20220621
Time 17.01 h
INSTRUM Avance Neo 500
PROBHD Z119470_0333 (
PULPROG zg30
TD 65536
SOLVENT CDCl3
NS 16
DS 0
SWH 14705.883 Hz
FIDRES 0.448788 Hz
AQ 2.2282240 sec
RG 101
DW 34.000 usec
DE 6.79 usec
TE 300.1 K
D1 1.00000000 sec
TD0 1
SFO1 500.1730885 MHz
NUC1 1H
P0 3.33 usec
P1 10.00 usec
PLW1 20.93000031 W

F2 - Processing parameters
SI 65536
SF 500.1700012 MHz
WDW EM
SSB 0
LB 0.30 Hz
GB 0
PC 1.00

D13
1H_8scan CDCl3 {D:\Spectra} nmr 29

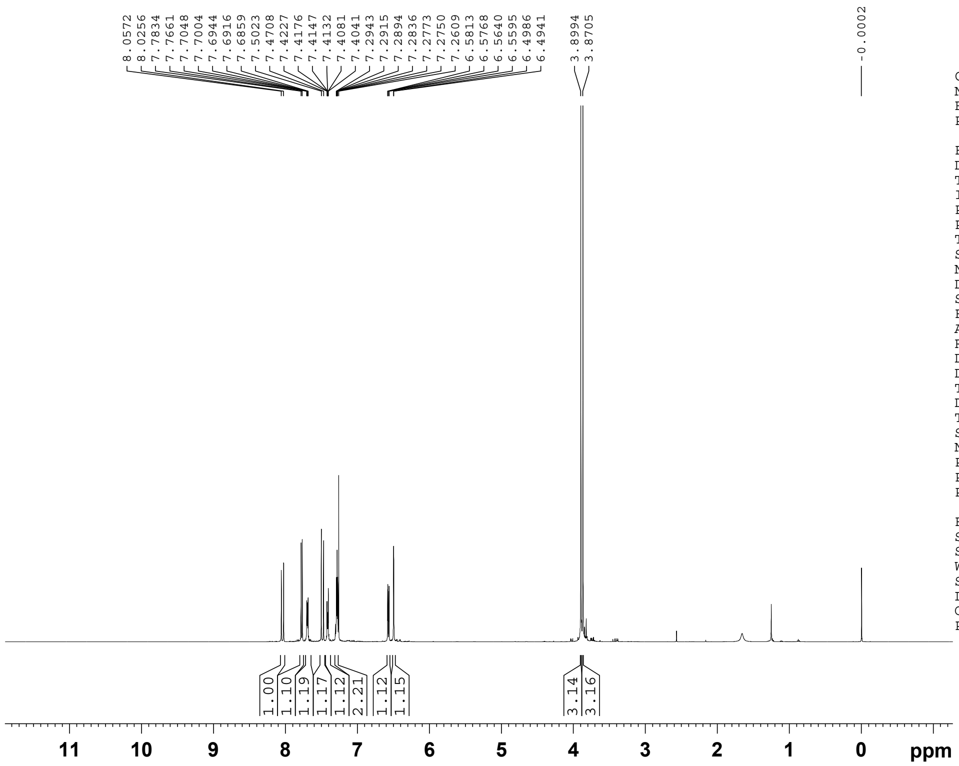


```
BRUKER
AVANCE NEO
500 MHz NMR
SPECTROMETER
SAIF, P.U.

Current Data Parameters
NAME        Jun21-2022
EXPNO              290
PROCNO               1

F2 - Acquisition Parameters
Date_         20220621
Time             17.15 h
INSTRUM  Avance Neo 500
PROBHD   Z119470_0333 (
PULPROG           zg30
TD               65536
SOLVENT          CDCl3
NS                  16
DS                   0
SWH          14705.883 Hz
FIDRES        0.448788 Hz
AQ           2.2282240 sec
RG             95.7854
DW              34.000 usec
DE                6.79 usec
TE               300.1 K
D1          1.00000000 sec
TD0                  1
SFO1       500.1730885 MHz
NUC1                1H
P0                3.33 usec
P1               10.00 usec
PLW1       20.93000031 W

F2 - Processing parameters
SI               65536
SF         500.1700115 MHz
WDW                 EM
SSB                  0
LB                0.30 Hz
GB                   0
PC                1.00
```

D14
1H_8scan CDCl3 {D:\Spectra} nmr 30

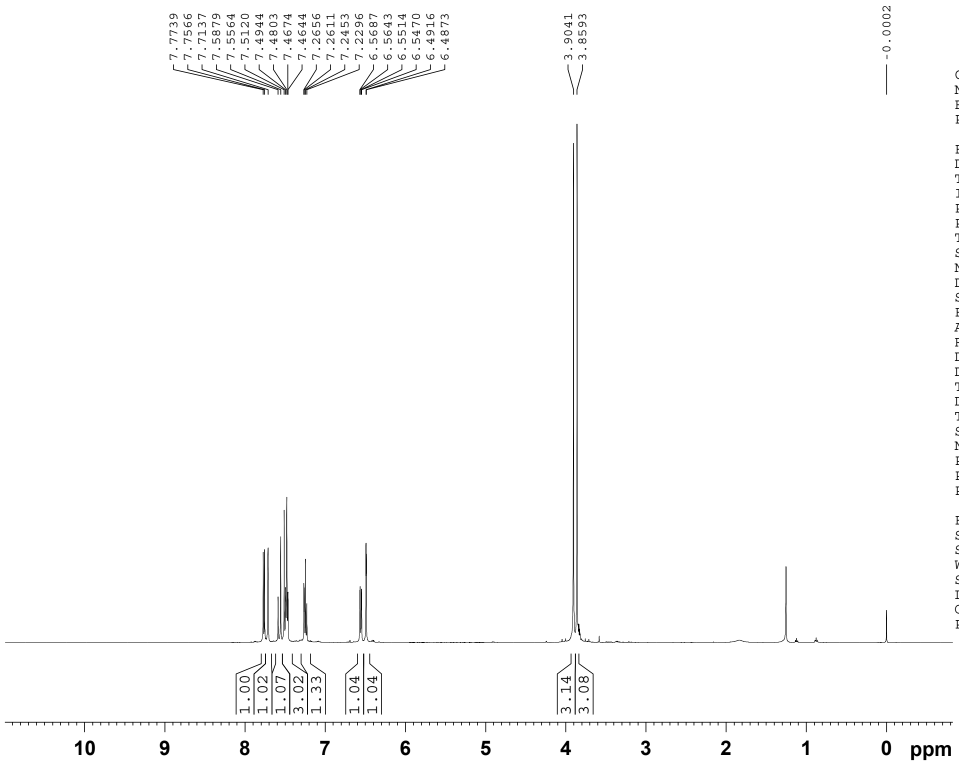


```
BRUKER
AVANCE NEO
500 MHz NMR
SPECTROMETER
SAIF, P.U.

Current Data Parameters
NAME        Jun21-2022
EXPNO              300
PROCNO               1

F2 - Acquisition Parameters
Date_         20220621
Time             17.26 h
INSTRUM  Avance Neo 500
PROBHD   Z119470_0333 (
PULPROG           zg30
TD               65536
SOLVENT          CDCl3
NS                  16
DS                   0
SWH          14705.883 Hz
FIDRES        0.448788 Hz
AQ           2.2282240 sec
RG              59.445
DW              34.000 usec
DE                6.79 usec
TE               300.1 K
D1          1.00000000 sec
TD0                  1
SFO1       500.1730885 MHz
NUC1                1H
P0                3.33 usec
P1               10.00 usec
PLW1       20.93000031 W

F2 - Processing parameters
SI               65536
SF         500.1700090 MHz
WDW                 EM
SSB                  0
LB                0.30 Hz
GB                   0
PC                1.00
```

D15
1H_8scan CDCl3 {D:\Spectra} nmr 31

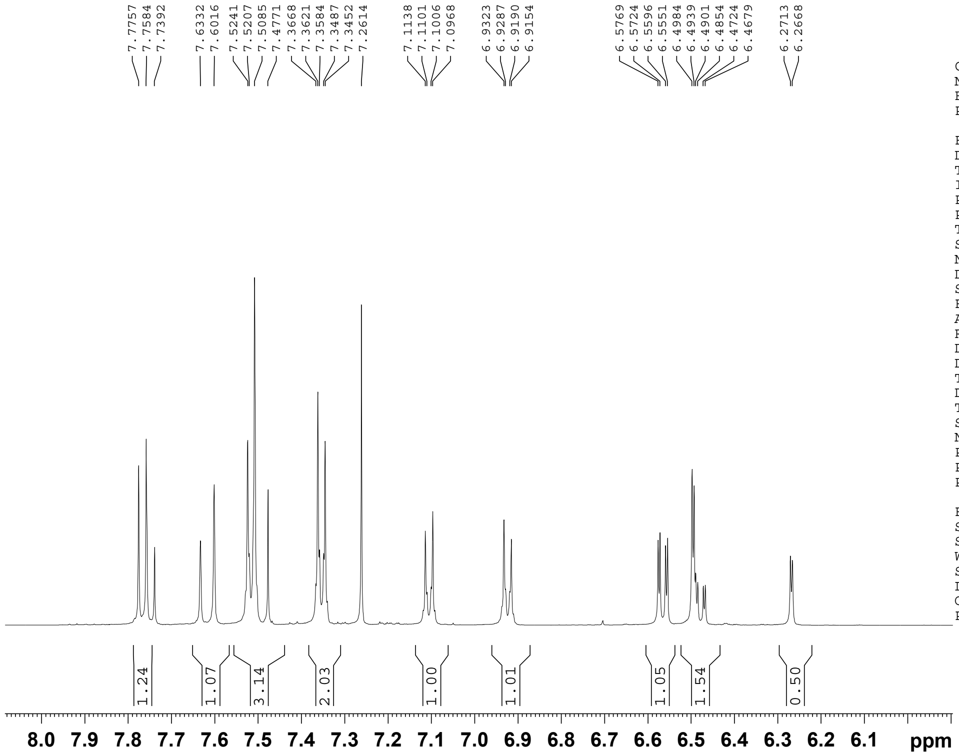

BRUKER
AVANCE NEO
500 MHz NMR
SPECTROMETER
SAIF, P.U.

Current Data Parameters	
NAME	Jun21-2022
EXPNO	310
PROCNO	1

F2 - Acquisition Parameters	
Date_	20220621
Time	17.54 h
INSTRUM	Avance Neo 500
PROBHD	Z119470_0333 (
PULPROG	zg30
TD	65536
SOLVENT	CDCl3
NS	16
DS	0
SWH	14705.883 Hz
FIDRES	0.448788 Hz
AQ	2.2282240 sec
RG	95.7854
DW	34.000 usec
DE	6.79 usec
TE	300.1 K
D1	1.00000000 sec
TD0	1
SFO1	500.1730885 MHz
NUC1	1H
P0	3.33 usec
P1	10.00 usec
PLW1	20.93000031 W

F2 - Processing parameters	
SI	65536
SF	500.1700112 MHz
WDW	EM
SSB	0
LB	0.30 Hz
GB	0
PC	1.00

D16
1H_8scan CDCl3 {D:\Spectra} nmr 32

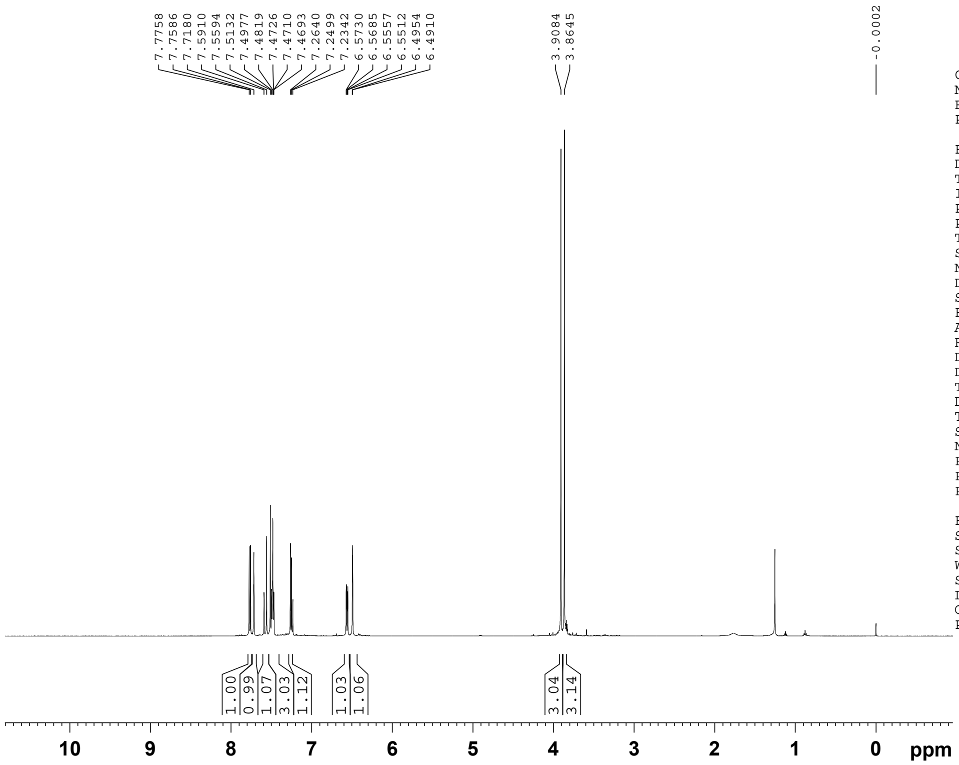

BRUKER
AVANCE NEO
500 MHz NMR
SPECTROMETER
SAIF, P.U.

Current Data Parameters	
NAME	Jun21-2022
EXPNO	320
PROCNO	1

F2 - Acquisition Parameters	
Date_	20220622
Time	10.42 h
INSTRUM	Avance Neo 500
PROBHD	Z119470_0333 (
PULPROG	zg30
TD	65536
SOLVENT	CDCl3
NS	16
DS	0
SWH	14705.883 Hz
FIDRES	0.448788 Hz
AQ	2.2282240 sec
RG	70.3334
DW	34.000 usec
DE	6.79 usec
TE	300.2 K
D1	1.00000000 sec
TD0	1
SFO1	500.1730885 MHz
NUC1	1H
P0	3.33 usec
P1	10.00 usec
PLW1	20.93000031 W

F2 - Processing parameters	
SI	65536
SF	500.1700100 MHz
WDW	EM
SSB	0
LB	0.30 Hz
GB	0
PC	1.00

D17
1H_8scan CDCl3 {D:\Spectra} nmr 33

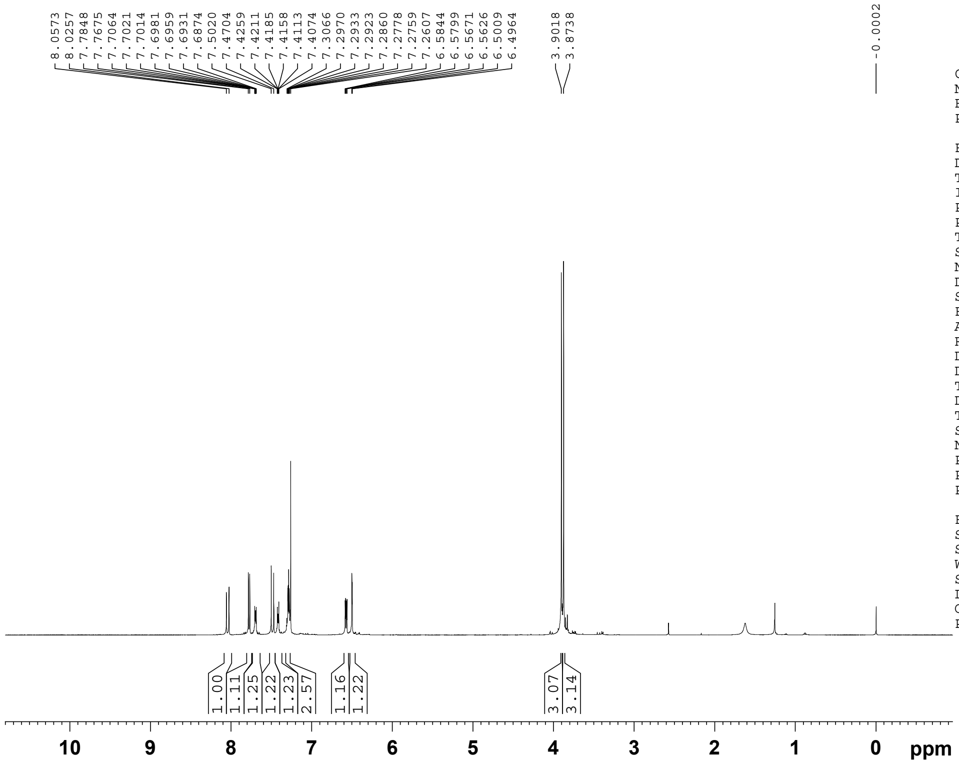

BRUKER
AVANCE NEO
500 MHz NMR
SPECTROMETER
SAIF, P.U.

```
Current Data Parameters
NAME          Jun21-2022
EXPNO                330
PROCNO                 1

F2 - Acquisition Parameters
Date_           20220622
Time               10.54 h
INSTRUM   Avance Neo 500
PROBHD   Z119470_0333 (
PULPROG             zg30
TD                 65536
SOLVENT            CDCl3
NS                    16
DS                     0
SWH            14705.883 Hz
FIDRES          0.448788 Hz
AQ             2.2282240 sec
RG                   101
DW                34.000 usec
DE                  6.79 usec
TE                 300.1 K
D1            1.00000000 sec
TD0                    1
SFO1         500.1730885 MHz
NUC1                  1H
P0                  3.33 usec
P1                 10.00 usec
PLW1         20.93000031 W

F2 - Processing parameters
SI                 65536
SF           500.1700115 MHz
WDW                   EM
SSB                    0
LB                  0.30 Hz
GB                     0
PC                  1.00
```

D18
1H_8scan CDCl3 {D:\Spectra} nmr 34

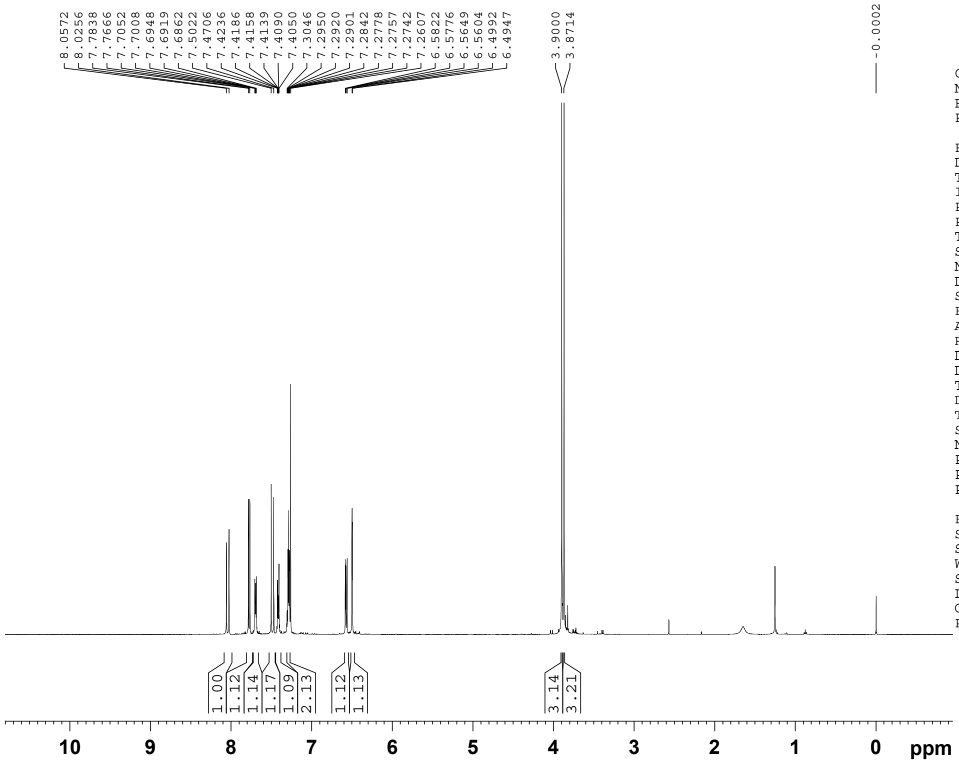

BRUKER
AVANCE NEO
500 MHz NMR
SPECTROMETER
SAIF, P.U.

```
Current Data Parameters
NAME          Jun21-2022
EXPNO                340
PROCNO                 1

F2 - Acquisition Parameters
Date_           20220622
Time               11.50 h
INSTRUM   Avance Neo 500
PROBHD   Z119470_0333 (
PULPROG             zg30
TD                 65536
SOLVENT            CDCl3
NS                    16
DS                     0
SWH            14705.883 Hz
FIDRES          0.448788 Hz
AQ             2.2282240 sec
RG               95.7854
DW                34.000 usec
DE                  6.79 usec
TE                 300.2 K
D1            1.00000000 sec
TD0                    1
SFO1         500.1730885 MHz
NUC1                  1H
P0                  3.33 usec
P1                 10.00 usec
PLW1         20.93000031 W

F2 - Processing parameters
SI                 65536
SF           500.1700116 MHz
WDW                   EM
SSB                    0
LB                  0.30 Hz
GB                     0
PC                  1.00
```

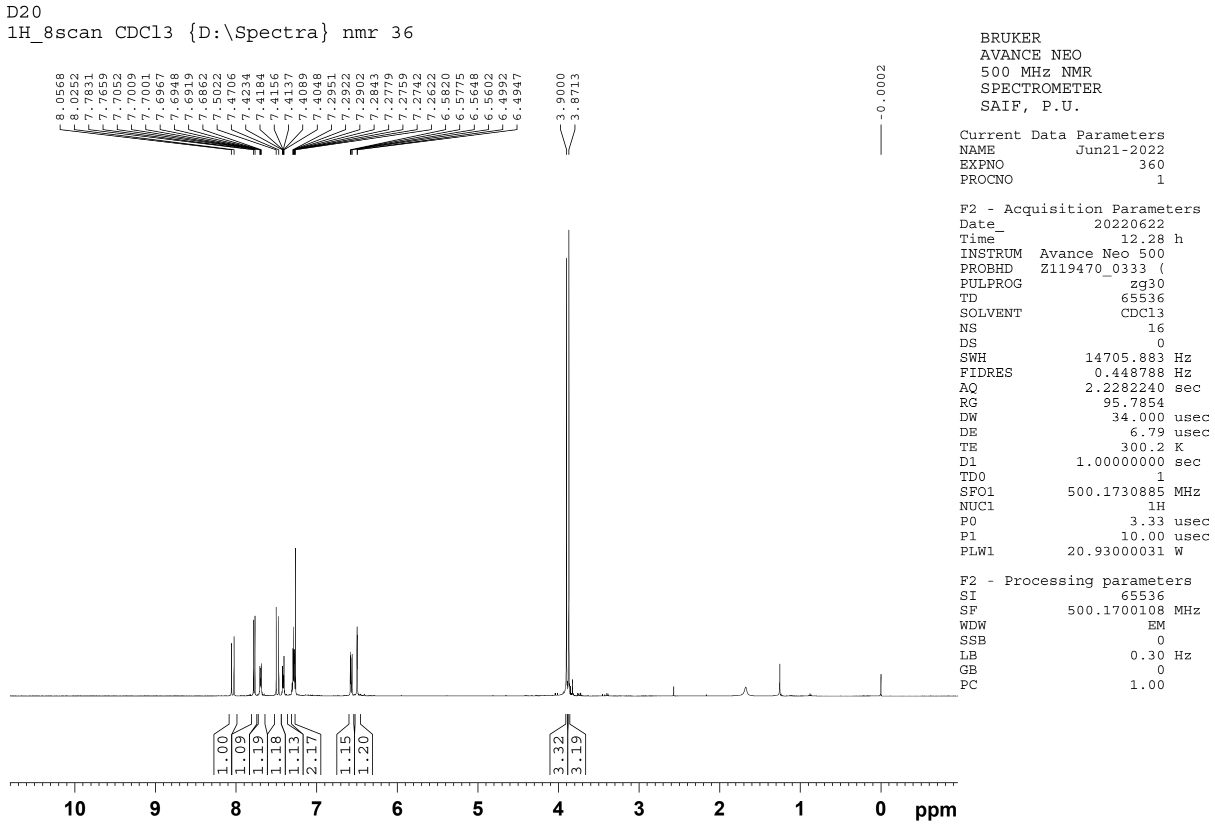
D20
1H_8scan CDCl3 {D:\Spectra} nmr 36
8.0568
8.0252
7.7831
7.7659
7.7052
7.7009
7.7001
7.6967
7.6948
7.6919
7.6862
7.5022
7.4706
7.4234
7.4184
7.4156
7.4137
7.4089
7.4048
7.2951
7.2922
7.2902
7.2843
7.2779
7.2759
7.2742
7.2622
6.5820
6.5775
6.5648
6.5602
6.4992
6.4947
3.9000
3.8713
-0.0002
1.00
1.09
1.19
1.18
1.13
2.17
1.15
1.20
3.32
3.19
10
9
8
7
6
5
4
3
2
1
0
ppm
BRUKER
AVANCE NEO
500 MHz NMR
SPECTROMETER
SAIF, P.U.
Current Data Parameters
NAME Jun21-2022
EXPNO 360
PROCNO 1
F2 - Acquisition Parameters
Date_ 20220622
Time 12.28 h
INSTRUM Avance Neo 500
PROBHD Z119470_0333 (
PULPROG zg30
TD 65536
SOLVENT CDCl3
NS 16
DS 0
SWH 14705.883 Hz
FIDRES 0.448788 Hz
AQ 2.2282240 sec
RG 95.7854
DW 34.000 usec
DE 6.79 usec
TE 300.2 K
D1 1.00000000 sec
TD0 1
SFO1 500.1730885 MHz
NUC1 1H
P0 3.33 usec
P1 10.00 usec
PLW1 20.93000031 W
F2 - Processing parameters
SI 65536
SF 500.1700108 MHz
WDW EM
SSB 0
LB 0.30 Hz
GB 0
PC 1.00

Figure S2. ^{13}C-NMR spectra

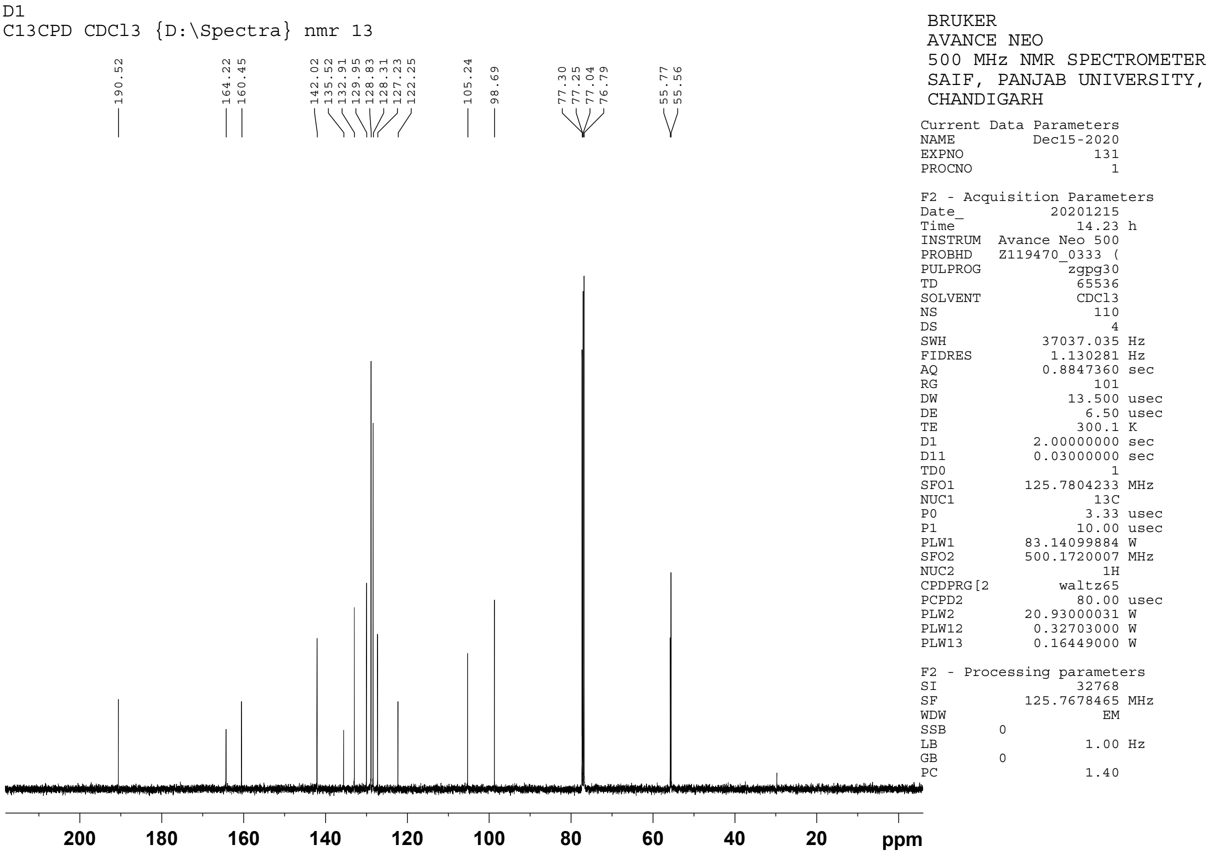

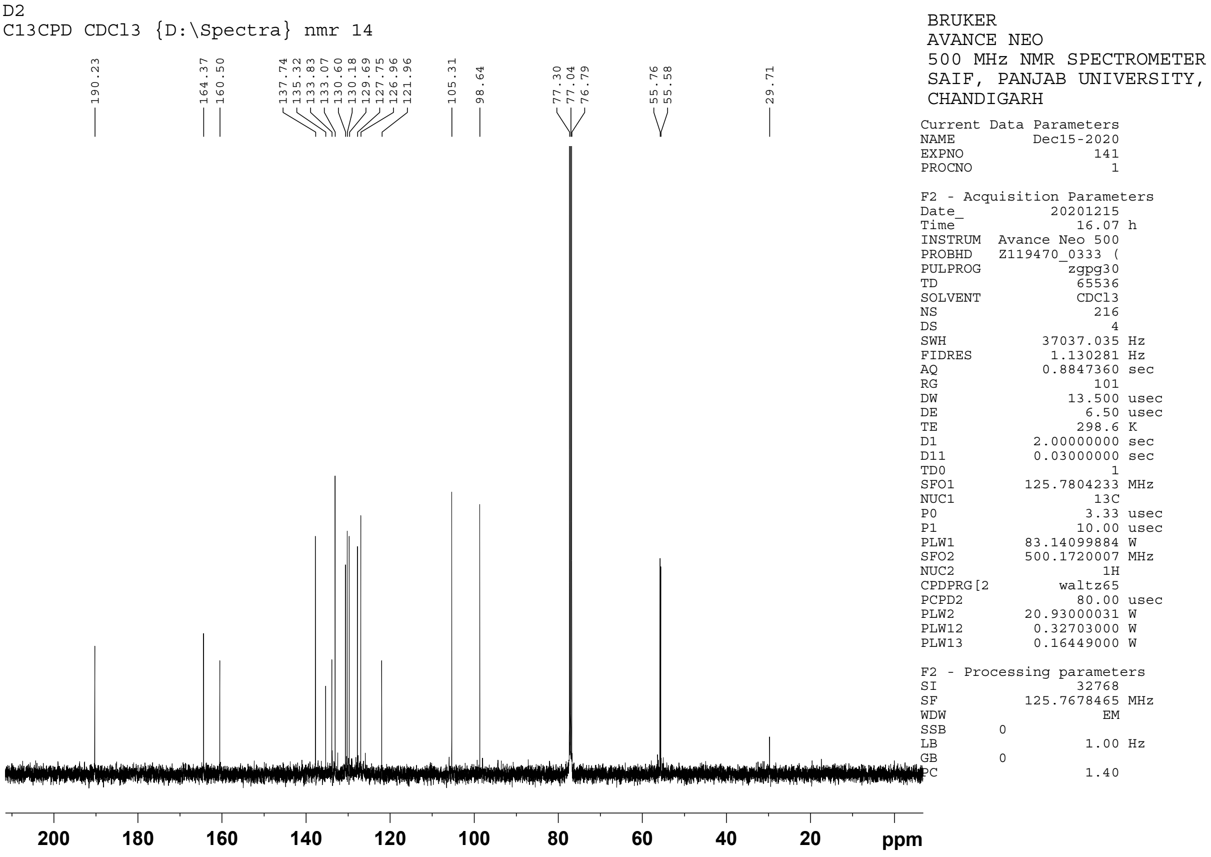

D3
C13CPD CDCl3 {D:\Spectra} nmr 15

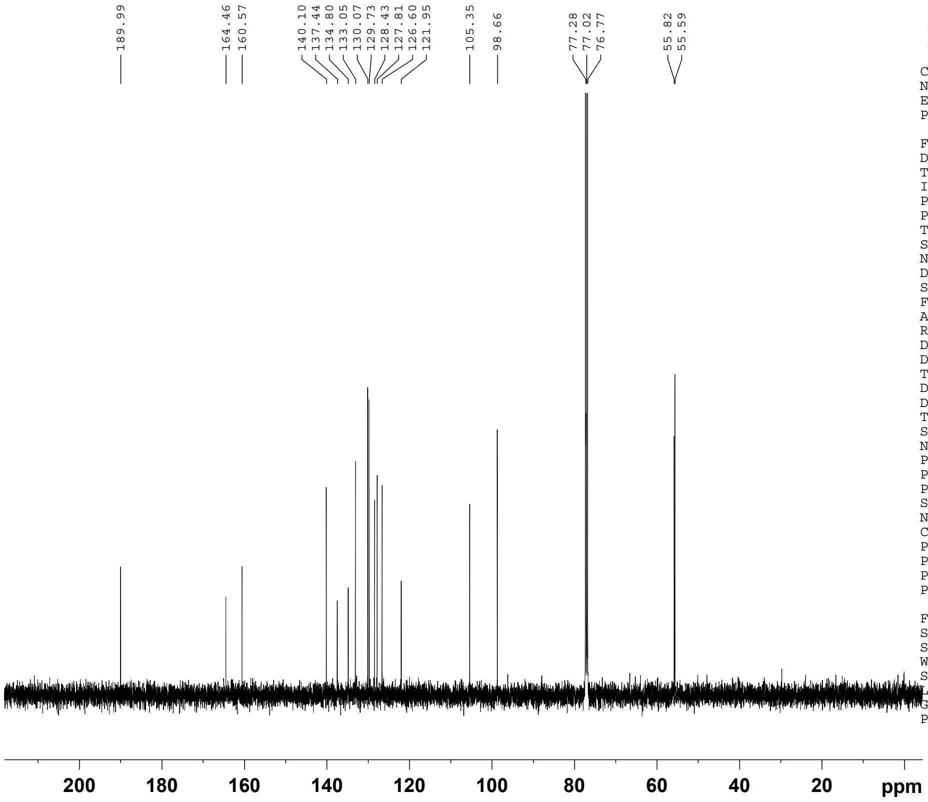

BRUKER
AVANCE NEO
500 MHz NMR SPECTROMETER
SAIF, PANJAB UNIVERSITY,
CHANDIGARH

```
Current Data Parameters
NAME          Dec15-2020
EXPNO                152
PROCNO                 1

F2 - Acquisition Parameters
Date_           20201215
Time               17.14 h
INSTRUM  Avance Neo 500
PROBHD   Z119470_0333 (
PULPROG           zgpg30
TD                 65536
SOLVENT            CDCl3
NS                   240
DS                     4
SWH            37037.035 Hz
FIDRES          1.130281 Hz
AQ             0.8847360 sec
RG                   101
DW                13.500 usec
DE                  6.50 usec
TE                 298.8 K
D1            2.00000000 sec
D11           0.03000000 sec
TD0                    1
SFO1        125.7804233 MHz
NUC1                 13C
P0                  3.33 usec
P1                 10.00 usec
PLW1         83.14099884 W
SFO2        500.1720007 MHz
NUC2                  1H
CPDPRG[2          waltz65
PCPD2              80.00 usec
PLW2         20.93000031 W
PLW12         0.32703000 W
PLW13         0.16449000 W

F2 - Processing parameters
SI                 32768
SF          125.7678465 MHz
WDW                   EM
SSB      0
LB                  1.00 Hz
GB       0
PC                  1.40
```

D4
C13CPD CDCl3 {D:\Spectra} nmr 16

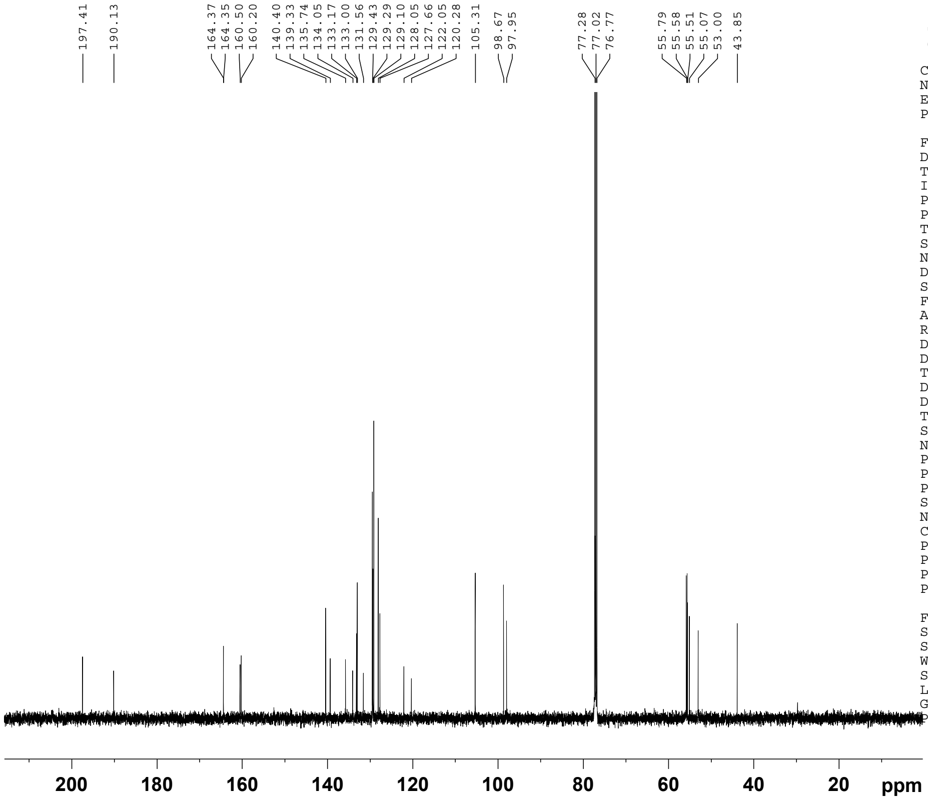

BRUKER
AVANCE NEO
500 MHz NMR SPECTROMETER
SAIF, PANJAB UNIVERSITY,
CHANDIGARH

```
Current Data Parameters
NAME          Dec15-2020
EXPNO                161
PROCNO                 1

F2 - Acquisition Parameters
Date_           20201215
Time               17.45 h
INSTRUM  Avance Neo 500
PROBHD   Z119470_0333 (
PULPROG           zgpg30
TD                 65536
SOLVENT            CDCl3
NS                   512
DS                     4
SWH            37037.035 Hz
FIDRES          1.130281 Hz
AQ             0.8847360 sec
RG                   101
DW                13.500 usec
DE                  6.50 usec
TE                 298.6 K
D1            2.00000000 sec
D11           0.03000000 sec
TD0                    1
SFO1        125.7804233 MHz
NUC1                 13C
P0                  3.33 usec
P1                 10.00 usec
PLW1         83.14099884 W
SFO2        500.1720007 MHz
NUC2                  1H
CPDPRG[2          waltz65
PCPD2              80.00 usec
PLW2         20.93000031 W
PLW12         0.32703000 W
PLW13         0.16449000 W

F2 - Processing parameters
SI                 32768
SF          125.7678465 MHz
WDW                   EM
SSB      0
LB                  1.00 Hz
GB       0
PC                  1.40
```

D5
C13CPD CDCl3 {D:\Spectra} nmr 17

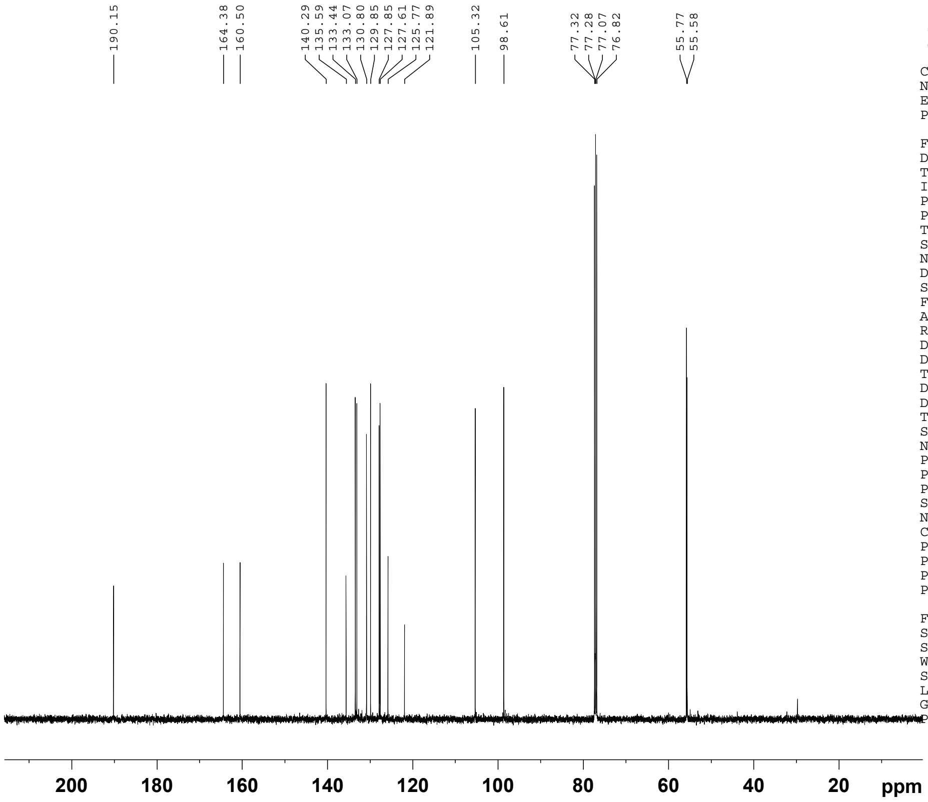

BRUKER
AVANCE NEO
500 MHz NMR SPECTROMETER
SAIF, PANJAB UNIVERSITY,
CHANDIGARH

```
Current Data Parameters
NAME          Dec15-2020
EXPNO                171
PROCNO                 1

F2 - Acquisition Parameters
Date_           20201216
Time                9.53 h
INSTRUM  Avance Neo 500
PROBHD   Z119470_0333 (
PULPROG           zgpg30
TD                 65536
SOLVENT            CDCl3
NS                   170
DS                     4
SWH            37037.035 Hz
FIDRES          1.130281 Hz
AQ             0.8847360 sec
RG                   101
DW                13.500 usec
DE                  6.50 usec
TE                 297.8 K
D1            2.00000000 sec
D11           0.03000000 sec
TD0                    1
SFO1         125.7804233 MHz
NUC1                 13C
P0                  3.33 usec
P1                 10.00 usec
PLW1         83.14099884 W
SFO2         500.1720007 MHz
NUC2                  1H
CPDPRG[2         waltz65
PCPD2              80.00 usec
PLW2         20.93000031 W
PLW12         0.32703000 W
PLW13         0.16449000 W

F2 - Processing parameters
SI                 32768
SF           125.7678465 MHz
WDW                   EM
SSB      0
LB                  1.00 Hz
GB       0
PC                  1.40
```

D6
C13CPD CDCl3 {D:\Spectra} nmr 18

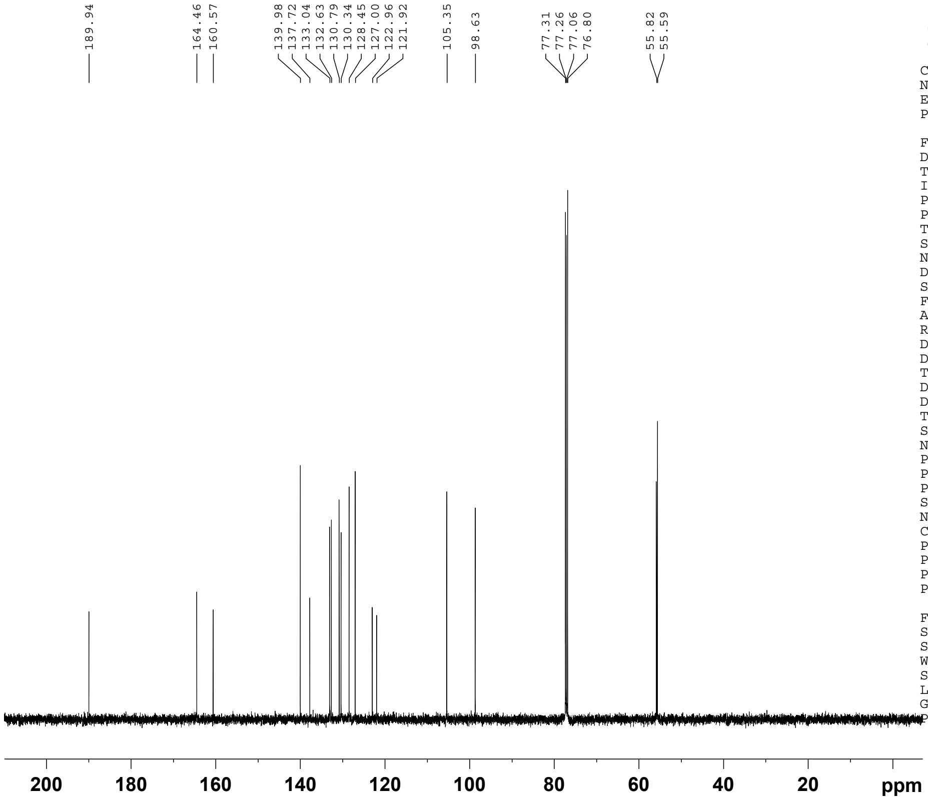

BRUKER
AVANCE NEO
500 MHz NMR SPECTROMETER
SAIF, PANJAB UNIVERSITY,
CHANDIGARH

```
Current Data Parameters
NAME          Dec15-2020
EXPNO                181
PROCNO                 1

F2 - Acquisition Parameters
Date_           20201216
Time               10.00 h
INSTRUM  Avance Neo 500
PROBHD   Z119470_0333 (
PULPROG           zgpg30
TD                 65536
SOLVENT            CDCl3
NS                    84
DS                     4
SWH            37037.035 Hz
FIDRES          1.130281 Hz
AQ             0.8847360 sec
RG                   101
DW                13.500 usec
DE                  6.50 usec
TE                 297.8 K
D1            2.00000000 sec
D11           0.03000000 sec
TD0                    1
SFO1         125.7804233 MHz
NUC1                 13C
P0                  3.33 usec
P1                 10.00 usec
PLW1         83.14099884 W
SFO2         500.1720007 MHz
NUC2                  1H
CPDPRG[2         waltz65
PCPD2              80.00 usec
PLW2         20.93000031 W
PLW12         0.32703000 W
PLW13         0.16449000 W

F2 - Processing parameters
SI                 32768
SF           125.7678465 MHz
WDW                   EM
SSB      0
LB                  1.00 Hz
GB       0
PC                  1.40
```

D7
C13CPD CDCl3 {D:\Spectra} nmr 19

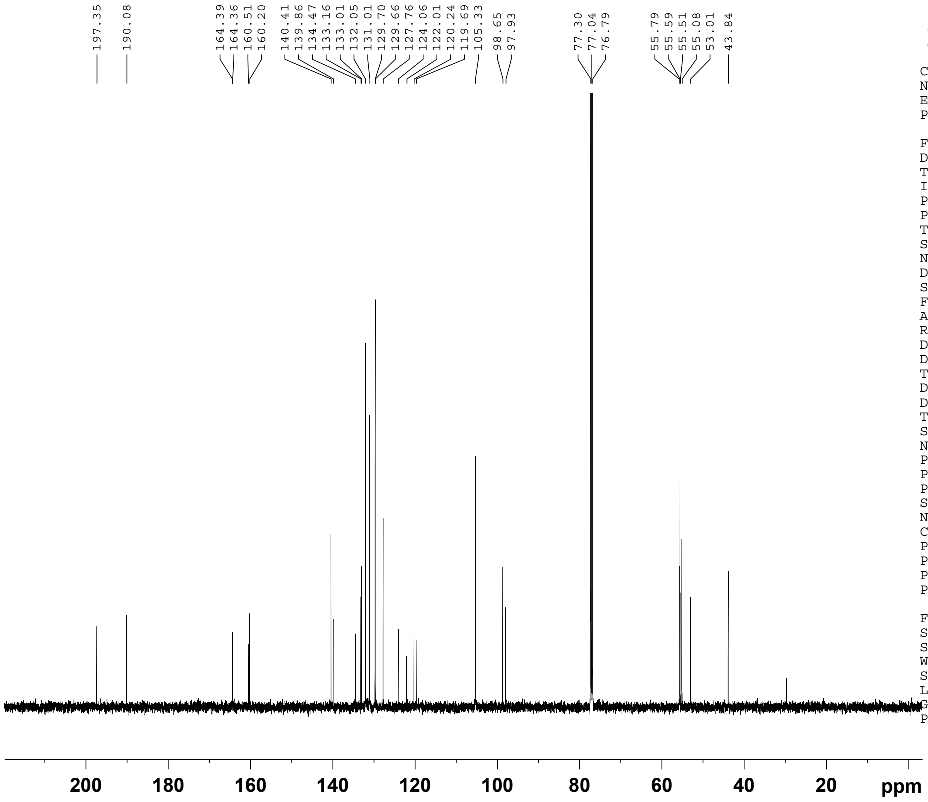

BRUKER
AVANCE NEO
500 MHz NMR SPECTROMETER
SAIF, PANJAB UNIVERSITY,
CHANDIGARH

Current Data Parameters
NAME Dec15-2020
EXPNO 191
PROCNO 1

F2 - Acquisition Parameters
Date_ 20201216
Time 10.13 h
INSTRUM Avance Neo 500
PROBHD Z119470_0333 (
PULPROG zgpg30
TD 65536
SOLVENT CDCl3
NS 202
DS 4
SWH 37037.035 Hz
FIDRES 1.130281 Hz
AQ 0.8847360 sec
RG 101
DW 13.500 usec
DE 6.50 usec
TE 297.7 K
D1 2.00000000 sec
D11 0.03000000 sec
TD0 1
SFO1 125.7804233 MHz
NUC1 13C
P0 3.33 usec
P1 10.00 usec
PLW1 83.14099884 W
SFO2 500.1720007 MHz
NUC2 1H
CPDPRG[2 waltz65
PCPD2 80.00 usec
PLW2 20.93000031 W
PLW12 0.32703000 W
PLW13 0.16449000 W

F2 - Processing parameters
SI 32768
SF 125.7678465 MHz
WDW EM
SSB 0
LB 1.00 Hz
GB 0
PC 1.40

D8
C13CPD CDCl3 {D:\Spectra} nmr 24

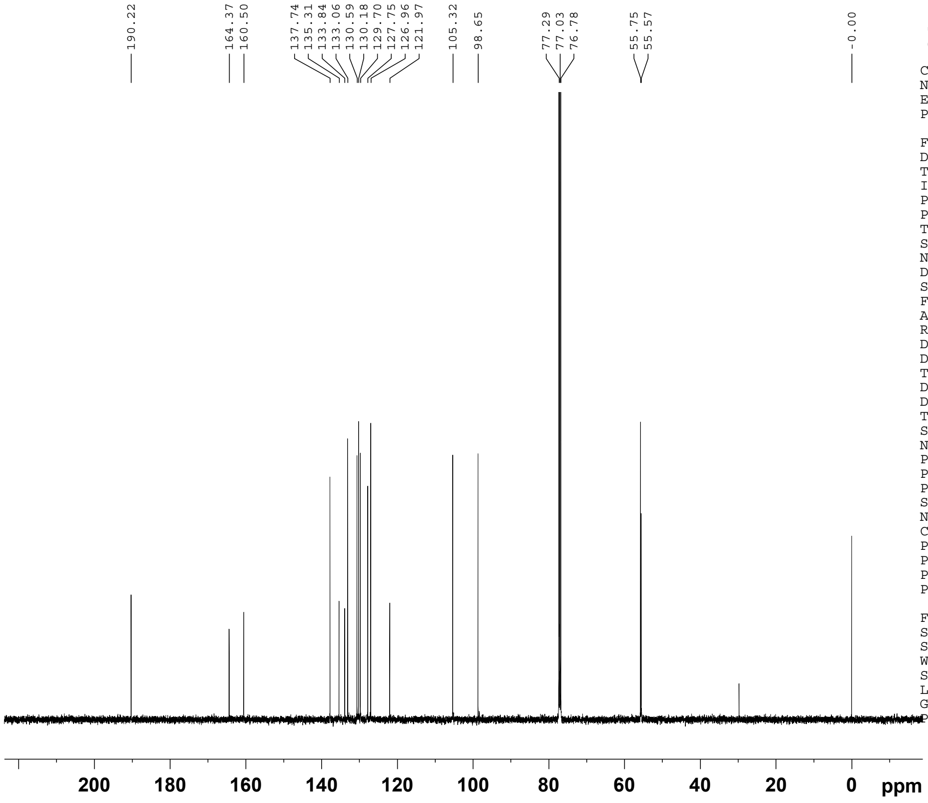

BRUKER
AVANCE NEO
500 MHz NMR SPECTROMETER
SAIF, PANJAB UNIVERSITY,
CHANDIGARH

Current Data Parameters
NAME Jun21-2022
EXPNO 241
PROCNO 1

F2 - Acquisition Parameters
Date_ 20220621
Time 16.13 h
INSTRUM Avance Neo 500
PROBHD Z119470_0333 (
PULPROG zgpg30
TD 65536
SOLVENT CDCl3
NS 512
DS 4
SWH 37037.035 Hz
FIDRES 1.130281 Hz
AQ 0.8847360 sec
RG 101
DW 13.500 usec
DE 6.50 usec
TE 300.6 K
D1 2.00000000 sec
D11 0.03000000 sec
TD0 1
SFO1 125.7804233 MHz
NUC1 13C
P0 3.33 usec
P1 10.00 usec
PLW1 83.14099884 W
SFO2 500.1720007 MHz
NUC2 1H
CPDPRG[2 waltz65
PCPD2 80.00 usec
PLW2 20.93000031 W
PLW12 0.32703000 W
PLW13 0.16449000 W

F2 - Processing parameters
SI 32768
SF 125.7678466 MHz
WDW EM
SSB 0
LB 1.00 Hz
GB 0
PC 1.40

D9
C13CPD CDCl3 {D:\Spectra} nmr 25

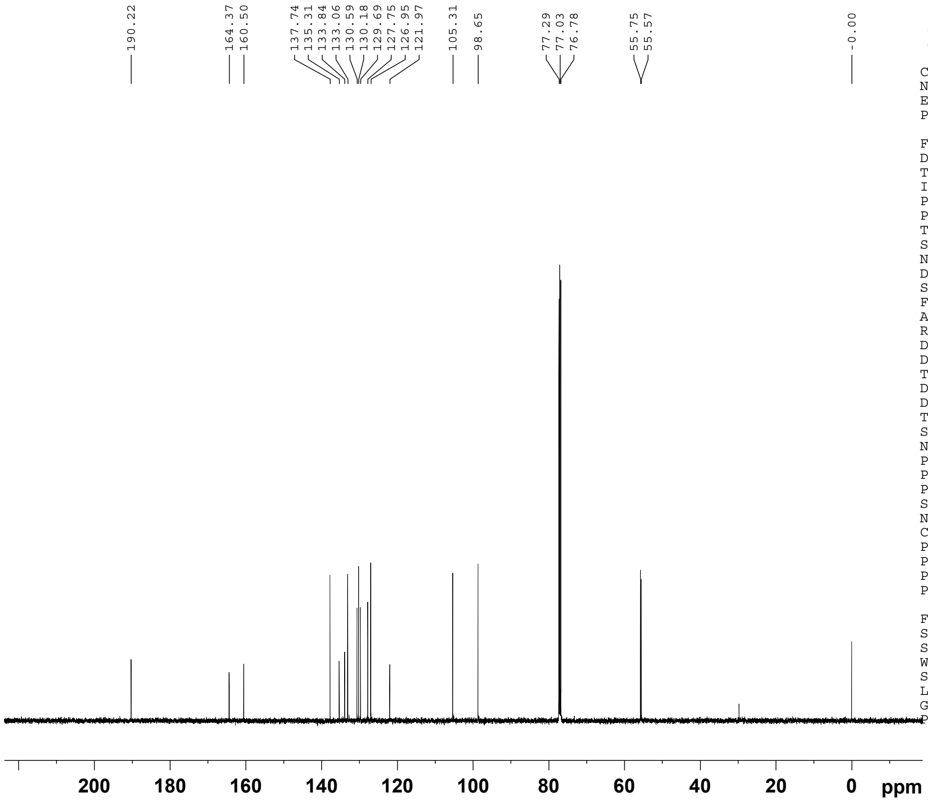

BRUKER
AVANCE NEO
500 MHz NMR SPECTROMETER
SAIF, PANJAB UNIVERSITY,
CHANDIGARH

Current Data Parameters
NAME Jun21-2022
EXPNO 251
PROCNO 1

F2 - Acquisition Parameters
Date_ 20220621
Time 16.29 h
INSTRUM Avance Neo 500
PROBHD Z119470_0333 (
PULPROG zgpg30
TD 65536
SOLVENT CDCl3
NS 265
DS 4
SWH 37037.035 Hz
FIDRES 1.130281 Hz
AQ 0.8847360 sec
RG 101
DW 13.500 usec
DE 6.50 usec
TE 300.3 K
D1 2.00000000 sec
D11 0.03000000 sec
TD0 1
SFO1 125.7804233 MHz
NUC1 13C
P0 3.33 usec
P1 10.00 usec
PLW1 83.14099884 W
SFO2 500.1720007 MHz
NUC2 1H
CPDPRG[2 waltz65
PCPD2 80.00 usec
PLW2 20.93000031 W
PLW12 0.32703000 W
PLW13 0.16449000 W

F2 - Processing parameters
SI 32768
SF 125.7678468 MHz
WDW EM
SSB 0
LB 1.00 Hz
GB 0
PC 1.40

D10
C13CPD CDCl3 {D:\Spectra} nmr 26

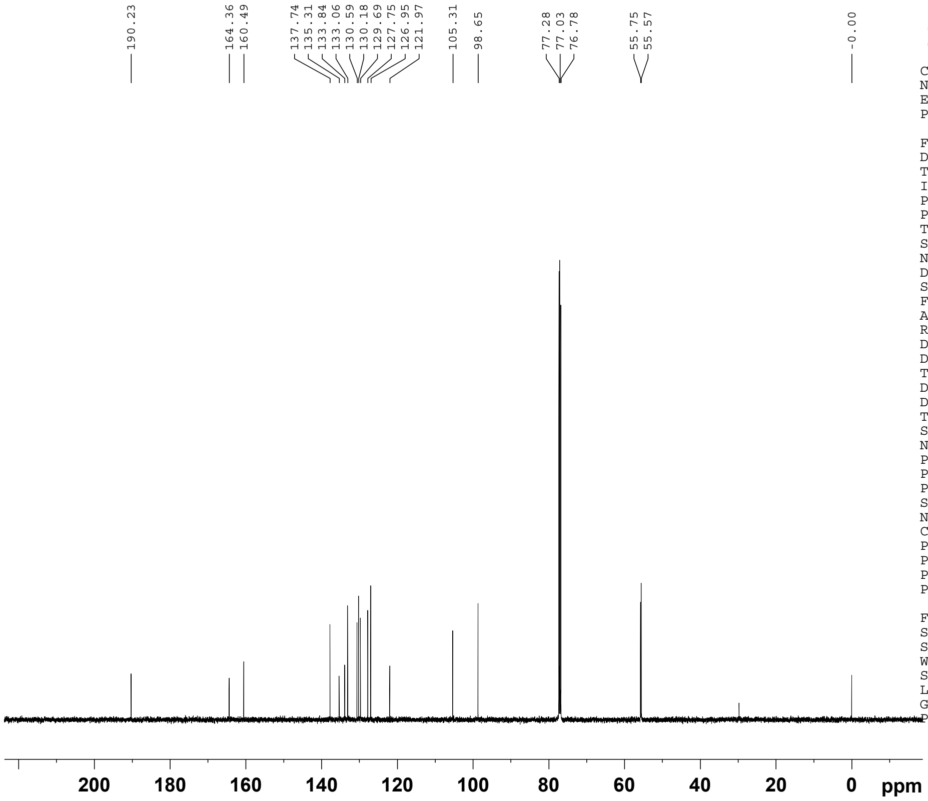

BRUKER
AVANCE NEO
500 MHz NMR SPECTROMETER
SAIF, PANJAB UNIVERSITY,
CHANDIGARH

Current Data Parameters
NAME Jun21-2022
EXPNO 261
PROCNO 1

F2 - Acquisition Parameters
Date_ 20220621
Time 16.45 h
INSTRUM Avance Neo 500
PROBHD Z119470_0333 (
PULPROG zgpg30
TD 65536
SOLVENT CDCl3
NS 261
DS 4
SWH 37037.035 Hz
FIDRES 1.130281 Hz
AQ 0.8847360 sec
RG 101
DW 13.500 usec
DE 6.50 usec
TE 300.3 K
D1 2.00000000 sec
D11 0.03000000 sec
TD0 1
SFO1 125.7804233 MHz
NUC1 13C
P0 3.33 usec
P1 10.00 usec
PLW1 83.14099884 W
SFO2 500.1720007 MHz
NUC2 1H
CPDPRG[2 waltz65
PCPD2 80.00 usec
PLW2 20.93000031 W
PLW12 0.32703000 W
PLW13 0.16449000 W

F2 - Processing parameters
SI 32768
SF 125.7678467 MHz
WDW EM
SSB 0
LB 1.00 Hz
GB 0
PC 1.40

D11

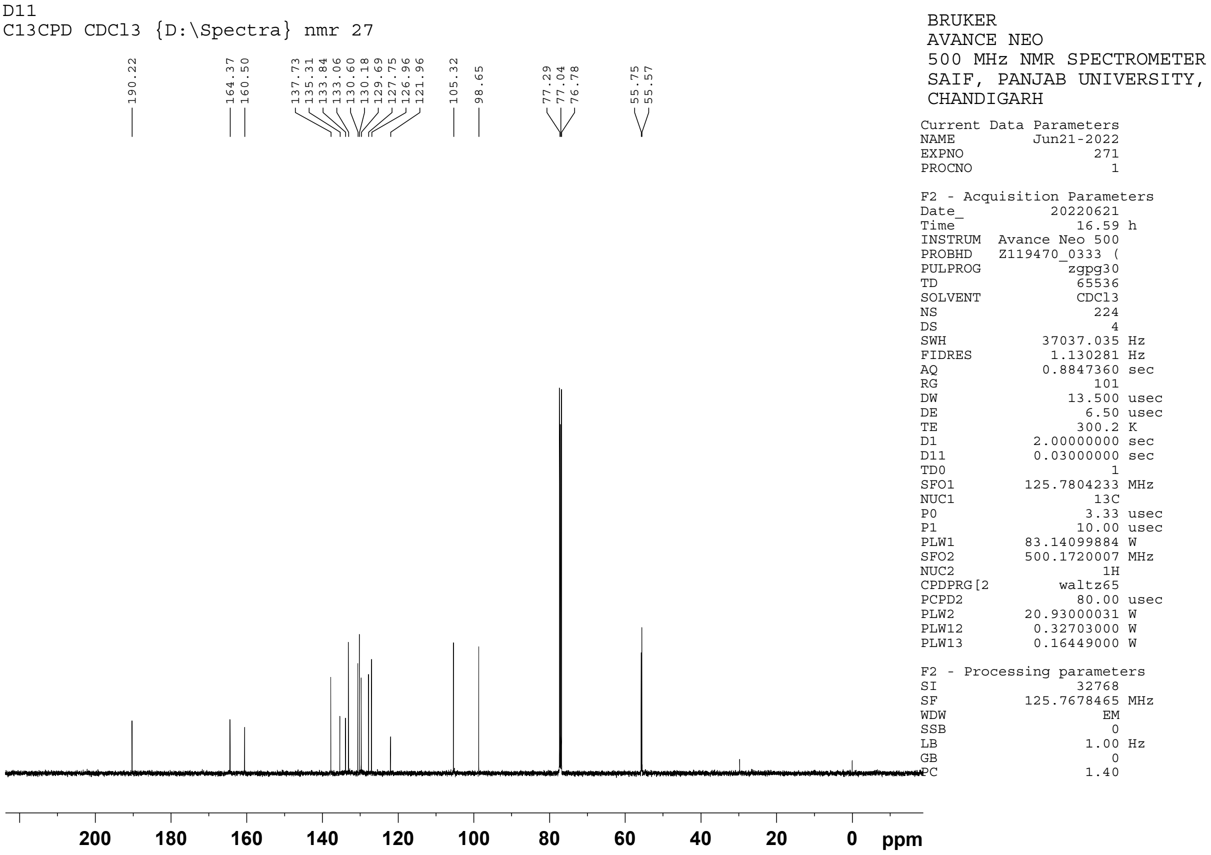
C13CPD CDCl3 {D:\Spectra} nmr 27
190.22
164.37
160.50
137.73
135.31
133.84
133.06
130.60
130.18
129.69
127.75
126.96
121.96
105.32
98.65
77.29
77.04
76.78
55.75
55.57
200 180 160 140 120 100 80 60 40 20 0 ppm
BRUKER
AVANCE NEO
500 MHz NMR SPECTROMETER
SAIF, PANJAB UNIVERSITY,
CHANDIGARH
Current Data Parameters
NAME Jun21-2022
EXPNO 271
PROCNO 1
F2 - Acquisition Parameters
Date_ 20220621
Time 16.59 h
INSTRUM Avance Neo 500
PROBHD Z119470_0333 (
PULPROG zgpg30
TD 65536
SOLVENT CDCl3
NS 224
DS 4
SWH 37037.035 Hz
FIDRES 1.130281 Hz
AQ 0.8847360 sec
RG 101
DW 13.500 usec
DE 6.50 usec
TE 300.2 K
D1 2.00000000 sec
D11 0.03000000 sec
TD0 1
SFO1 125.7804233 MHz
NUC1 13C
P0 3.33 usec
P1 10.00 usec
PLW1 83.14099884 W
SFO2 500.1720007 MHz
NUC2 1H
CPDPRG[2 waltz65
PCPD2 80.00 usec
PLW2 20.93000031 W
PLW12 0.32703000 W
PLW13 0.16449000 W
F2 - Processing parameters
SI 32768
SF 125.7678465 MHz
WDW EM
SSB 0
LB 1.00 Hz
GB 0
PC 1.40

D12

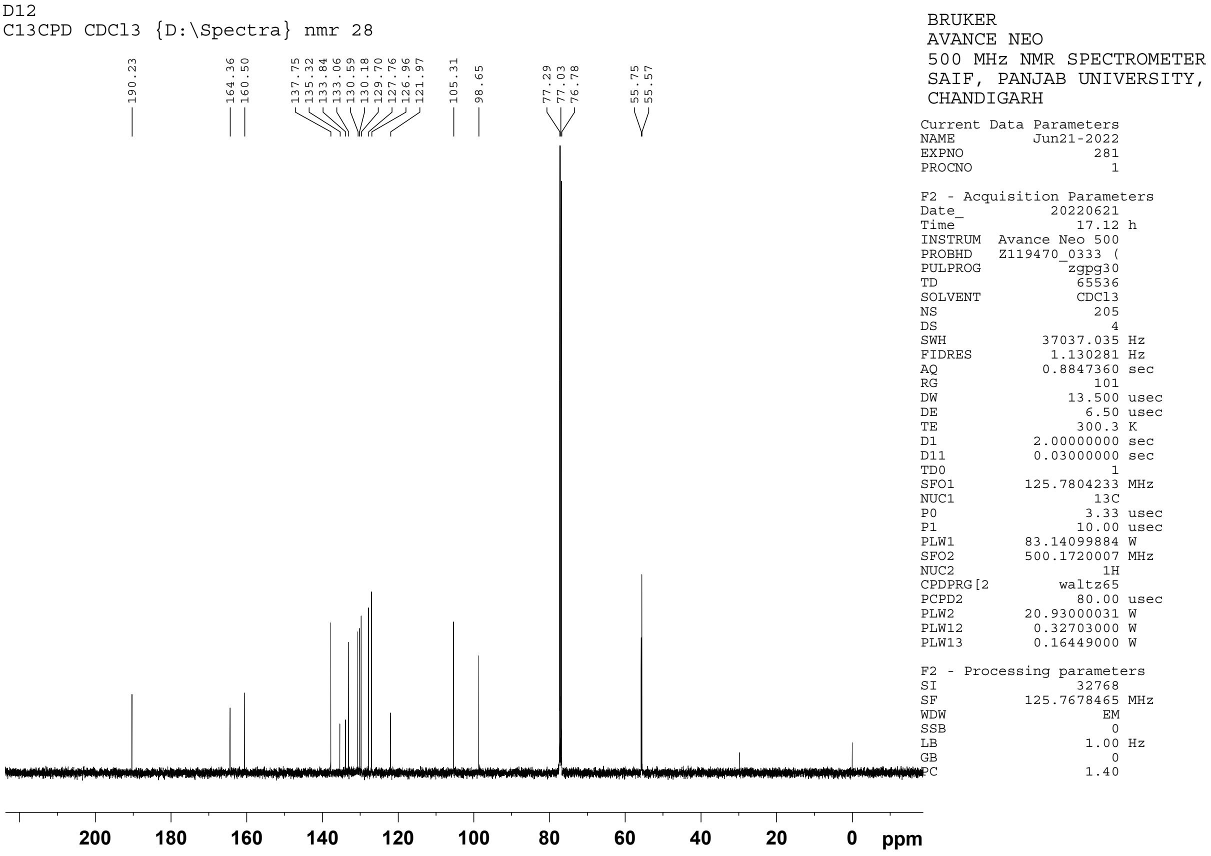
C13CPD CDCl3 {D:\Spectra} nmr 28
190.23
164.36
160.50
137.75
135.32
133.84
133.06
130.59
130.18
129.70
127.76
126.96
121.97
105.31
98.65
77.29
77.03
76.78
55.75
55.57
200 180 160 140 120 100 80 60 40 20 0 ppm
BRUKER
AVANCE NEO
500 MHz NMR SPECTROMETER
SAIF, PANJAB UNIVERSITY,
CHANDIGARH
Current Data Parameters
NAME Jun21-2022
EXPNO 281
PROCNO 1
F2 - Acquisition Parameters
Date_ 20220621
Time 17.12 h
INSTRUM Avance Neo 500
PROBHD Z119470_0333 (
PULPROG zgpg30
TD 65536
SOLVENT CDCl3
NS 205
DS 4
SWH 37037.035 Hz
FIDRES 1.130281 Hz
AQ 0.8847360 sec
RG 101
DW 13.500 usec
DE 6.50 usec
TE 300.3 K
D1 2.00000000 sec
D11 0.03000000 sec
TD0 1
SFO1 125.7804233 MHz
NUC1 13C
P0 3.33 usec
P1 10.00 usec
PLW1 83.14099884 W
SFO2 500.1720007 MHz
NUC2 1H
CPDPRG[2 waltz65
PCPD2 80.00 usec
PLW2 20.93000031 W
PLW12 0.32703000 W
PLW13 0.16449000 W
F2 - Processing parameters
SI 32768
SF 125.7678465 MHz
WDW EM
SSB 0
LB 1.00 Hz
GB 0
PC 1.40

D13
C13CPD CDCl3 {D:\Spectra} nmr 29

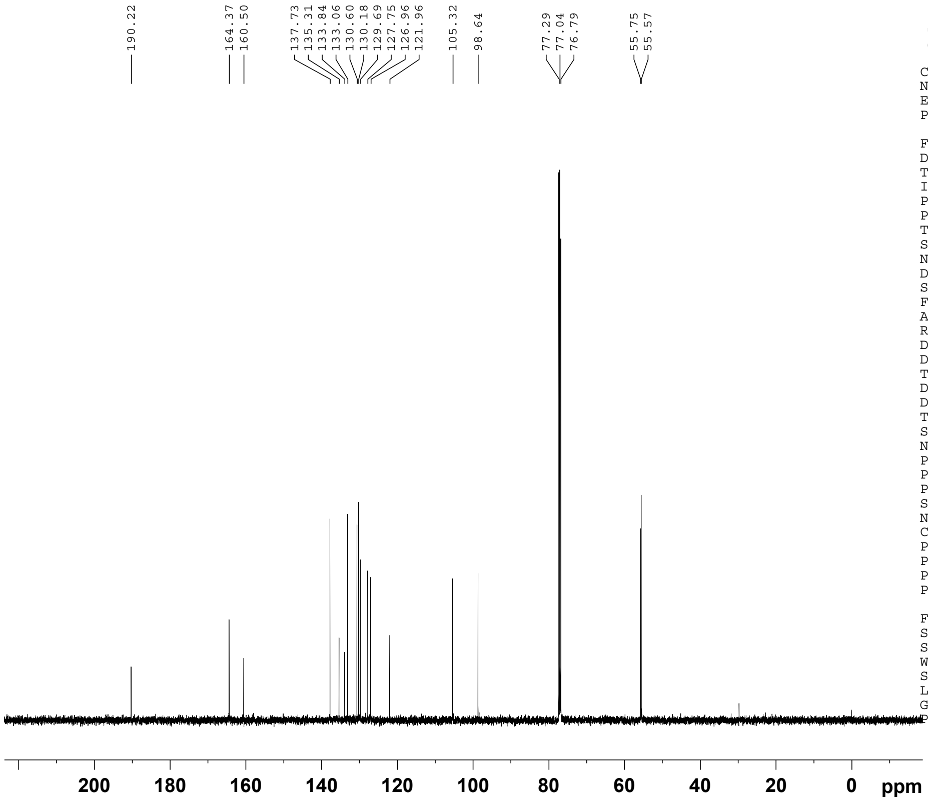

BRUKER
AVANCE NEO
500 MHz NMR SPECTROMETER
SAIF, PANJAB UNIVERSITY,
CHANDIGARH

Current Data Parameters
NAME Jun21-2022
EXPNO 291
PROCNO 1

F2 - Acquisition Parameters
Date_ 20220621
Time 17.23 h
INSTRUM Avance Neo 500
PROBHD Z119470_0333 (
PULPROG zgpg30
TD 65536
SOLVENT CDCl3
NS 156
DS 4
SWH 37037.035 Hz
FIDRES 1.130281 Hz
AQ 0.8847360 sec
RG 101
DW 13.500 usec
DE 6.50 usec
TE 300.2 K
D1 2.00000000 sec
D11 0.03000000 sec
TD0 1
SFO1 125.7804233 MHz
NUC1 13C
P0 3.33 usec
P1 10.00 usec
PLW1 83.14099884 W
SFO2 500.1720007 MHz
NUC2 1H
CPDPRG[2 waltz65
PCPD2 80.00 usec
PLW2 20.93000031 W
PLW12 0.32703000 W
PLW13 0.16449000 W

F2 - Processing parameters
SI 32768
SF 125.7678465 MHz
WDW EM
SSB 0
LB 1.00 Hz
GB 0
PC 1.40

D14
C13CPD CDCl3 {D:\Spectra} nmr 30

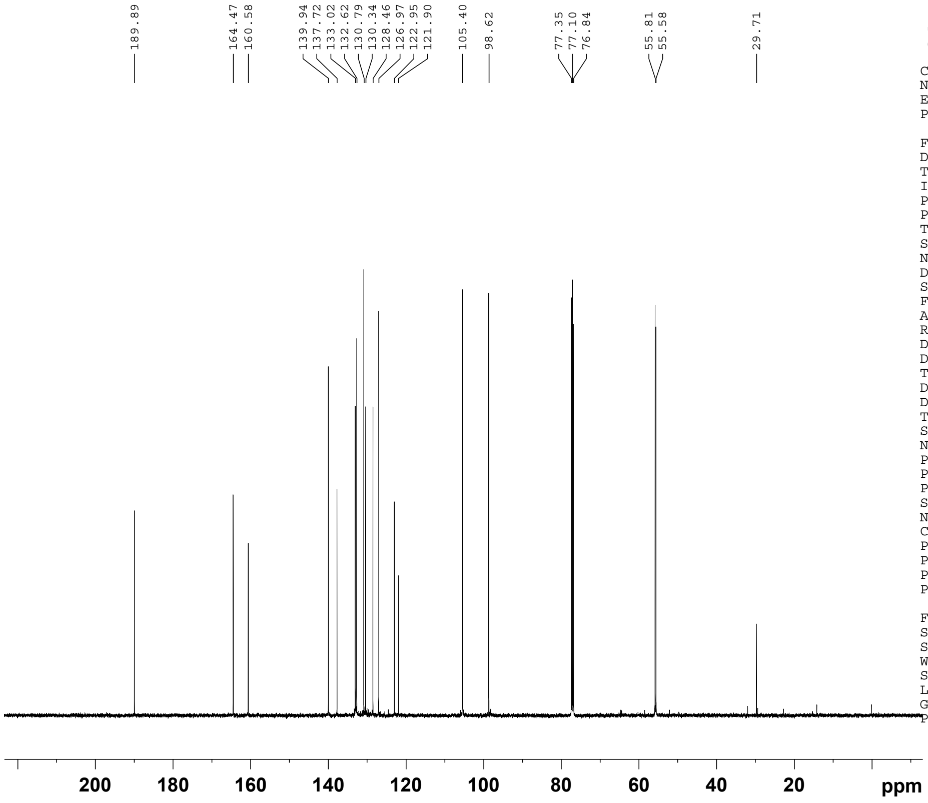

BRUKER
AVANCE NEO
500 MHz NMR SPECTROMETER
SAIF, PANJAB UNIVERSITY,
CHANDIGARH

Current Data Parameters
NAME Jun21-2022
EXPNO 301
PROCNO 1

F2 - Acquisition Parameters
Date_ 20220621
Time 17.52 h
INSTRUM Avance Neo 500
PROBHD Z119470_0333 (
PULPROG zgpg30
TD 65536
SOLVENT CDCl3
NS 512
DS 4
SWH 37037.035 Hz
FIDRES 1.130281 Hz
AQ 0.8847360 sec
RG 101
DW 13.500 usec
DE 6.50 usec
TE 300.1 K
D1 2.00000000 sec
D11 0.03000000 sec
TD0 1
SFO1 125.7804233 MHz
NUC1 13C
P0 3.33 usec
P1 10.00 usec
PLW1 83.14099884 W
SFO2 500.1720007 MHz
NUC2 1H
CPDPRG[2 waltz65
PCPD2 80.00 usec
PLW2 20.93000031 W
PLW12 0.32703000 W
PLW13 0.16449000 W

F2 - Processing parameters
SI 32768
SF 125.7678465 MHz
WDW EM
SSB 0
LB 1.00 Hz
GB 0
PC 1.40

D16
C13CPD CDCl3 {D:\Spectra} nmr 32

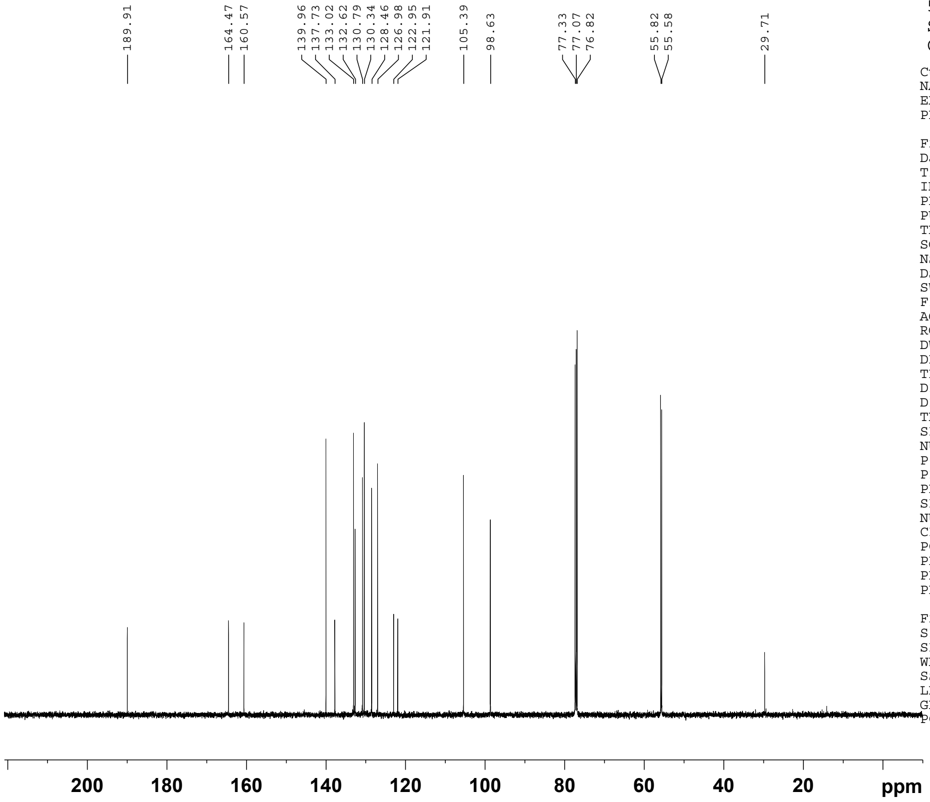

BRUKER
AVANCE NEO
500 MHz NMR SPECTROMETER
SAIF, PANJAB UNIVERSITY,
CHANDIGARH

Current Data Parameters
NAME Jun21-2022
EXPNO 321
PROCNO 1

F2 - Acquisition Parameters
Date_ 20220622
Time 10.51 h
INSTRUM Avance Neo 500
PROBHD Z119470_0333 (
PULPROG zgpg30
TD 65536
SOLVENT CDCl3
NS 156
DS 4
SWH 37037.035 Hz
FIDRES 1.130281 Hz
AQ 0.8847360 sec
RG 101
DW 13.500 usec
DE 6.50 usec
TE 300.2 K
D1 2.00000000 sec
D11 0.03000000 sec
TD0 1
SFO1 125.7804233 MHz
NUC1 13C
P0 3.33 usec
P1 10.00 usec
PLW1 83.14099884 W
SFO2 500.1720007 MHz
NUC2 1H
CPDPRG[2 waltz65
PCPD2 80.00 usec
PLW2 20.93000031 W
PLW12 0.32703000 W
PLW13 0.16449000 W

F2 - Processing parameters
SI 32768
SF 125.7678465 MHz
WDW EM
SSB 0
LB 1.00 Hz
GB 0
PC 1.40

D17
C13CPD CDCl3 {D:\Spectra} nmr 33

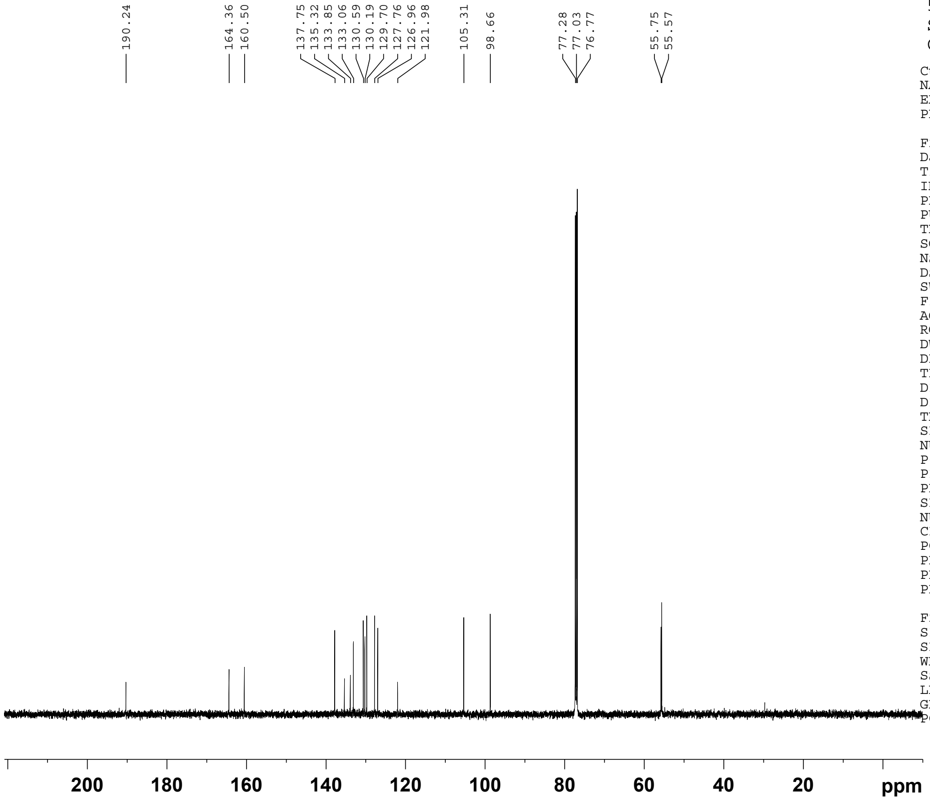

BRUKER
AVANCE NEO
500 MHz NMR SPECTROMETER
SAIF, PANJAB UNIVERSITY,
CHANDIGARH

Current Data Parameters
NAME Jun21-2022
EXPNO 331
PROCNO 1

F2 - Acquisition Parameters
Date_ 20220622
Time 11.07 h
INSTRUM Avance Neo 500
PROBHD Z119470_0333 (
PULPROG zgpg30
TD 65536
SOLVENT CDCl3
NS 270
DS 4
SWH 37037.035 Hz
FIDRES 1.130281 Hz
AQ 0.8847360 sec
RG 101
DW 13.500 usec
DE 6.50 usec
TE 300.1 K
D1 2.00000000 sec
D11 0.03000000 sec
TD0 1
SFO1 125.7804233 MHz
NUC1 13C
P0 3.33 usec
P1 10.00 usec
PLW1 83.14099884 W
SFO2 500.1720007 MHz
NUC2 1H
CPDPRG[2 waltz65
PCPD2 80.00 usec
PLW2 20.93000031 W
PLW12 0.32703000 W
PLW13 0.16449000 W

F2 - Processing parameters
SI 32768
SF 125.7678465 MHz
WDW EM
SSB 0
LB 1.00 Hz
GB 0
PC 1.40

D18
C13CPD CDCl3 {D:\Spectra} nmr 34

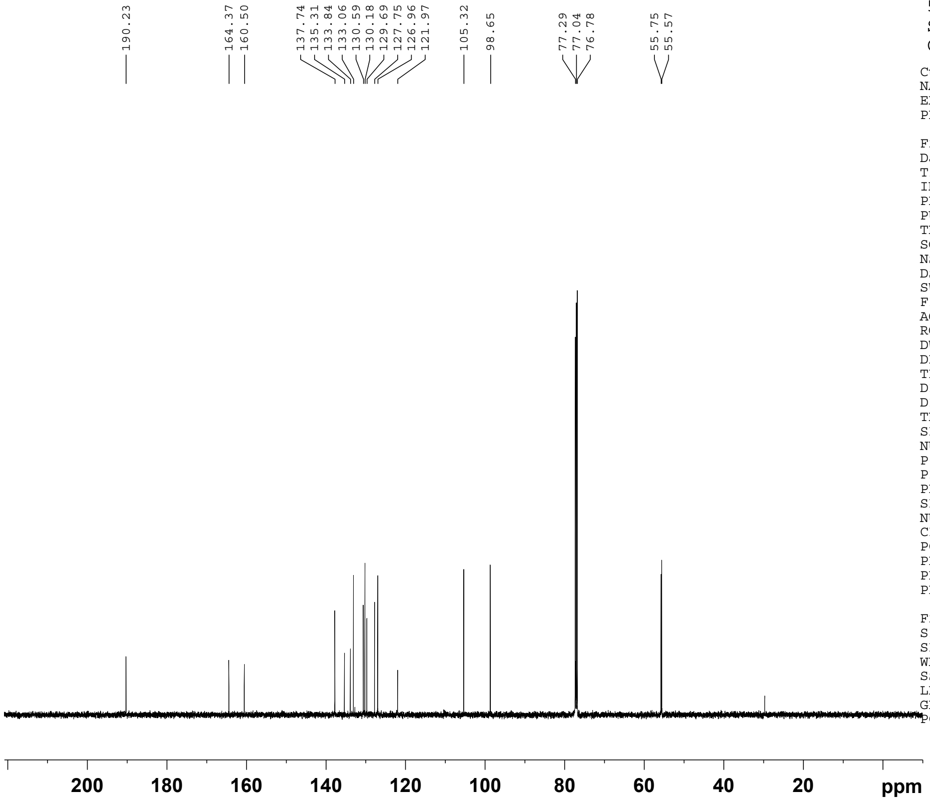

BRUKER
AVANCE NEO
500 MHz NMR SPECTROMETER
SAIF, PANJAB UNIVERSITY,
CHANDIGARH

```
Current Data Parameters
NAME          Jun21-2022
EXPNO                341
PROCNO                 1

F2 - Acquisition Parameters
Date_           20220622
Time               11.59 h
INSTRUM  Avance Neo 500
PROBHD   Z119470_0333 (
PULPROG          zgpg30
TD                65536
SOLVENT           CDCl3
NS                  176
DS                    4
SWH           37037.035 Hz
FIDRES         1.130281 Hz
AQ            0.8847360 sec
RG                  101
DW               13.500 usec
DE                 6.50 usec
TE                300.1 K
D1           2.00000000 sec
D11          0.03000000 sec
TD0                   1
SFO1        125.7804233 MHz
NUC1                13C
P0                 3.33 usec
P1                10.00 usec
PLW1        83.14099884 W
SFO2        500.1720007 MHz
NUC2                 1H
CPDPRG[2        waltz65
PCPD2             80.00 usec
PLW2        20.93000031 W
PLW12        0.32703000 W
PLW13        0.16449000 W

F2 - Processing parameters
SI                32768
SF          125.7678465 MHz
WDW                  EM
SSB                   0
LB                 1.00 Hz
GB                    0
PC                 1.40
```

D20
C13CPD CDCl3 {D:\Spectra} nmr 36

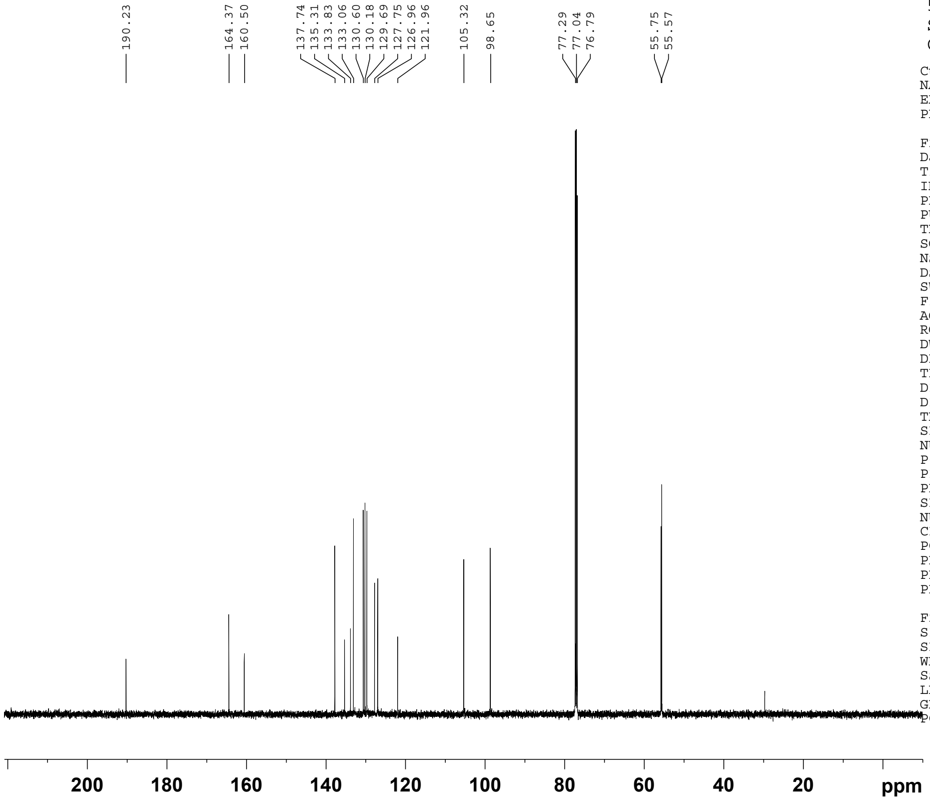

BRUKER
AVANCE NEO
500 MHz NMR SPECTROMETER
SAIF, PANJAB UNIVERSITY,
CHANDIGARH

```
Current Data Parameters
NAME          Jun21-2022
EXPNO                361
PROCNO                 1

F2 - Acquisition Parameters
Date_           20220622
Time               12.40 h
INSTRUM  Avance Neo 500
PROBHD   Z119470_0333 (
PULPROG          zgpg30
TD                65536
SOLVENT           CDCl3
NS                  240
DS                    4
SWH           37037.035 Hz
FIDRES         1.130281 Hz
AQ            0.8847360 sec
RG                  101
DW               13.500 usec
DE                 6.50 usec
TE                300.2 K
D1           2.00000000 sec
D11          0.03000000 sec
TD0                   1
SFO1        125.7804233 MHz
NUC1                13C
P0                 3.33 usec
P1                10.00 usec
PLW1        83.14099884 W
SFO2        500.1720007 MHz
NUC2                 1H
CPDPRG[2        waltz65
PCPD2             80.00 usec
PLW2        20.93000031 W
PLW12        0.32703000 W
PLW13        0.16449000 W

F2 - Processing parameters
SI                32768
SF          125.7678465 MHz
WDW                  EM
SSB                   0
LB                 1.00 Hz
GB                    0
PC                 1.40
```

Figure S3. MASS Spectra

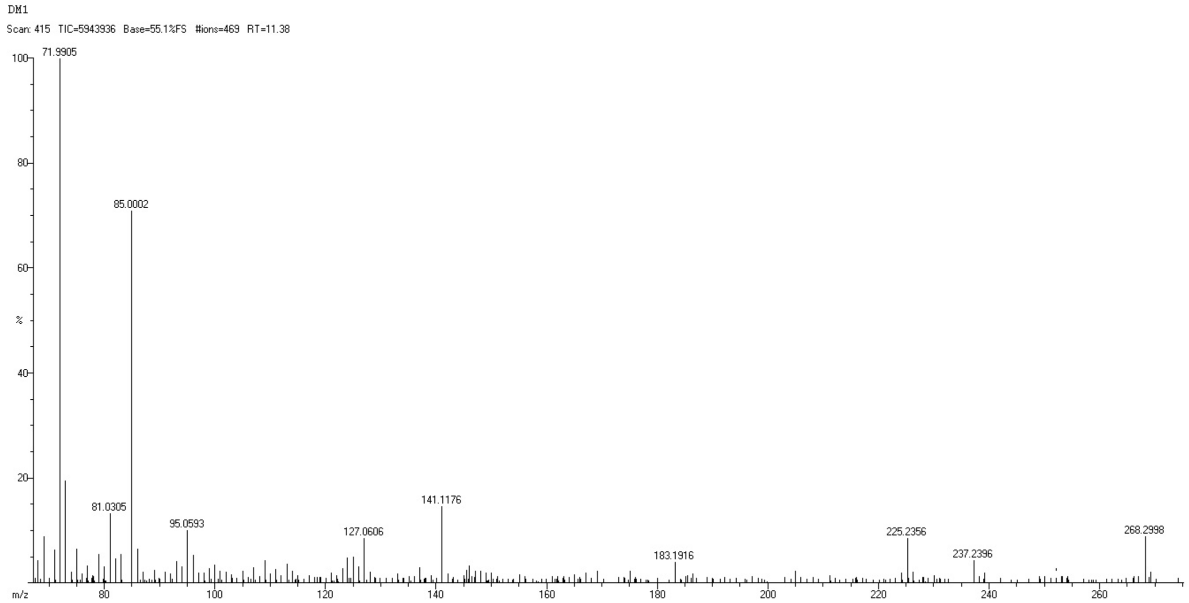

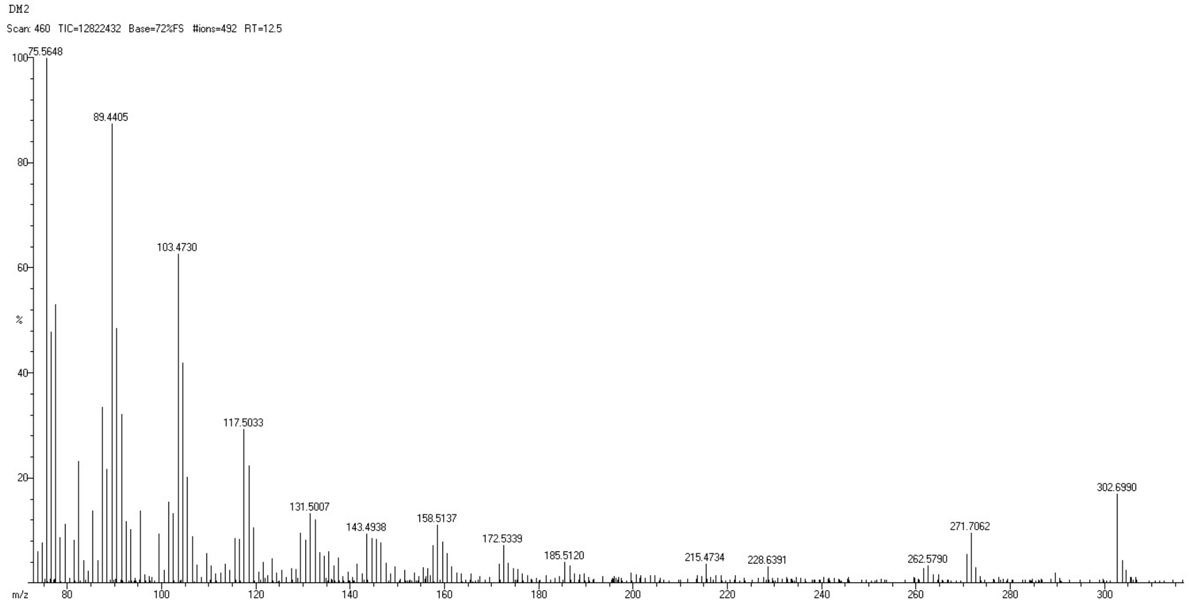

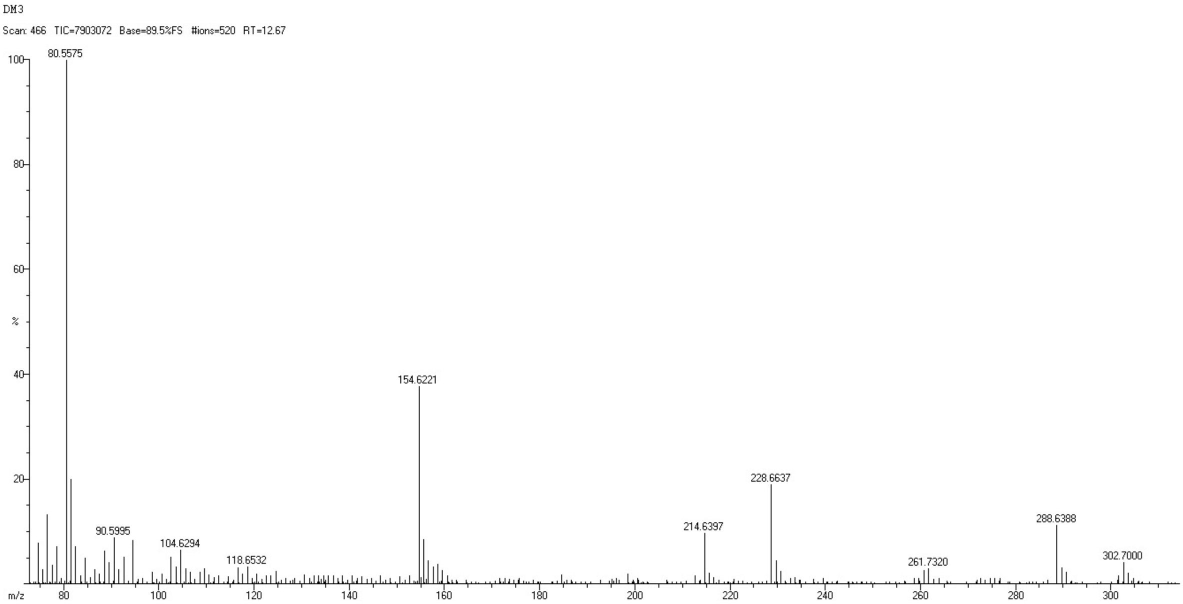
DM3
Scan: 466 TIC=7903072 Base=89.5%FS #ions=520 RT=12.67
80.5575
90.5995
104.6294
118.6532
154.6221
214.6397
228.6637
261.7320
288.6388
302.7000
%
m/z

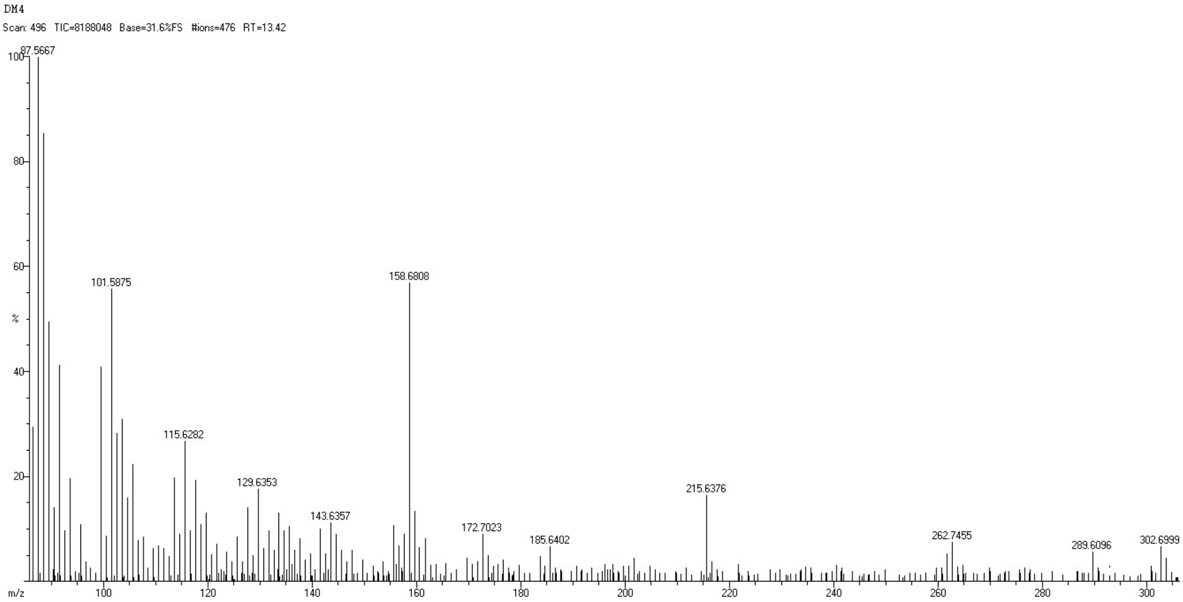
DM4
Scan: 496 TIC=8188048 Base=31.6%FS #ions=476 RT=13.42
87.5667
101.5875
115.6282
129.6353
143.6357
158.6808
172.7023
185.6402
215.6376
262.7455
289.6096
302.6999
%
m/z

DM5

Scan: 504 TIC=8003024 Base=76%FS #ions=512 RT=13.62

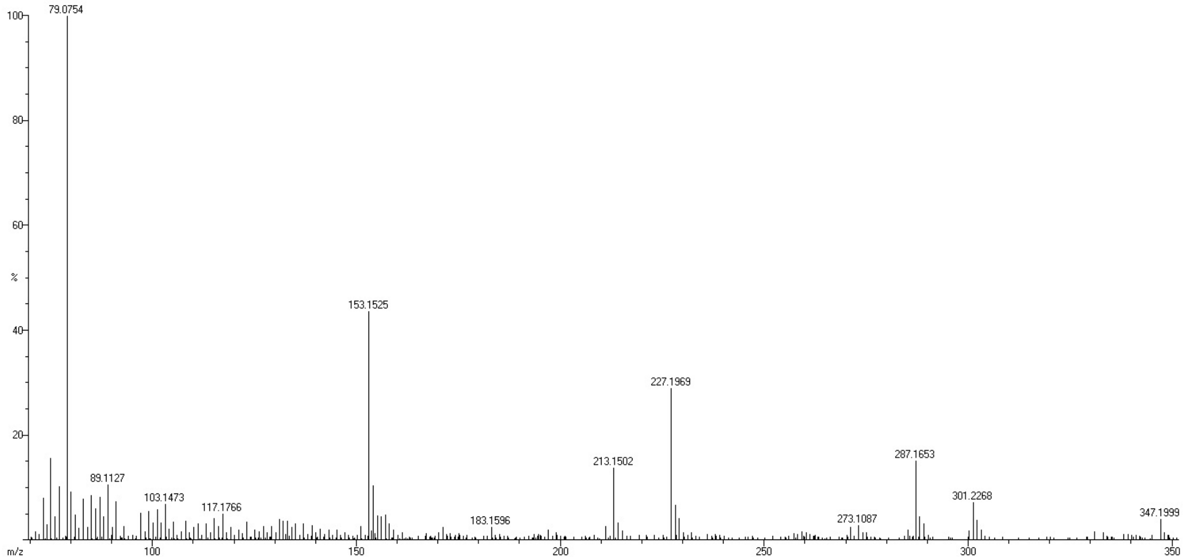

DM6

Scan: 549 TIC=7095840 Base=56.1%FS #ions=516 RT=14.73

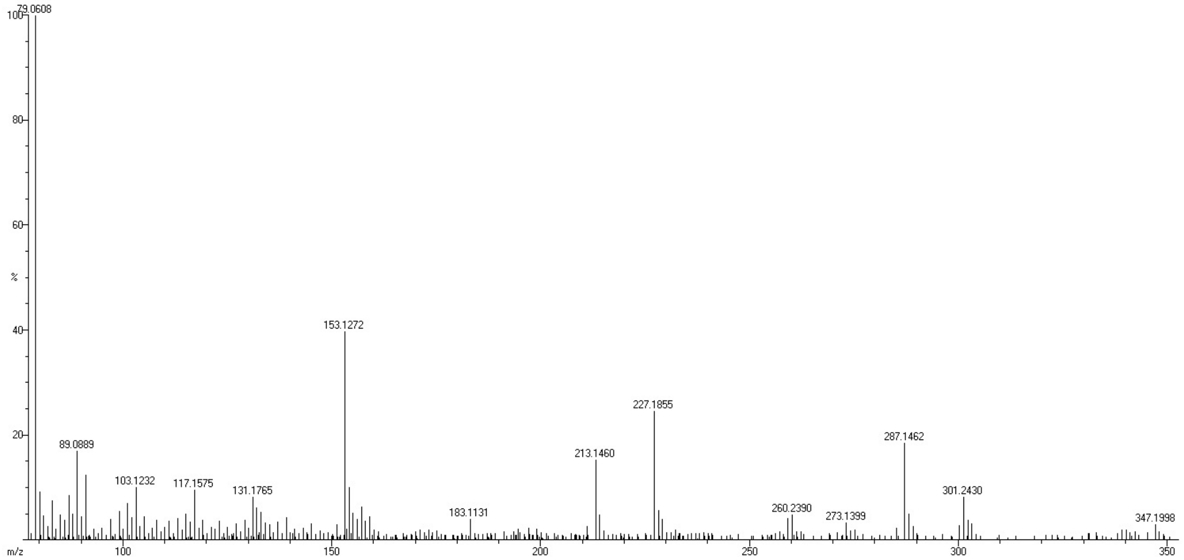

DM7

Scan: 609 TIC=6551136 Base=23.1%FS #ions=522 RT=16.25

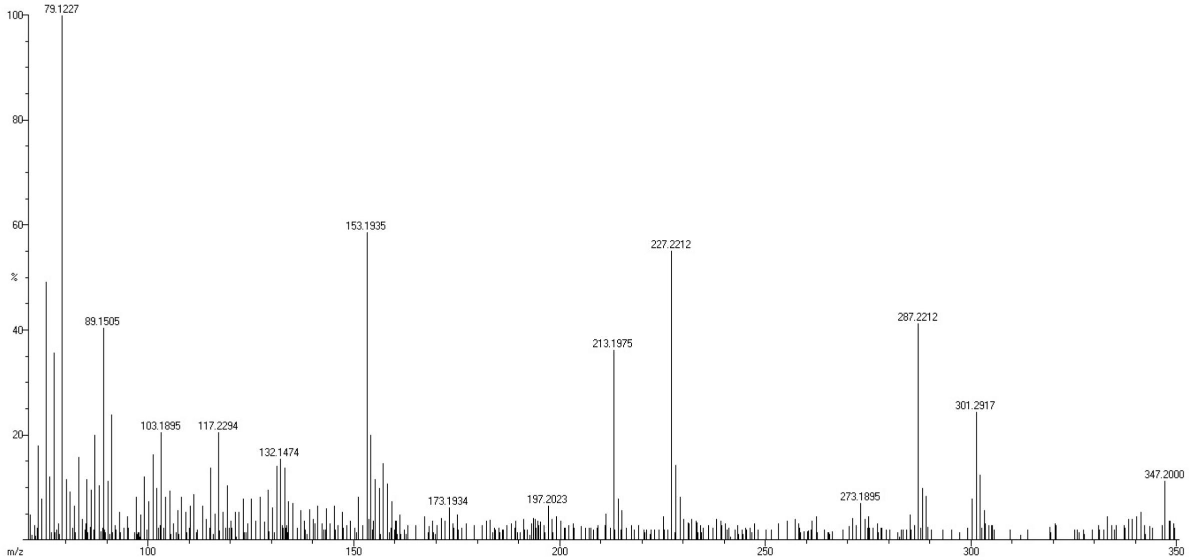

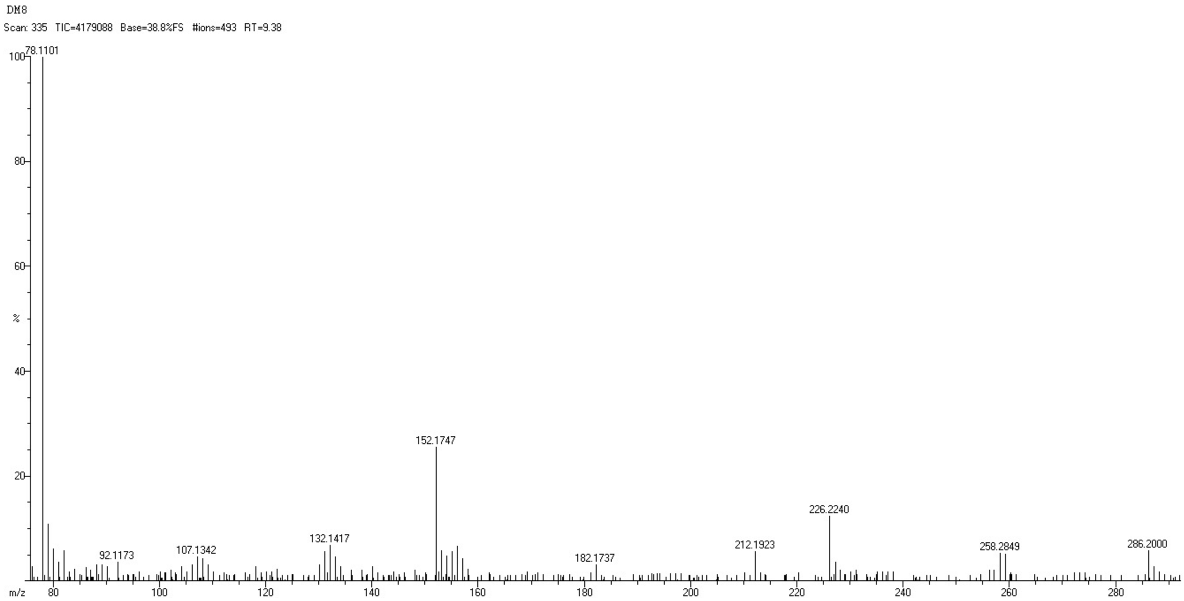
DM8
Scan: 335 TIC=4179088 Base=38.8%FS #ions=493 RT=9.38
78.1101
92.1173
107.1342
132.1417
152.1747
182.1737
212.1923
226.2240
258.2849
286.2000
%
m/z

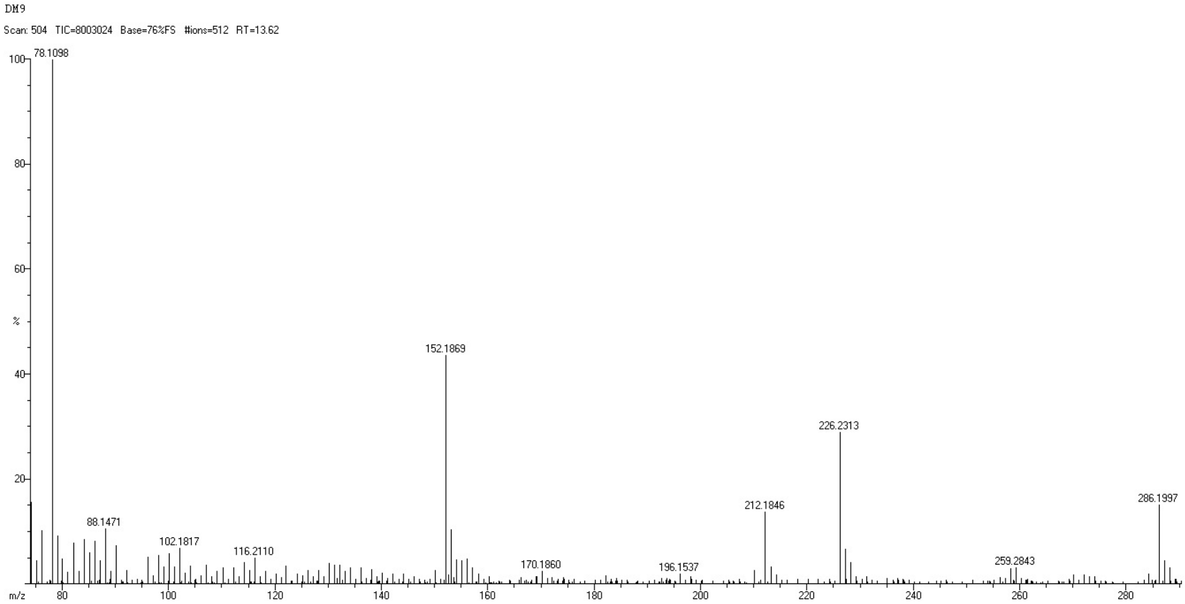
DM9
Scan: 504 TIC=8003024 Base=76%FS #ions=512 RT=13.62
78.1098
88.1471
102.1817
116.2110
152.1869
170.1860
196.1537
212.1846
226.2313
259.2843
286.1997
%
m/z

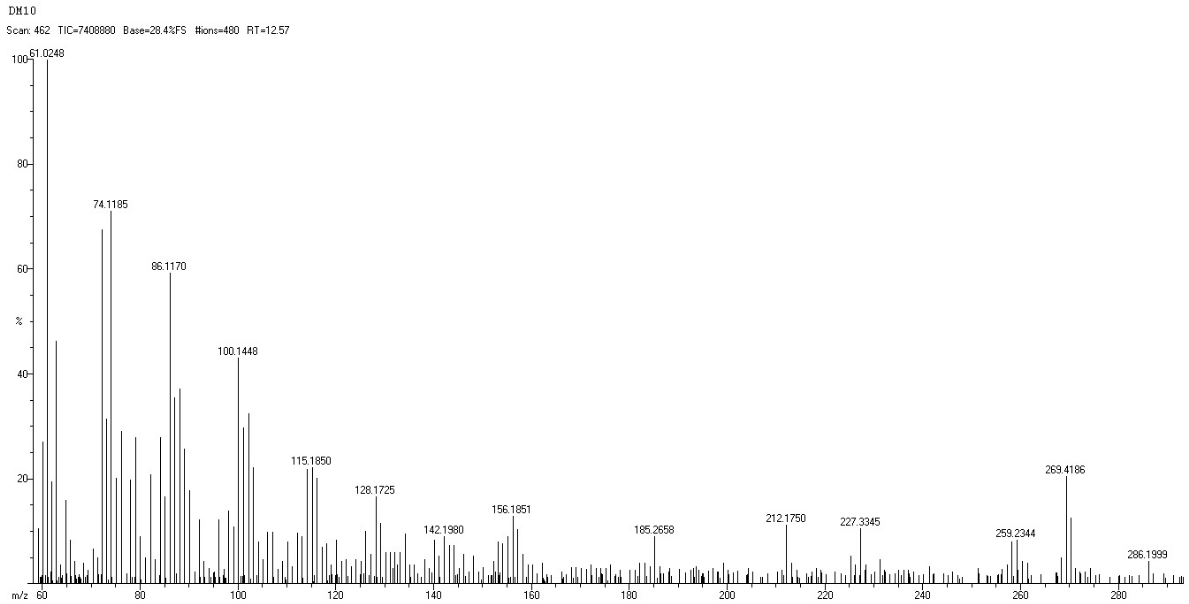
DM10
Scan: 462 TIC=7408880 Base=28.4%FS #ions=480 RT=12.57
61.0248
74.1185
86.1170
100.1448
115.1850
128.1725
142.1980
156.1851
185.2658
212.1750
227.3345
259.2344
269.4186
286.1999
%
m/z

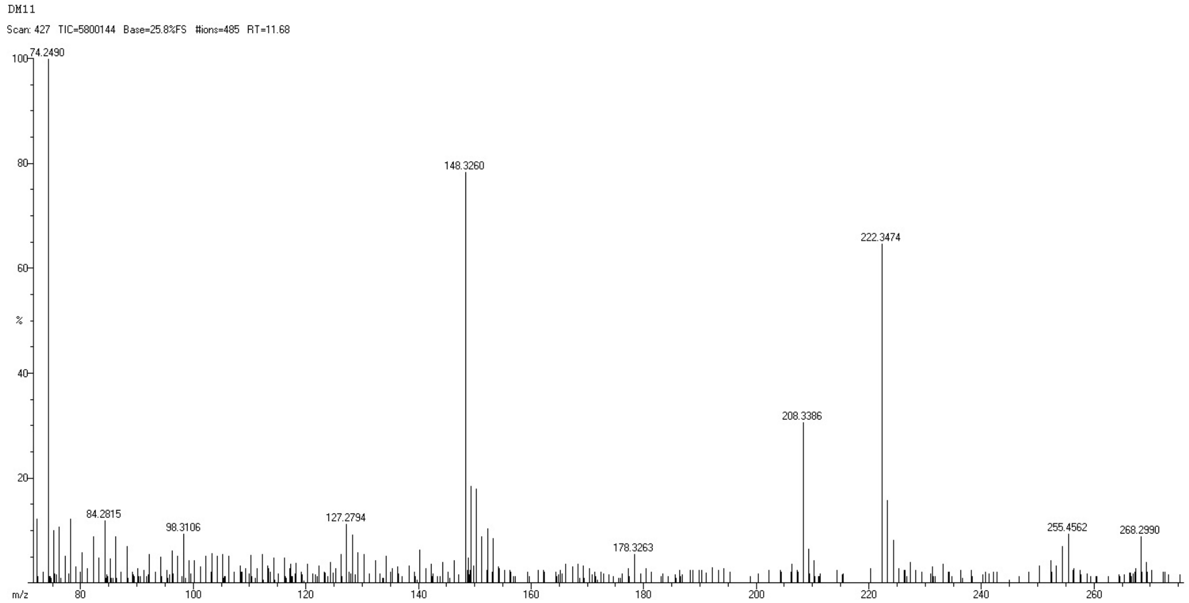
DM11
Scan: 427 TIC=5800144 Base=25.8%FS #ions=485 RT=11.68
74.2490
148.3260
222.3474
208.3386
84.2815
98.3106
127.2794
178.3263
255.4562
268.2990
%
m/z

DM12

Scan: 504 TIC=8003024 Base=76%FS #ions=512 RT=13.62

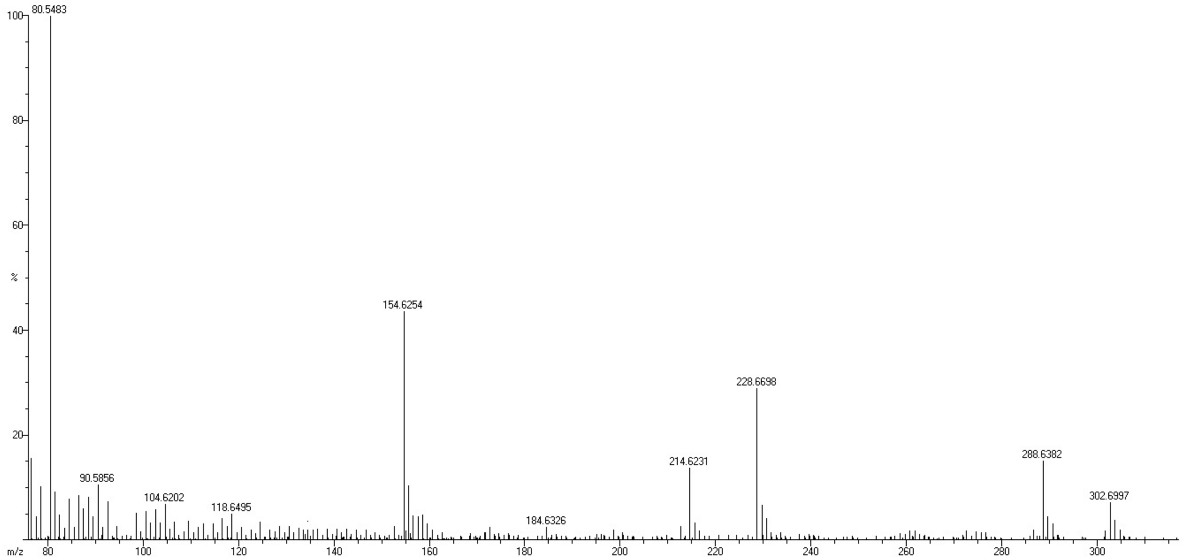

DM13

Scan: 549 TIC=7095840 Base=56.1%FS #ions=516 RT=14.73

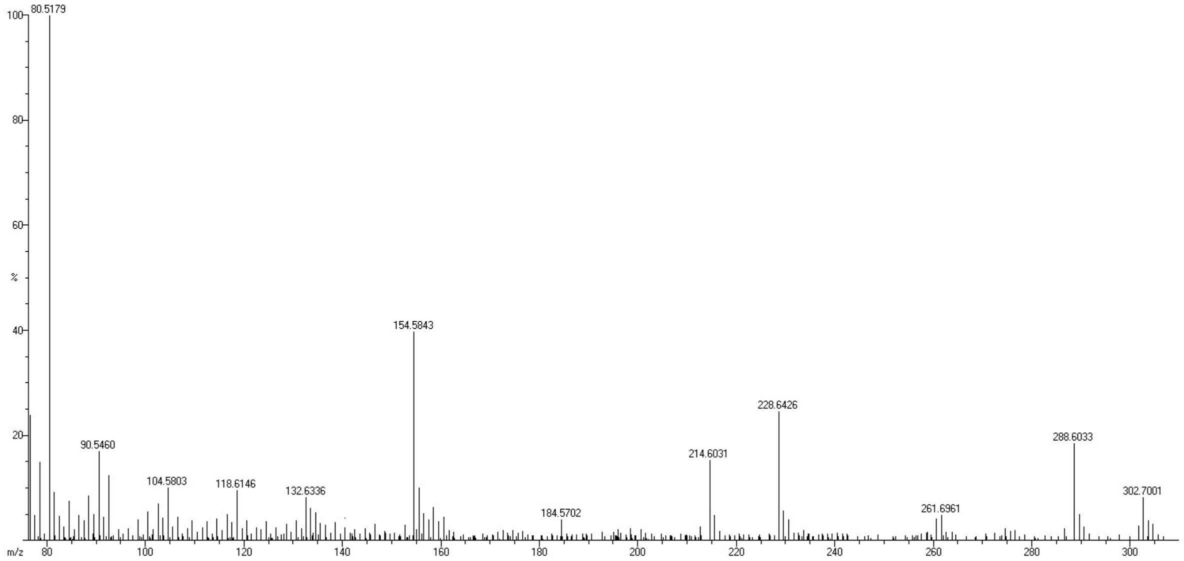

DM14

Scan: 609 TIC=6551136 Base=23.1%FS #ions=522 RT=16.25

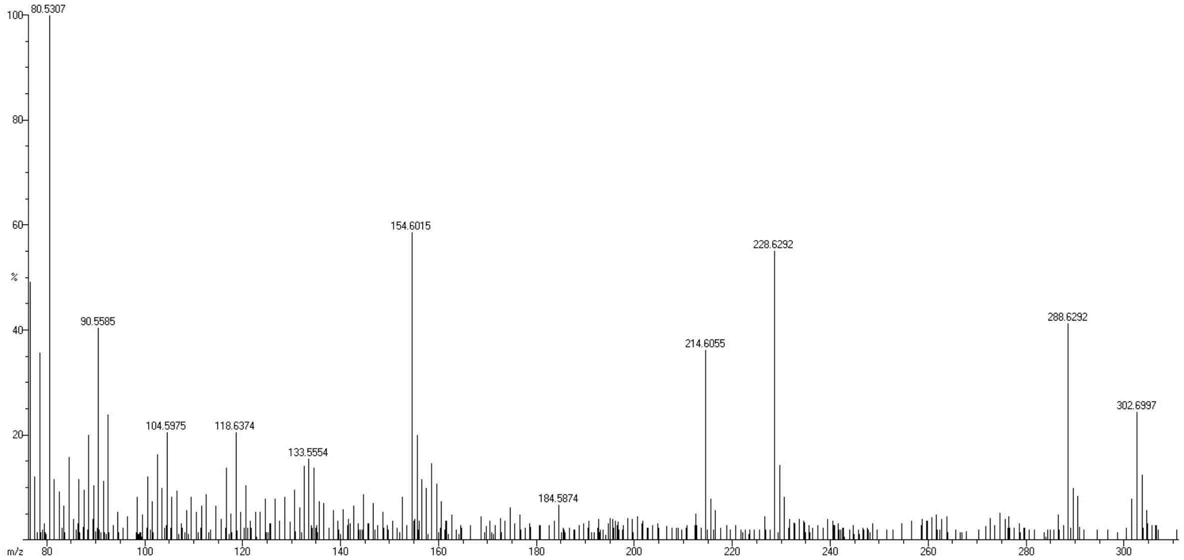

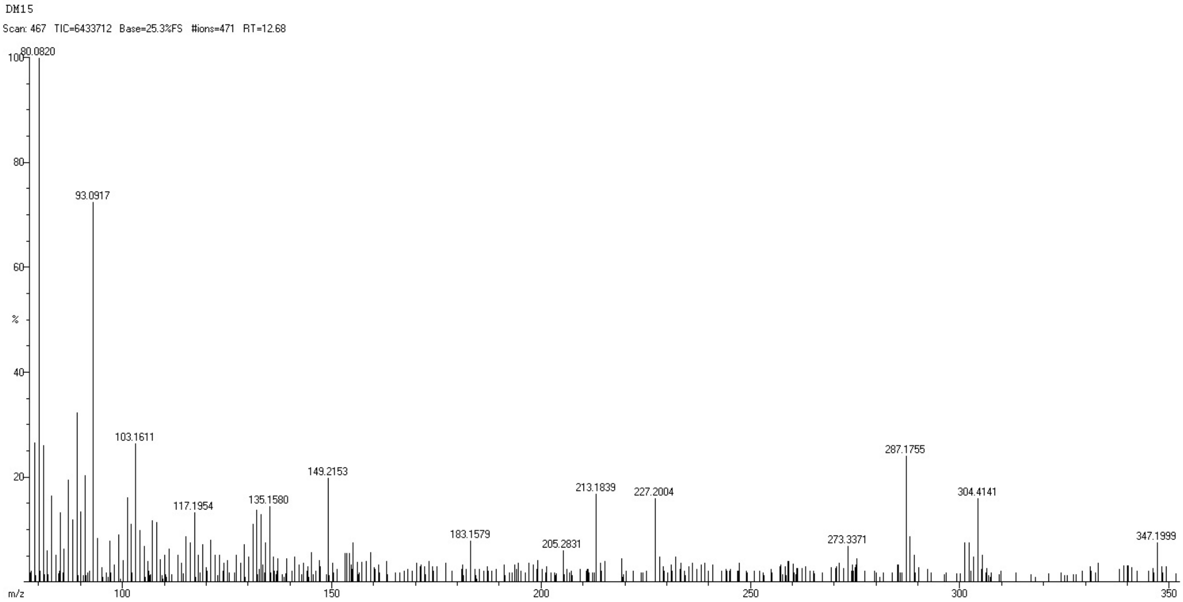
DM15
Scan: 467 TIC=6433712 Base=25.3%FS #ions=471 RT=12.68
80.0820
93.0917
103.1611
117.1954
135.1580
149.2153
183.1579
205.2831
213.1839
227.2004
273.3371
287.1755
304.4141
347.1999
%
m/z

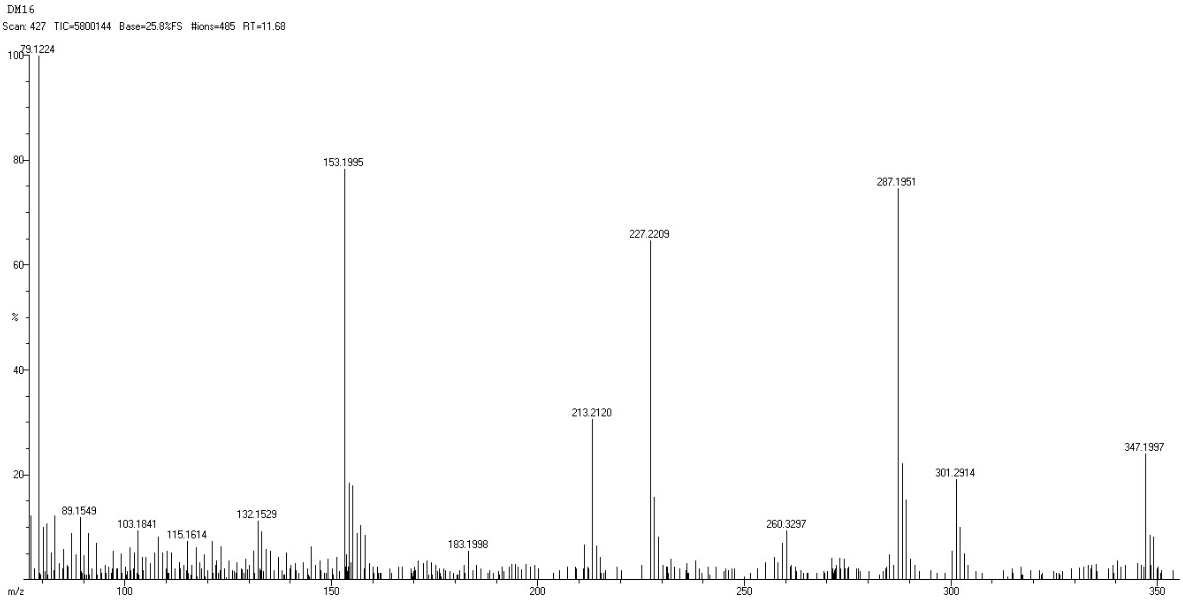
DM16
Scan: 427 TIC=5800144 Base=25.8%FS #ions=485 RT=11.68
79.1224
153.1995
287.1951
227.2209
213.2120
347.1997
301.2914
89.1549
103.1841
115.1614
132.1529
183.1998
260.3297
%
m/z

DM17

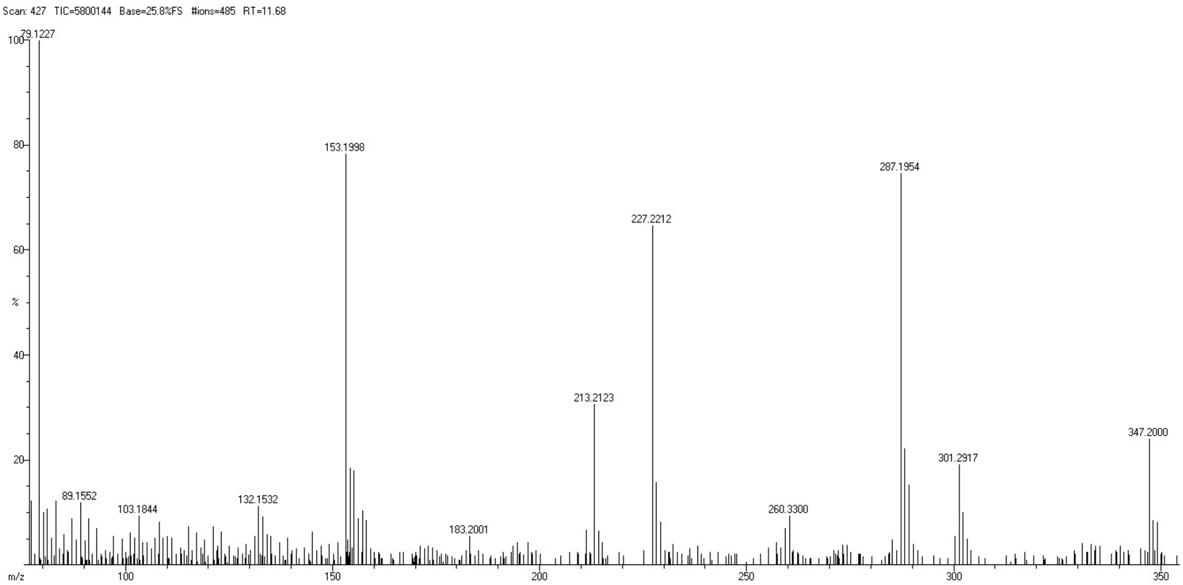
Scan: 427 TIC=5800144 Base=25.8%FS #ions=485 RT=11.68
79.1227
153.1998
287.1954
227.2212
213.2123
347.2000
301.2917
89.1552
103.1844
132.1532
260.3300
183.2001
%
m/z

DM18

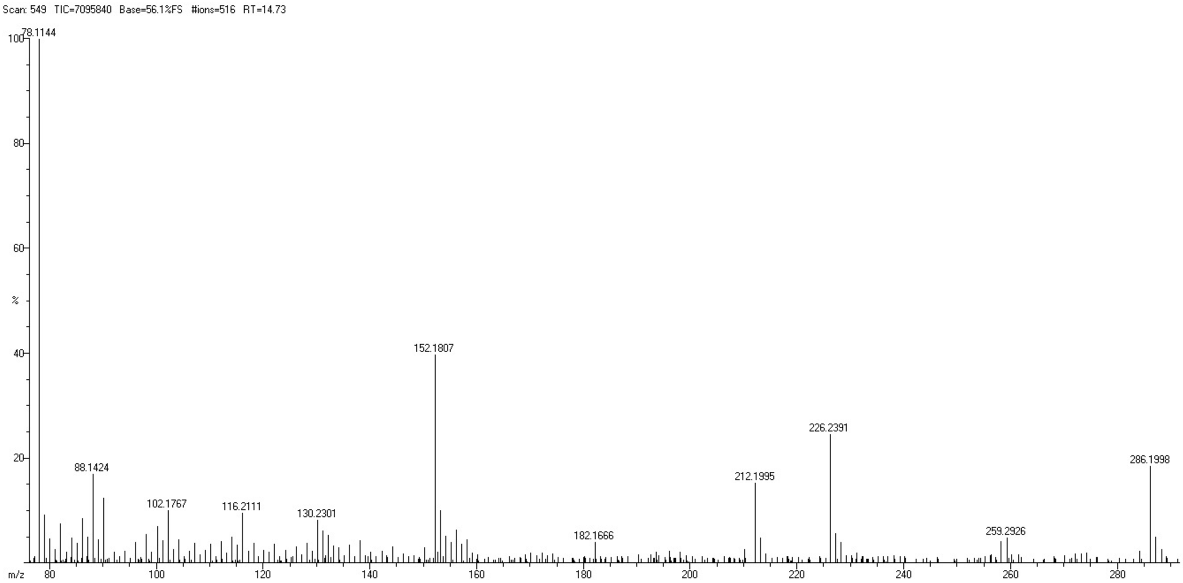
Scan: 549 TIC=7095840 Base=56.1%FS #ions=516 RT=14.73
78.1144
152.1807
226.2391
286.1998
88.1424
212.1995
102.1767
116.2111
130.2301
259.2926
182.1666
%
m/z

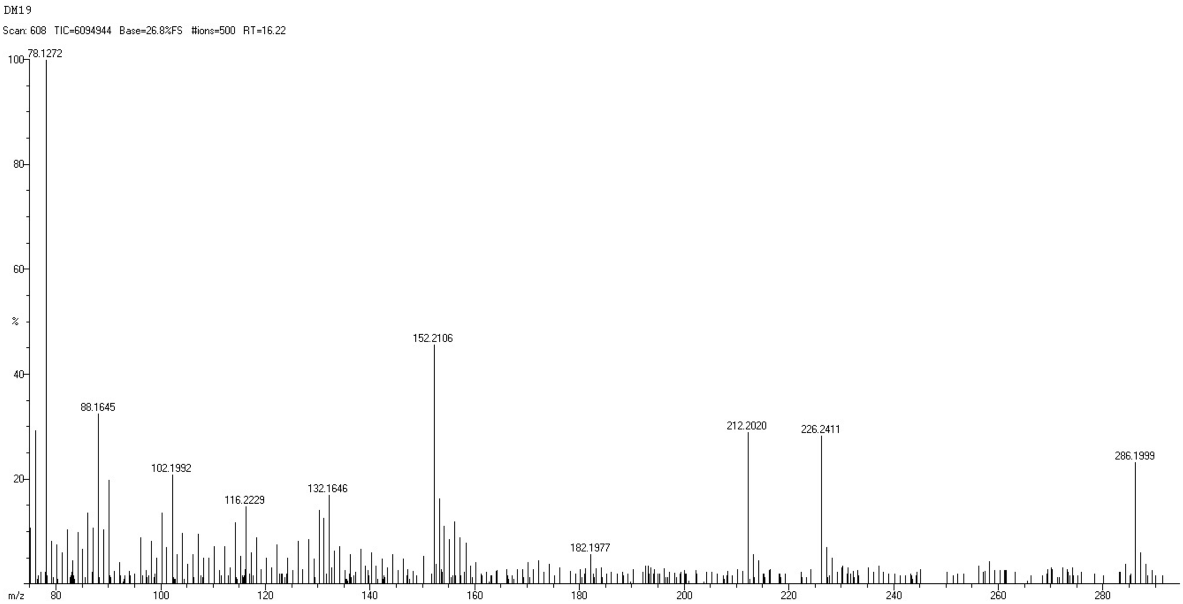
DM19
Scan: 608 TIC=6094944 Base=26.8%FS #ions=500 RT=16.22
78.1272
88.1645
102.1992
116.2229
132.1646
152.2106
182.1977
212.2020
226.2411
286.1999
%
m/z

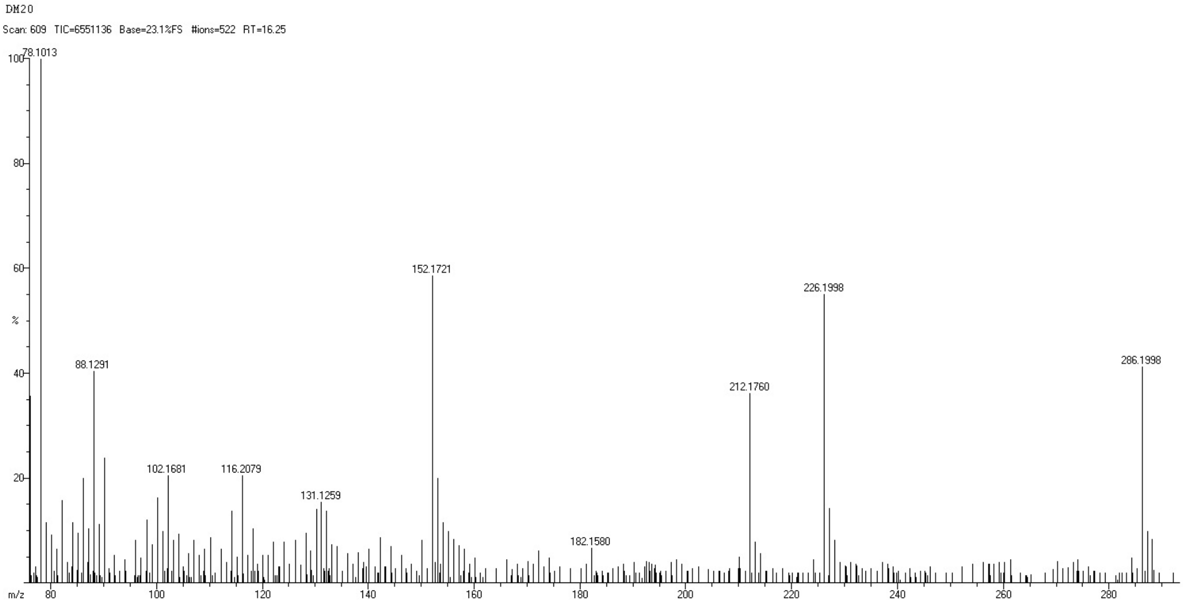
DM20
Scan: 609 TIC=6551136 Base=23.1%FS #ions=522 RT=16.25
78.1013
88.1291
102.1681
116.2079
131.1259
152.1721
182.1580
212.1760
226.1998
286.1998
%
m/z

Text S1. Supplementary procedure for toxicity evaluation

Cell culture

The cell line of Vero (African green monkey kidney cells) was procured from NCCS (Pune, India) and was grown in liquid medium (DMEM) containing 100 ug/mL penicillin, 100 μg/mL streptomycin, and 10% Fetal Bovine Serum (FBS) and preserved under an atmosphere of 5% CO_2 at 37^oC.

MTT Assay

The **DM2** was assayed for *in vitro* cytotoxicity by MTT assay using the cultured Vero cells. Briefly, the cultured Vero cells were produced by cell dissociation with trypsin (trypsinization), collectively in a 15 mL tube. At a density of 1×10^5 cells/mL cells/well (200 μL), the cells were plated for 24-48 h at 37°C into 96-well tissue culture plate in DMEM medium containing 10 % FBS and 1% antibiotic solution. The wells were washed with sterile phosphate buffered saline (PBS) and further allowed to react with varying concentrations of the **DM2** in a serum free DMEM medium. The samples were triplicated and the cells were kept for incubation at 37°C for 24 h in a humidified 5% CO_2 incubator. MTT (20 μL of 5 mg/mL) was added into each well after the incubation and the cells were incubated for another 2-4 h. The end point was determined by the purple precipitation, which was clearly seen under an inverted microscope. At the end, the medium along with MTT (220 μL) were used for the aspiration of the wells and later washed with 1X PBS (200 μL). In order to dissolve formazan crystals, DMSO (100 μL) was added to the plate with shaking for 5 min. The absorbance at 570 nm was measured using a micro plate reader (Thermo Fisher Scientific, USA). The IC_{50} value and the percentage cell viability was calculated using GraphPad Prism 8.0 software (USA).

Table S1. Inhibitions of AChE, BChE, and BACE-1 by **DM** series[a]

**Compounds**	**Residual activity at 10 μM (%)**		
	**AChE**	**BChE**	**BACE-1**
**DM1**	75.60 ± 2.35	93.21 ± 0.30	92.67 ± 0.26
**DM2**	71.91 ± 2.87	64.16 ± 0.44	100.50 ± 0.18
**DM3**	81.47 ± 4.09	69.87 ± 0.19	99.67 ± 0.53
**DM4**	74.23 ± 2.43	80.07 ± 1.59	90.82 ± 0.09
**DM5**	71.15 ± 6.12	60.54 ± 0.03	87.80 ± 0.29
**DM6**	73.04 ± 0.75	79.72 ± 1.10	88.53 ± 0.22
**DM7**	87.23 ± 1.19	98.21 ± 1.01	97.29 ± 0.34
**DM8**	82.69 ± 10.41	85.42 ± 0.67	91.01 ± 0.06
**DM9**	71.92 ± 3.06	72.50 ± 3.69	88.20 ± 0.28
**DM10**	64.26 ± 6.85	92.84 ± 0.51	87.13 ± 0.42
**DM11**	80.34 ± 5.21	80.53 ± 1.36	97.51 ± 1.02
**DM12**	68.52 ± 2.87	63.48 ± 1.47	94.27 ± 1.06
**DM13**	95.00 ± 3.40	100.38 ± 7.32	99.86 ± 0.28
**DM14**	97.58 ± 3.42	76.53 ± 1.37	99.83 ± 0.81
**DM15**	83.89 ± 4.91	55.16 ± 1.01	99.54 ± 0.24
**DM16**	82.70 ± 9.21	91.21 ± 1.43	99.74 ± 0.04
**DM17**	81.78 ± 6.31	68.89 ± 1.57	100.03 ± 0.04
**DM18**	78.55 ± 4.89	59.55 ± 4.08	99.94 ± 0.01
**DM19**	70.00 ± 2.02	103.45 ± 8.13	99.86 ± 0.12
**DM20**	86.98 ± 0.45	93.10 ± 6.50	99.89 ± 0.08
Donepezil[b]	0.010 ± 0.002	0.180 ± 0.004	-
Quercetin[c]	-	-	13.400 ± 0.035

[a] Results are the means ± standard errors of duplicate or triplicate experiments.
[b] IC_{50} of reference compounds for AChE and BChE.
[c] IC_{50} of reference compound for BACE-1.

The AChE and BChE inhibitory activities were measured by continuous colorimetric determination method [64], in the presence of 0.5 mM acetylthiocholine iodide (ACTI) and butyrylthiocholine iodide (BTCI) as substrates, respectively, for 15 min at 412 nm using UV/Vis spectrophotometer (OPTIGEN POP, K LAB, Daejeon, Korea) [65,66]. After adding the substrate, 0.5 mM 5,5-dithiobis (2-nitrobenzoic acid) (DTNB) was added as the color development solution. The BACE-1 inhibitory activity was evaluated by β-secretase (BACE1) activity detection kit (Sigma-Aldrich, St. Louis, Mo, USA) using a fluorescence spectrometer (FS-2, Scinco, Seoul, Korea) [67].

References

64. Ellman, G.L., Courtney, K.D., Andres, V. Jr., Feather-Stone, R.M., 1961. A new and rapid colorimetric determination of acetylcholinesterase activity. *Biochem. Pharmacol.* 7, 88-95.
65. Lee, J.P., Kang, M.-G., Lee, J.Y., Oh, J.M., Baek, S.C., Leem, H.H., Park, D., Cho, M.-L., Kim, H. 2019. Potent inhibition of acetylcholinesterase by sargachromanol I from *Sargassum siliquastrum* and by selected natural compounds. *Bioorg. Chem.* 89, 103043.
66. Heo, J.H., Eom, B.H., Ryu, H.W., Kang, M.-G., Park, J.E., Kim, D.Y., Kim, J.H., Park, D., Oh, S.R., & Kim, H., 2020. Acetylcholinesterase and butyrylcholinesterase inhibitory activities of khellactone coumarin derivatives isolated from *Peucedanum japonicum* Thurnberg. *Sci. Rep.* 10, 21695.
67. Abdelgawad, M.A., Oh, J.M., Parambi, D.G.T., Kumar, S., Musa, A., Ghoneim, M.M., Nayl, A.A., El-Ghorab, A.H., Ahmad, I., Patel, H., Kim, H., Mathew, B., 2022. Development of bromo- and fluoro-based α, β-

unsaturated ketones as highly potent MAO-B inhibitors for the treatment of Parkinson's disease. *J. Mol. Struct*. 1266, 133545.
